# The *N*-Alkylation of Agelastatin A Modulates Its Chemical Reactivity

**DOI:** 10.3390/molecules28196821

**Published:** 2023-09-27

**Authors:** Michele D’Ambrosio

**Affiliations:** Laboratory of Bioorganic Chemistry, Department of Physics, Università degli Studi di Trento, Via Sommarive 14, 38123 Trento, Italy; michele.dambrosio@unitn.it

**Keywords:** agelas, agelastatin, pyrrole-imidazole alkaloid, *N*-acyliminium ion, electrophilic aromatic substitution, debromination, anticancer

## Abstract

Agelastatin A is a marine alkaloid with potent biological activity. To date, at least 17 different strategies have achieved its total synthesis, along with many analogues. The present study focuses on the acidity stability of some *N*-methyl derivatives of agelastatin A. The study made use of chemical reactions and spectroscopic acquisitions. The chemical structure of some derivatives can undergo a profound rearrangement. The results could shed light on the mechanism of action of agelastatin A and suggest the preparation of analogues with improved pharmacological efficacy.

## 1. Introduction

**Scheme 1:**
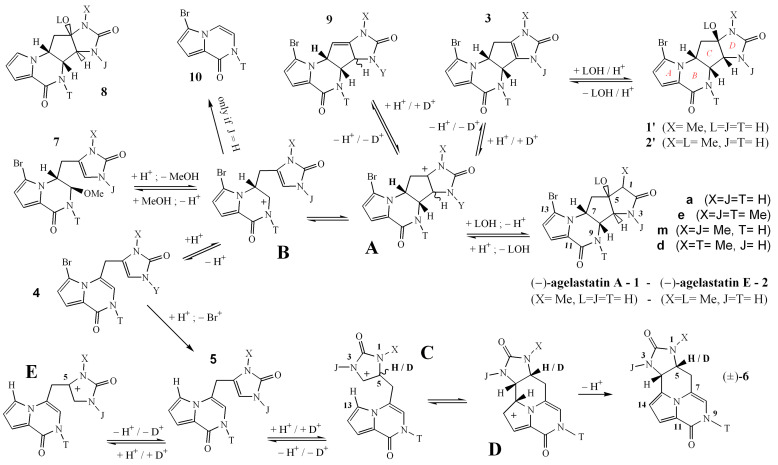
Plausible pathways of equilibria involving agelastatins and reactions to give compounds **4**, **5** and **6**. *N*-acyliminium ions (NAI) (**A**–**E**) are reaction intermediates.

Agelastatin A **1** (Scheme 1) is a specialised metabolite isolated for the first time from the axinellid sponge *Agelas dendromorpha* from the Coral Sea [1,2]. Its structural formula places it among the pyrrolo-imidazol alkaloids because it exhibits four condensed cycles (C4N1-C5N1-C5-C3N2) and four contiguous stereocentres.

Seventeen research groups have achieved total synthesis of agelastatin A, some through different routes. Synthetic efforts have been reviewed [3,4,5]. The pyrrole ring *A* was generally obtained from commercially available pyrrol-2-carboxylic acid and rarely by condensation of a primary amine with a 1,4-dicarbonyl compound [6,7,8,9]. Most synthetic strategies have focused on early *C* carbocycle formation with appropriate vicinal diamine functionality and stereochemistry. Two biomimetic strategies were both based on the reactivity of the iminium ion; they involved the sequential formation of the *C* and *B* rings [10] or the formation of the *C* ring in the final step [7]. The last route provided 1.4 g of (−)-agelastatin A **1** in a single batch (8 steps, 22% overall yield, 96% ee). The shortest route exploited the chemistry of Stenhouse salts, which provided the carbocycle in a single step [11]. This procedure has recently been improved to provide (±)-agelastatin A in 6 steps and 26% overall yield [12]. The success of total syntheses has allowed the preparation of many derivatives and the execution of biological assays. SAR studies have highlighted that intact functional groups (N3-H, C4-H, N9-H, C15-H) are essential for cytotoxicity [2,13,14,15,16], N1-ethyl is slightly tolerated [17] while C5-OMe was effective in one case [18]. Only 13-chloro and 13-trifluoromethyl substituents revealed bioactivity greater than **1** against some tumour cell lines [17,19]. Agelastatin A targets the peptidyl transferase centre of the eukaryotic ribosome, thereby inhibiting tumour cell proliferation [20]. The contribution of computational studies is also interesting [21]. 

The present study adopts an arbitrary numbering to designate the many agelastatin derivatives (Figure S1). Compound **2** was easily obtained by stirring **1** in methanol under reflux in the presence of the heterogeneous acid catalyst Amberlyst^®^ 15 (A15) [1]. Later, compound **2** was isolated as a natural metabolite and named agelastatin E [22]. This transformation is reversible, and the hydroxyl group can be reinstated simply by changing the solvent in the water/acetone 1:2 mixture and A15 [2]. 

Compound **2** eliminates the methanol when heated under reflux in dry pyridine to give compound **3**. This transformation is also reversible so that **1** or **2** can be recovered when **3** is stirred in water or methanol, and the resin is strongly acidic. However, the addition of water or methanol to the C4=C5 double bond in **3**, can also furnish the stereoisomers with the inverted configuration at C4 and C5, i.e., the **1’** and **2’** compounds [2]. All of these interconversions occur through the common carbocation intermediate **A** (Scheme 1).

In conclusion, **1**, **2** and **3** can be considered as the progenitor compounds of three series of agelastatin analogues, which differentiate by substituents at the nitrogen atoms and, for **1** and **2**, also by stereochemistry at the C4–C5 bond.

If, for simplicity, we consider that N-substituted compounds carry an identical moiety, eight compounds can be envisaged: one without substituents, three mono-substituted, three di-substituted and one completely substituted. Furthermore, taking into account that **1** and **2** have a methyl group bonded to heteroatoms, we focus on the methyl moiety. 

Agelastatin D **1a** was isolated from the Indian Ocean sponge *Cymbastela* sp. [23] and shows three NH functional groups, so all methyls are absent. Of the seven possible combinations of methyl groups bonded to the three nitrogen atoms, three have so far been described in the literature: the N1-methylated agelastatin A **1** and the entirely methylated **1e**, together with the analogues of series **2e**, **2’e** and **3e** [2] and the di-substituted at up-down (N1,N9 nitrogens) positions **1d** (Scheme 1) [14].

Starting from agelastatin A **1**, we have here prepared the di-substituted at the up-medium (N1,N3 nitrogens) positions **1m** (Scheme 1). Like agelastatin A **1**, compounds **1m** and **1d** can be easily converted into the **2m/d** and **3m/d** series; no attempt has been made here to isolate the **1’m/d** and **2’m/d** series of epimers. The remaining N3 or N9 mono-methylated and the N3,N9-di-methylated agelastatins would be accessible only by total synthesis (Figure S1). 

In a past experiment, the permethylated agelastatin A **2e**, dissolved in chloroform and heated in the presence of A15 beads, yielded many products that could be separated by thin layer chromatography (TLC) on silica gel. Two minor products were tentatively identified as **4e** and **5e,** while one major product was characterised as **6e** (Scheme 1). Compound **4e** was supposed to be produced by loss of methanol and opening of the carbocycle; compound **5e** would have to undergo further reductive debromination. In fact, **6e** showed a new ring formed by the C4–C13 closure. Compounds **4e** and **5e** are devoid of stereogenic carbon, and, actually, **6e** has measured zero rotatory power, being a racemic mixture. Movassaghi reported similar cyclisation when treating pre-agelastatin D **7a** with methansulphonic acid (MsOH) in water to give **6a** [7,13]. He suggested a protodebromination reaction and did not delve into the study of reaction mechanisms. 

These transformations look quite interesting and are worth studying in terms of solvent, temperature, type and mole ratio of an acid catalyst. Furthermore, the present study aims to investigate how the electron-donating group methyl at the nitrogen positions N1, N3 and/or N9 affects the reactivity of agelastatin A.

## 2. Results and Discussion

### 2.1. Reaction Conditions and Products

The permethylated agelastatin A **2e** (~5 mg) was stirred under reflux with 2–3 equivalents of MsOH in CHCl_3_, CH_2_Cl_2_, CH_2_ClCH_2_Cl (12 h) and acetone (48 h). Chlorinated solvents exert only Van der Waals forces and give the best results. Yields of **6e** seem to be affected by the boiling temperature so that it is 50% in CH_2_Cl_2_ and 100% in CHCl_3,_ while some unidentified decomposition products appear in DCE. After refluxing **2e** in acetone for 48 h and neutralisation, the reaction mixture contains **1e**/**3e**/**6e**~4:2:1. Reflux is not necessary, but **6e** achieved approximately 90% yield after 6 days at room temperature. In addition to MsOH, pTsOH and dry HCl and HBr (obtained by in situ hydrolysis of AcCl and PrCOBr) also partially catalysed the conversion of **2e** into **6e**. Trifluoroacetic acid cleanly produced the **3e** olefin. Without strong acid, no reaction takes place. MsOH turned out to be the preferred catalyst. 

Very interesting were the results on the stoichiometric addition of MsOH. Compound **6e** is formed quantitatively by the addition of at least one acid equivalent. The addition of 0.1 equivalent of MsOH gives mainly **3e,** which slowly adds water and\or methanol. The addition of 0.5 equivalent of MsOH gave a mixture of **1e**/**3e**/**4e**/**6e**/**5e**~40:40:10:8:2, which, after two weeks, was shown to contain only **1e** and **6e** to the proton spectrum.

Evidence suggested that the reaction should be run with less than one acid equivalent for a short time and then with rapid separation to capture a small amount of **4e** and **5e**. An experiment was carried out on **2e** in larger quantities, and the solution refluxed for 15’. After neutralisation, the crude product was subjected to flash chromatography (FC) on SiO_2_ eluting with a gradient of 2-propanol (iPrOH) in acetone. Final fractions contained **3e**/**4e**/**5e**~3:3:1. After one week, the NMR tube showed compounds **1e**/**3e**/**4e**/**5e**~1.5:1.5:3:1, and after one-month compounds **1e**/**6e**~3:4. Therefore, several experiments have shown that the **3e** olefin easily adds methanol (or water) to the double bond to give **1e** while **4e** and **5e** decompose or transform into **6e**. 

The procedure described above was repeated: FC supplied the largely unreacted **2e**, small amounts of **6e** and **3e** and traces of **1’e** in the central fractions. The final fractions contained **3e**/**5e**/**4e**~4:2.5:1 and were separated by HPLC. Three major peaks were collected at the retention time (**t_R_**) 9’ (**3e** + **1’e**), 15’ (**4e**) and 19’ (**5e**). Each compound weighed less than 1 mg. Compound **4e** was highly reactive, probably due to the unfavourable conformation and the onset of steric strain between the C13 bromine and C6 methylene following the formation of the C7=C8 double bond [7]. In fact, **4e** undergoes a protodebromination, providing the compound **5e**, which easily rearranges to give **6e**. For the above reasons, the accumulation of compounds **4e** and **5e** proved prohibitive and hampered the complete acquisition of spectroscopic data for **4e**. However, an LC-MS study of four reactions and comparison of retention times, proton NMR, DAD-UV and low-resolution MS spectra (Table 1) strongly support the proposed structure for **4e** (Figure S6m).

Despite having very close **t_R_**, similar UVs and the same MW, it is possible to distinguish **6e** from **5e** with certainty from their fragmentation spectra MS^2^ (Figure S8f,h).

### 2.2. Reaction Mechanism

The results shown so far suggest that the isolated compounds rearrange as depicted in Scheme 1. Compounds **1**, **2**, **3**, **1’** and **2’** interconvert with each other via the common intermediate carbocation **A**. This equilibrium favours the natural *cis*-transoid-*cis* ring junction. In fact, pure **2’e** spontaneously gives rise to a mixture of **2’e**/**3e**/(**1e** + **2e**)~1.5:1.5:(4). 

However, the carbocation intermediate **A** can also break the C4–C8 bond to give **B** and allow compound **4e** to be obtained by the elimination of H7. The isolation of **4e** and **5e** suggests that the **6e** formation occurs via two distinct reactions: the initial protodebromination (**4e** → **5e**) is followed by C4–C13 cyclisation (**5e** → **6e**). Two further experimental data (Exp 1 and 2) support that the rearrangement occurs in two distinct reactions, while two mechanistic considerations (Mec 1 and 2) invalidate the hypothesis of a single reactive event:

Exp 1. It is not necessary for the bromine to be bound to C13 because a pure sample of **5e** converts to **6e** with acid catalysis (Section 2.3).

Exp 2. Sodium hydride reduces **2e** by nucleophilic attack at C13 and removal of the bromide, thus forming **8e**. A pure sample of this derivative, dissolved in CHCl_3_ and spiked with catalytic MsOH, produced **6e**.

Mec 1. Since C4 is nucleophilic in compounds **4e** and **5e**, it could bind to the electron-poor C13 because it is delta to the C10 carbonyl. Subsequent detachment of the bromide anion from C13 would create a C5 carbocation, which requires a hydride ion to give rise to the neutral product **6e**. This description of rearrangement through a single reaction event is not very convincing, given the acidic environment of the reaction.

Mec 2. Furthermore, the voluminous bromine atom can cause a steric hindrance towards both the nucleophilic or electrophilic attack of C4.

In conclusion, the most plausible mechanism of **6e** formation appears to consist of two consecutive electrophilic aromatic substitution reactions. Building on Horne’s work [24], numerous experiments were performed to obtain clues about the mechanism of protodebromination and the fate of the bromonium ion. Unfortunately, no useful result was obtained. Certainly, some oxidative degradation products of **1** are formed whose structure has not been elucidated. Among the halogens, bromine appears to have unique chemical properties that make electrophilic aromatic bromination reversible [25], similar to sulfonation. Elucidating bromination conditions requires dedicated study, selection of substrates and use of the proper equipment. 

### 2.3. Deuterium Incorporation: MS and ^13^C-NMR Studies

Since C4–C13 annulation begins with protonation at C5, it has been useful to perform the reaction with deuterated methanesulfonic acid (MsOD). Two experiments with MsOD catalysis were performed on the **2e** and **5e** substrates. The first reaction gave, after TLC, two bands corresponding to deuterated **5e** (2e) and **6e** (2e). The second reaction produced deuterated **6e** (5e). 

**Figure 1:**
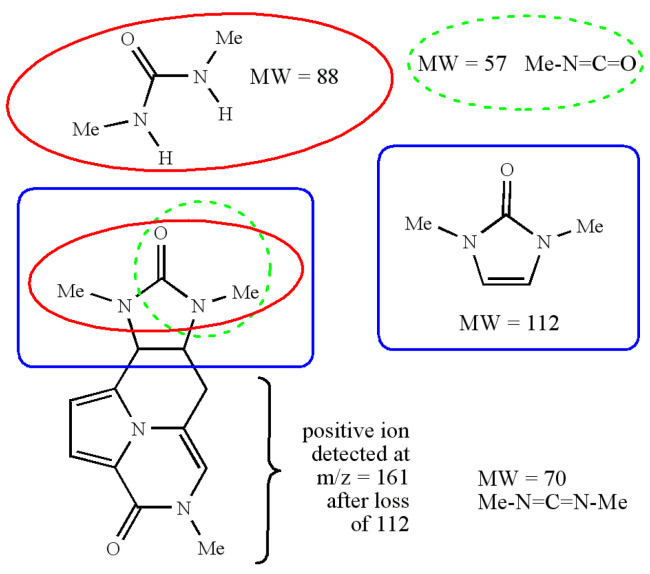
Fragmentation pattern of **6e** as inferred from HR(ESI+)MS measurements.

The ESI-(+)-MS spectra of the three products always showed an ion cluster whose lowest value, at *m*/*z* 273, corresponded to the [M + H]^+^ ion with a molecular formula containing only protons. The other ions in the cluster had *m*/*z* values increasing by one unit, each corresponding to the introduction of one or more deuterium atoms in the molecular formula. The molecular ions in each cluster were five for **6e** (2e), four for **5e** (2e) and three for **6e** (5e). Subsequently, each ion in the cluster was isolated in the MS trap and subjected to fragmentation. The fragmentation pattern observed on the [M + H]^+^ of **6e** (2e) is depicted in Figure 1.

The fragment ion at *m*/*z* 161 stands out as a diagnostic ion in that it corresponds to the pyrrolopyrazinone moiety after loss of the neutral imidazolone moiety (112 Dalton). In fact, this fragmentation allows for discrimination of the deuterium incorporated at C13, C14, C15, and C6 (pyrazinone ion) from those at C4 and C5 (neutral imidazolone loss). The H/D substitution on the pyrrole ring can occur at any time and on all compounds in Scheme 1. Incorporation at C6 can be explained by assuming an equilibrium **A** ⇆ **9**. Deuterium atoms can enter C4 due to the equilibria **3** ⇆ **A** ⇆ **1’** or even **5** ⇆ **E**. However, incorporation at C5 can only occur by the equilibrium **5** ⇆ **C,** which gives the final product **6**. A thorough evaluation of the MS^2^ data is explained in the Supplementary part. The complete data are reported in Figure S8i, and their in-depth evaluation is explained in Figure S8j. Consequently, the deuterated imidazolone in **6e** (2e), **5e** (2e) and **6e** (5e) amounts to 48.3%, 15.4% and 21.5%, respectively. Partial incorporation of deuterium is likely due to residual moisture in the reaction flask. The residual moisture was greater when the MsOD-catalysed reaction was performed on the **5e** substrate than on **2e**. However, the 33% percentage difference (48 minus 15) is due to the addition of D to C5. This supports the proposed mechanism, consisting of two consecutive electrophilic aromatic substitution reactions. 

Simultaneous examination of the DEPT-135 and ^13^C-NMR spectra of **6e** (2e) showed a good signal-to-noise ratio for the three methyls and the mono-substituted olefinic carbons C8 and C15 as well as for the two carbonyls C2 and C10. A poor signal-to-noise ratio appeared for the C4 doublet and C6 triplet, while the C7, C11 and C13 singlet olefin carbons barely emerged from the baseline due to their long relaxation time (and perhaps also their quadrupolar coupling with N12). Interestingly, the C5 and C14 hydrogen-bonded carbon signals exhibited a broadening and multiplicity that can only be attributed to coupling with deuterium instead of protium. These data point out that the ^2^H isotope is mainly located in the C14 position of the pyrazinone ion and C5 of the imidazolone neutral loss, which confirms the above reasoning regarding the 33% difference.

### 2.4. N-Alkylations and Reaction Products

The carbocation **B** is a common intermediate obtainable from both alkylated agelastatin A and pre-agelastatin. Indeed, Movassaghi had previously reported the formation of **6a** (20% yield) starting from pre-agelastatin D **7a** together with **1a** (26%), **2’a** (9%) and **10a** (20%) [7]. However, starting from pre-agelastatin A **7**, he obtained only **1** (49%) and **2’** (22%). We wondered whether the electron donor property of methyl groups could selectively drive the C–C4 or C4–C13 cyclisation (after protodebromination). 

Two minor products of the methylation reaction of agelastatin A were **1m**, **2m**, **1d** and **2d**. A chromatographic separation yielded **1d** and **1m** pure samples. However, to address the shortage of **1d** and **1m**, the reactions were performed in the NMR tube, and the transformation was followed spectroscopically. 

A sample of **2e** was used to refine the reaction conditions: it was dissolved in CDCl_3,_ and MsOH was added in portion until the signals of alkaloid methyls and acid methyl were integrated roughly equal. The NMR tube was warmed at 62 °C for 1 h to observe that all **2e** had transformed into **6e**. No clear signal emerged that could be attributed to **3e**, **4e** and **5e**. The NMR signals showed a downfield shift when in an acidic solution but moved to the usual shift after neutralisation with basic beads of Amberlyst^®^ 21 (A21). The experiment was repeated twice and gave the same results. 

A sample of **1m** was treated similarly: after two hours of heating, most of the **1m** had turned into **3m**. The sample was allowed to cool at r.t., observing that it again contained **1m**. Repeated heating and cooling changed the head between **3m** and **1m**, respectively. The experiment was repeated twice and gave the same results. 

A sample of **1d** was treated in a similar way: the NMR tube was heated at 65 °C for 30’ to observe that all **1d** had turned into **3d**. The heating was continued for 2 h, during which the signals for **3d** slowly decreased. After neutralisation with beads of A21, a set of signals prevailed that is definitely attributable to **5d**. Minor sets of signals could not be identified. The experiment was repeated twice with **1d** and once with **2d**, always giving the same results.

All products were analysed by ESI-(+)-MS spectrometry, and the acquired data confirmed the ^1^H-NMR identification. An LC-MS study revealed that **6d** formed in traces starting from **2d**, as evidenced by their different **t_R_** and MS^2^ fragmentation of identical [M + H]^+^ at *m*/*z* 273. 

These results can be rationalised in a simple way. *N*-acyliminium ions (NAI) are formed by fragmentation of an ether emiaminal amid via an alcohol leaving group or by protonation of a *N*-acyl enamide (NAE) at the β-nitrogen olefin carbon [26]. Both methods require a strong acid medium. The first method provides NAI intermediates **A** (from **1**/**2** or **1’**/**2’**) and **B** (from **7**). Unusually, if **B** comes from **A**, the leaving group is an electron-rich carbon atom instead of the usual alcohol. The second method gives rise to NAI intermediates **C** (from **5**) or **A** (from **3** or **9**). The second method exploits the basic behaviour of an NAE that can be protonated at the β-nitrogen olefin carbon. 

**Figure 2:**
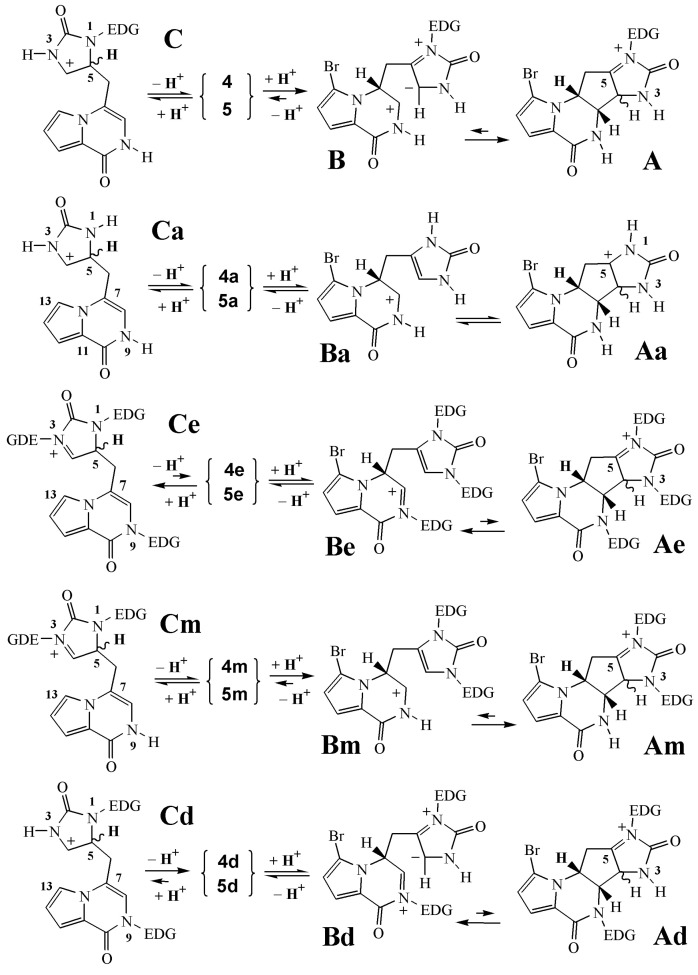
Resonance structures of *N*-methyl-*N*-acyliminium ions. The electron-donor alkyl groups shift the equilibria among the NAIs.

In general, an electron-donating group (e.g., methyl) stabilises a positive charge. Therefore, *N*-alkylated NAIs are more stable than hydrogenated ones. An *N*-alkylated NAI forms more easily, as effectively as the leaving group, and is less electrophilic than a hydrogenated one [27]. However, it must be kept in mind that NAEs also behave as nucleophiles. The above theoretical statements are applied to the known experimental examples below (Figure 2). 

Pre-agelastatin A **7** gives rise to iminium ion **B,** which has a high internal energy and is highly electrophilic. The C4 position is the most nucleophilic, among other things, because the electron donor methyl is bonded to N1 instead of N3. Consequently, the C8–C4 cyclisation takes place rapidly, in high yields (**1** + **2’** = 71%) [13] and without minor by-products. The back opening of the carbocycle is not favoured because it involves the transition from the *N*-alkylated NAI ion **A** to the hydrogenated NAI ion **B**. 

Pre-agelastatin D **7a** forms the corresponding iminium ion **Ba**. However, here, C4 is poorly nucleophilic, and the C8–C4 cyclisation occurs slowly so that the H7 elimination process becomes competitive. This elimination should form **4a** and open the way to **6a**. Furthermore, the breaking of the C7–C6 bond is competitive with the protodebromination reaction, thus producing **10a**. 

The permethylated agelastatin A **2e** readily loses methanol to form the iminium ion **Ae,** which can generate equilibria leading to compounds **3e** and **2’e**. Alternatively, rearrangement to the iminium ion **Be** may also occur. In fact, its formation would be thermodynamically favoured by the release of the pentacarbon ring strain and, specifically, by the generation of a more stable, low internal energy, iminium ion **Be**, which is alkylated at the N9 position. The back reclosure from **Be** to **Ae** could also be slowed down by the homogeneous distribution of electrons in the C4=C5 double bond induced by alkylation at both N1 and N3 nitrogen atoms. 

A similar reasoning to that of pre-agelastatin A applies to derivative **1m**. In fact, the equilibrium between the intermediate ions, **Bm** and **Am**, is largely shifted toward **Am** because the NAI **Bm** lacks the electron donating methyl at N9. As a result, with **1m,** the N1 alkylated NAI **Am** does not undergo any breakage of the C4-C8 bond. 

A similar reasoning to that of **2e** holds for the derivative **1d**. In fact, the iminium ion **Bd** can be formed thanks to its stabilisation by methyl at N9. After H7 elimination and protodebromination, compound **5d** is mainly obtained. In essence, the iminium ion **Cd** is not formed because the electron donor methyl at N3 is missing. Consequently, the reaction process stops at compound **5d**, providing only traces of the **6d** analogue.

## 3. Materials and Methods

### 3.1. Chemicals and Instrumentation

Analytical grade solvents were used for extraction procedures and reactions. Reactions were carried out under N_2_, the glassware was heat-dried, and THF was distilled from Na/benzophenone prior to use when dry conditions were required. Commercially available chemicals were usually used without further purification. Yields are given on reacted substrates; however, no attempt at yield optimisation was made. For flash chromatography, commercial silica gel VWR Normasil 60 (40–63 μm) and Merck RP-18 LiChroprep^®^ (40–63 μm) were used. Precoated silica gel plates (Merck Kieselgel 60 PF254) were used for analytical TLC. Preparative HPLC: Merck Hitachi system equipped with an L7100 pump, an L7400 UV detector, a D7500 integrator and a Rheodyne manual injector. Analytical HPLC: Agilent 1100 series LC system consisting of a binary pump, a vacuum degasser, an autosampler with a standard analytical head (100 μL), a column thermostat and a 1200 series diode array detector (DAD). The column oven temperature was fixed at 25 °C; the flow rate was 1.0 mL/min, and the injection volume was 10 μL; the DAD was set at 220, 254 and 289 nm. The following HPLC columns were used: (i) Kinetex^®^ C18 (150 × 10 mm, 5 μm, 100 Å, Phenomenex, Torrance, CA, USA); (ii) Kinetex^®^ C18 (250 × 4.6 mm, 5 μm, 100 Å, Phenomenex, Torrance, CA, USA); (iii) Luna SiO_2_ (150 × 3 mm, 3 μm, 100 Å, Phenomenex, Torrance, CA, USA). UV: Perkin-Elmer Lambda3 (λ_max_ in nm, ε in mol^−1^ l cm^−1^). NMR: Varian XL-300 (^1^H at 299.94 MHz, ^13^C at 75.4 MHz) or Bruker Avance 400 (^1^H at 400 MHz, ^13^C at 100 MHz), 5 mm probe. Chemical shifts are reported in ppm (δ) using residual solvent signals as internal standard (CDCl_3_: δ_H_ = 7.26, δ_C_ = 77.0; CD_3_OD: δ_H_ = 3.31, δ_C_ = 49.0). Coupling constants (*J*) are in Hz, multiplicities and peak assignments from DEPT, ^1^H,^1^H-COSY, ^1^*J*_CH_-and ^n^*J*_CH_-heterocorrelations, ^1^*J*_CH_ (HSQC), ^n^*J*_CH_ (HMBC) and NOESY experiments. High-resolution mass spectra: Kratos MS80 spectrometer with home-built acquisition data system; electron impact ionisation at 70 eV; LTQ-Orbitrap hybrid mass spectrometer (Thermo Fisher Scientific, Bremen, Germany) fitted with an electrospray source (ESI) operating in positive ionisation mode. LC-MS analyses: Agilent 1100 series LC system interfaced to a Bruker model Esquire multiple ion trap mass spectrometer equipped with an atmospheric pressure interface electrospray (API-ES) chamber.

### 3.2. Reaction Conditions and Products

#### 3.2.1. Rel-(6aR,9aR)-4,7,9-trimethyl-4,6,6a,7,9,9a-hexahydroimidazo [4,5-g]pyrazino [2,1,6-cd]indolizine-3,8-dione (**6e**)

General procedure for preparing **6e**: A solution of **2e** (~5 mg, 13 μmol) in 5 mL of the selected solvent and 1–3 equivalents of acid catalyst was refluxed for 12–48 h. The mixture was then neutralised by the addition of A21 or Na_2_CO_3_, filtered, dried and the crude product dissolved in a deuterated solvent. Proton NMR spectra allowed the identification of reaction products and their relative conversion rate. 

Data: **6e**: ^1^H NMR (300 MHz, CDCl_3_) δ 7.11 (d, J = 4.0, H-15), 6.61 (dd, J = 4.0, 0.5, H-14), 6.28 (dd, J = 1.8, 0.8, H-8), 4.56 (br.d, J = 6.8, H-4), 3.80 (ddd, J = 9.5, 6.8, 4.9, H-5), 3.45 (s, CH_3_-9), 3.06 (ddd, J = 15.2, 4.9, 0.8 Ha-6), 2.90 (s, CH_3_-1), 2.86 (s, CH_3_-3), 2.77 (dddd, J = 15.2, 9.5, 1.8, 0.6, Hb-6). ^13^C NMR (75 MHz, CDCl_3_) δ 160.5 (C-2), 155.8 (C-10), 122.8 * (C-13), 121.4 * (C-11), 115.3 (C-8), 113.1 (C-14), 112.4 (C-7), 110.1 (C-15), 52.4 (C-4), 51.8 (C-5), 35.0 (CH_3_-N9), 29.5 (CH_3_-N3), 28.7 (CH_3_-N1), 24.3 (C-6); UV (EtOH) λ_max_ 233 (19500ε), λ_max_ 280 (5500ε); HRMS (ESI): Calcd for C_14_H_17_N_4_O_2_ [M + H]^+^ 273,13460, found: 273.13449; Calcd for C_14_H_17_N_4_O_2_ [M + H]^+^ 273,13460, found: 273.13622; Calcd for C_14_H_16_N_4_O_2_Na [M + Na]^+^ 295,11655, found: 295.11798. HRMS (EI): Calcd for C_14_H_16_N_4_O_2_ [M^+^] 272.12732, found: 272.12708. 

General procedure for the preparation of **3e**, **4e**, and **5e**. In a dry flask, a solution of **2e** (44 mg, 0.115 mmol) in 8 mL of CHCl_3_ and 1 equivalent of MsOH was refluxed for 15 min. The mixture was then neutralised over sodium carbonate. The solvent was evaporated to dryness, and the residue was purified by FC on silica gel. The eluent Me_2_CO/i-PrOH 80:20 gave the products **2e** > **6e** > **3e** > **1’e**; the eluent Me_2_CO/i-PrOH 60:40 gave the products **3e** > **5e** > **4e**. The latter fraction was separated by HPLC over the SiO_2_ column, eluting with hexane/ethanol 70:30 and collecting the peaks of (**3e** + **1’e**) at t_R_ = 8.9, **4e** at t_R_ = 15.0 and **5e** at t_R_ = 18.7.

#### 3.2.2. (5aR,9aR)-1-Bromo-5,6,8-trimethyl-5,5a,6,8,9,9a-hexahydroimidazo [4’,5’:4,5]cyclopenta [1,2-e]pyrrolo [1,2-a]pyrazine-4,7-dione (**3e**)

Data for **3e**: ^1^H NMR (400 MHz, CDCl_3_) δ 6.94 (d, J = 4.0, H-15), 6.32 (d, J = 4.0, H-14), 5.12 (m, H-7), 4.95 (dd, J = 6.2, 1.2, H-8), 3.37 (s, CH_3_-9), 3.30 (dd, J = 14.3, 7.1 Ha-6), 3.25 (s, CH_3_-1), 3.05 (s, CH_3_-3), 2.72 (ddd, J = 14.3, 7.3, 1.2, Hb-6). ^13^C NMR (75 MHz, CDCl_3_) δ 156.0 (C-10), 154.6 (C-2), 125.3 (C-5), 123.3 (C-11), 120.0 (C-4), 114.8 (C-15), 113.2 (C-14), 57.4 (C-8), 56.5 (C-7), 31.0 (C-6), 30.2 (CH_3_-N9), 30.0 (CH_3_-N1), 29.1 (CH_3_-N3); See also [2].

#### 3.2.3. 6-Bromo-4-[(2,3-dihydro-1,3-dimethyl-2-oxo-1H-imidazol-4-yl)methyl]-2-methylpyrrolo [1,2-a]pyrazin-1(2H)-one (**4e**)

Data for **4e**: ^1^H NMR (400 MHz, CDCl_3_) δ 7.18 (d, J = 4.2, H-15), 6.59 (d, J = 4.2, H-14), 6.00 (br.s, H-8), 5.87 (br.s, H-4), 4.19 (br.s, 2H-6), 3.40 (s, CH_3_-9), 3.25 (s, CH_3_-3), 3.24 (s, CH_3_-1).

#### 3.2.4. 4-[(2,3-Dihydro-1,3-dimethyl-2-oxo-1H-imidazol-4-yl)methyl]-2-methylpyrrolo [1,2-a]pyrazin-1(2H)-one (**5e**)

Data for **5e**: ^1^H NMR (400 MHz, CDCl_3_) δ 7.20 (dd, J = 4.0, 1.4, H-15), 7.10 (dd, J = 2.7, 1.4, H-13), 6.63 (dd, J = 4.0, 2.7, H-14), 6.05 (br.s, H-4 and H-8), 3.73 (br.s, 2H-6), 3.43 (s, CH_3_-9), 3.26 (s, CH_3_-3), 3.22 (s, CH_3_-1). ^13^C NMR (100 MHz, CDCl_3_) δ 155.9 (C-10), 153.9 (C-2), 124.6 (C-11), 116.7 (C-8), 116.2 (C-5), 115.5 (C-13), 115.1 (C-7), 113.0 (C-14), 110.9 (C-15), 110.0 (C-4), 34.9 (CH_3_-N9), 30.4 (CH_3_-N3), 27.7 (CH_3_-N1), 24.3 (C-6); HRMS (ESI+): Calcd for C_14_H_16_N_4_O_2_Na [M + Na]^+^ 295,11655, found: 295.11672.

### 3.3. Reaction Mechanism

Isolation of **2’e**: A mixture of reaction by-products was subjected to preparative HPLC over the C18 column, the eluents were CH_3_CN (A) and H_2_O (B), and A was applied in the gradient of 10% at t = 0, 25% at t = 30, 55% at t = 32 min, 55% at t = 35 min, 4.0 mL/min, λ = 289 nm; collecting peaks of **2’e** at t_R_ = 8.3, **3e** at t_R_ = 11.4 and **2e** at t_R_ = 13.4. Full spectroscopic data of **2’e** have been previously reported [2].

Preparation of **6e** from **8e**: A pure sample of **8e**, in 0.8 mL of CDCl_3_/CD_3_OD 95:5 and about one equivalent of MsOH, was refluxed for 4 h. The solvent was evaporated under vacuum, and the residue was dissolved in CDCl_3_ and heated under reflux for 3 h. Finally, the mixture was neutralised with A21, evaporated to dryness and dissolved in CDCl_3_. Its proton spectrum showed signals consistent with **6e**. A cluster of deuterated peaks was observed by the LC-MS experiment, thus confirming the NMR result (Figure S7e). 

### 3.4. Deuterium Incorporation

Preparation of **6e** (2e) and **5e** (2e) with methanesulfonic acid-*d*_4_: In a dry flask, a solution of **2e** (~14 mg, 36 μmol) in 7 mL of CDCl_3_ and approximately one equivalent of CD_3_SO_3_D, was heated at 60 °C for 90 min. The mixture was then neutralised over Na_2_CO_3_ and purified by TLC (CH_2_Cl_2_/iPrOH 9:1) to yield **6e** (t_R_ = 0.47, 2.7 mg, 27.8%) and **5e** (t_R_ = 0.23, 1.3 mg, 13.3%). A cluster of deuterated peaks was observed by MS experiments (Figure S8m,n). HR-MS data are in Figure S8e–h.

Preparation of **6e** (5e) with methanesulfonic acid-*d*_4_: A solution of **5e**, in 0.8 mL of CDCl_3_ and roughly one equivalent of CD_3_SO_3_D, was warmed at 60 °C for 90 min. The mixture was neutralised with A21: its proton spectrum showed signals consistent with **6e**. A cluster of deuterated peaks was observed by MS experiments (Figure S8k,l). 

### 3.5. N-Alkylations and Reaction Products

Preparation of **1d** and **1m**: A sample containing semi-pure agelastatin A **1** (~100 mg, ~0.3 mmol) was dissolved in dry THF (20 mL), and NaHMDA (330 mg, 1.8 mmol) was added under N_2_ atmosphere. The mixture was stirred for 30 min, and MeI (188 μL) was added. The reaction mixture was further stirred at r.t. for 1 h, and the solvent was evaporated. The residue was purified by FC over RP18, starting from MeOH/H_2_O 10:90 and applying a gradient of MeOH. Fractions eluted with (MeOH/H_2_O 40:60) gave **2e**, while those eluted with (MeOH/H_2_O 80:20) afforded a mixture of methylated derivatives. The crude products were dissolved in Me_2_CO/H_2_O 50:50 and stirred at r.t. for 48 h in the presence of A15. The residue was purified by HPLC over the C18 column to obtain **1** (t_R_ = 24.8, 2.5 mg), **1d** (t_R_ = 25.7, 3.5 mg), **1m** (t_R_ = 27.6, 18.5 mg) and **1e** (t_R_ = 28.7, 2.3 mg). The eluents were CH_3_OH (A) and H_2_O/CH_3_OH 95:5 (B), and A was applied in the gradient of 0% at t = 0, 40% at t = 40, 100% at t = 41 min, 100% at t = 45 min, 4.0 mL/min, λ = 254 nm.

#### 3.5.1. (5aS,5bS,8aS,9aR)-1-Bromo-8a-hydroxy-5,8-dimethyl-5,5a,5b,6,8,8a,9,9a-octahydroimidazo [4’,5’:4,5]cyclopenta [1,2-e]pyrrolo [1,2-a]pyrazine-4,7-dione (**1d**)

Data for **1d**: ^1^H NMR (400 MHz, CDCl_3_) δ 6.95 (d, J = 4.0, H-15), 6.29 (d, J = 4.0, H-14), 4.65 (m, H-7), 4.24 (br.s, H-4), 3.93 (d, J = 6.0, H-8), 3.15 (s, CH_3_-9), 2.83 (s, CH_3_-1), 2.60 (dd, J = 13.5, 6.4, Ha-6), 2.27 (dd, J = 13.5, 11.9, Hb-6); ^1^H NMR (400 MHz, CD_3_OD) δ 6.88 (d, J = 4.0, H-15), 6.32 (d, J = 4.0, H-14), 4.70 (m, H-7), 4.16 (br.s, H-4), 4.01 (d, J = 6.1, H-8), 3.13 (s, CH_3_-9), 2.80 (s, CH_3_-1), 2.66 (dd, J = 13.1, 6.5, Ha-6), 2.09 (dd, J = 13.2, 12.0, Hb-6); ^13^C NMR (75 MHz, CDCl_3_) δ 160.1 (C-10), 158.4 (C-2), 123.5 (C-11), 115.4 (C-15), 113.5 (C-14), 105.0 (C-13), 94.0 (C-5), 67.8 (C-8), 65.4 (C-4), 53.3 (C-7), 40.0 (C-6), 31.5 (CH_3_-N9), 24.2 (CH_3_-N1); HRMS (ESI+): Calcd for C_13_H_15_BrN_4_O_3_Na [M + Na]^+^ 377.02197, found: 377.02225. 

#### 3.5.2. (5aS,5bS,8aS,9aR)-1-Bromo-8a-hydroxy-6,8-dimethyl-5,5a,5b,6,8,8a,9,9a-octahydroimidazo [4’,5’:4,5]cyclopenta [1,2-e]pyrrolo [1,2-a]pyrazine-4,7-dione (**1m**)

Data for **1m**: ^1^H NMR (400 MHz, CD_3_OD) δ 6.91 (d, J = 4.1, H-15), 6.32 (d, J = 4.1, H-14), 4.55 (m, H-7), 4.28 (d, J = 5.7, H-8), 3.79 (s, H-4), 2.91 (s, CH_3_-3), 2.82 (s, CH_3_-1), 2.66 (dd, J = 13.0, 6.4, Ha-6), 2.11 (dd, J = 13.0, 12.2, Hb-6); ^13^C NMR (100 MHz, CD_3_OD) δ 159.7 (C-10), 158.8 (C-2), 122.7 (C-11), 114.7 (C-15), 112.4 (C-14), 106.0 (C-13), 91.8 (C-5), 71.9 (C-4), 57.2 (C-8), 52.9 (C-7), 38.8 (C-6) 28.6 (CH_3_-N3), 23.4 (CH_3_-N1); HRMS (ESI+): Calcd for C_13_H_15_BrN_4_O_3_Na [M + Na]^+^ 377,02197, found: 377.02252. 

#### 3.5.3. (5aS,5bS,8aS,9aR)-1-Bromo-8a-methoxy-5,8-dimethyl-5,5a,5b,6,8,8a,9,9a-octahydroimidazo [4’,5’:4,5]cyclopenta [1,2-e]pyrrolo [1,2-a]pyrazine-4,7-dione (**2d**)

Preparation of **2d**. A small sample of **1d** was dissolved in MeOH and stirred at r.t. for 48 h in the presence of A15 to give the corresponding **2d**.

Data for **2d**: ^1^H NMR (300 MHz, CDCl_3_) δ 6.95 (d, J = 4.1, H-15), 6.28 (d, J = 4.1, H-14), 4.64 (m, H-7), 4.24 (br.s, H-4), 3.93 (br.d, J = 5.5, H-8), 3.18 (CH_3_O-5), 3.14 (s, CH_3_-9), 2.82 (s, CH_3_-1), 2.60 (dd, J = 13.0, 6.5, Ha-6), 2.25 (dd, J = 13.0, 12.0, Hb-6); (75 MHz, CDCl_3_) δ 159.5 (C-10), 158.1 (C-2), 123.2 (C-11), 115.3 (C-15), 113.2 (C-14), 104.6 (C-13), 97.7 (C-5), 67.5 (C-8), 58.5 (C-4), 52.2 (C-7), 50.4 (CH_3_O-5), 39.1 (C-6), 31.2 (CH_3_-N9), 24.4 (CH_3_-N1). 

#### 3.5.4. (5aS,5bS,8aS,9aR)-1-Bromo-8a-methoxy-6,8-dimethyl-5,5a,5b,6,8,8a,9,9a-octahydroimidazo [4’,5’:4,5]cyclopenta [1,2-e]pyrrolo [1,2-a]pyrazine-4,7-dione (**2m**)

Preparation of **2m**: A small sample of **1m** was dissolved in MeOH and stirred at r.t. for 48 h in the presence of A15 to give the corresponding **2m**.

Data for **2m**: ^1^H NMR (400 MHz, CD_3_OD) δ 6.92 (d, J = 4.1, H-15), 6.33 (d, J = 4.1, H-14), 4.59 (m, H-7), 4.31 (d, J = 5.6, H-8), 3.94 (s, H-4), 3.12 (CH_3_O-5), 2.91 (s, CH_3_-3), 2.80 (s, CH_3_-1), 2.68 (dd, J = 13.2, 6.5, Ha-6), 2.16 (dd, J = 13.2, 12.1, Hb-6). 

The general procedure applied to reactions followed by NMR spectroscopy and identification of **3m**, **3d**, and **5d**: A sample (~2–4 mg) of the appropriate substrate (**2e**, **1m** or **1d**) was poured into an NMR tube, dried and dissolved in CDCl_3_. Then, MsOH was added in portion until the signals of alkaloid methyls and acid methyl integrated roughly equal. The solution was warmed at 60–65 °C for several hours, and proton spectra were acquired every hour. Finally, the mixture was neutralised by adding a few beads of A21. NMR signals showed a downfield shift when in an acidic solution but moved to the usual shift after neutralisation. Each experiment was repeated 2–3 times to confirm the observed results.

#### 3.5.5. (5aR,9aR)-1-Bromo-6,8-dimethyl-5,5a,6,8,9,9a-hexahydroimidazo [4’,5’:4,5]cyclopenta [1,2-e]pyrrolo [1,2-a]pyrazine-4,7-dione (**3m**)

Data for **3m**: ^1^H NMR (400 MHz, CDCl_3_) δ 7.18 (d, J = 4.3, H-15), 6.51 (d, J = 4.3, H-14), 5.37 (m, H-7), 5.29 (br.d, J = 6.9, H-8), 3.51 (dd, J = 14.3, 7.2 Ha-6), 3.50 (s, CH_3_-9), 3.34 (s, CH_3_-1), 2.74 (ddd, J = 14.3, 6.9, 1.5, Hb-6). (NB! Proton resonances may show a low field shift caused by MsOH).

#### 3.5.6. (5aR,9aR)-1-Bromo-5,8-dimethyl-5,5a,6,8,9,9a-hexahydroimidazo [4’,5’:4,5]cyclopenta [1,2-e]pyrrolo [1,2-a]pyrazine-4,7-dione (**3d**)

Data for **3d**: ^1^H NMR (400 MHz, CDCl_3_) δ 6.99 (d, J = 4.0, H-15), 6.36 (d, J = 4.0, H-14), 5.34 (m, H-7), 5.07 (dd, J = 7.0, 1.4, H-8), 3.49 (dd, J = 14.7, 7.4 Ha-6), 3.44 (s, CH_3_-9), 3.08 (s, CH_3_-1), 2.77 (ddd, J = 14.7, 6.9, 1.4, Hb-6). (NB! Proton resonances may show a low field shift caused by MsOH).

#### 3.5.7. 4-[(2,3-Dihydro-3-methyl-2-oxo-1H-imidazol-4-yl)methyl]-2-methylpyrrolo [1,2-a]pyrazin-1(2H)-one (**5d**)

Data for **5d**: ^1^H NMR (400 MHz, CDCl_3_) δ 7.20 (dd, J = 4.0, 1.5, H-15), 7.11 (dd, J = 2.7, 1.5, H-13), 6.63 (dd, J = 4.0, 2.7, H-14), 6.13 (dt, J = 2.3, 1.1, H-4), 6.09 (t, J = 1.1, H-8), 3.74 (dd, J = 1.1, 1.1, 2H-6), 3.43 (s, CH_3_-9), 3.22 (s, CH_3_-1). HRMS (ESI+): Calcd for C_13_H_14_N_4_O_2_Na [M + Na]^+^ 281.10090, found: 281.10108.

## 4. Conclusions

The present results suggest that a locally acidic environment at the site of agelastatin A accommodation can induce two processes: (1) the release of an oxidant bromonium ion and (2) the structural modification of **1** through C4–C8 bond cleavage. Notably, the second hypothesis does not conflict with previous SAR studies [2,13,14,15,16,17,18,19]. In agelastatin D, the C8 (**B**) and the C5 (**A**) carbocations are equally stable; in **1,** the ion **A** is better stabilised than the **B** ion. The C4–C8 bond breaks more easily if the C8 carbocation (**B**) is more stable than the C5 carbocation (**A**). For the C4–C8 bond to break, some electron donor to N9 must be present at the agelastatin A accommodation site. A methyl bonded to the N9 nitrogen makes it possible to break the bond before **1** reaches the binding pocket; thus, the alkylation of N9 leads to a complete loss of activity. The positive charge on C8 can also be stabilised by weakening the resonance structure of the N9–C10 amide bond. That is, the C10 carbonyl is predominantly conjugated to pyrrole. In that case, the C10–N9 amide bond would assume the stereochemistry of a twisted amide, and the N9 lone pair would be fully available to compensate for the positive charge on C8: it will actually be an iminium ion instead of acyliminium. Computational calculations could refute or confirm this resonance effect, particularly when an electron-withdrawing substituent is positioned at C13. The structural modification of **1** could be responsible for the irreversible conformational changes of the host enzyme [20]. To date, several synthetic routes have been established to obtain the total synthesis of agelastatin A and its derivatives; none chose C2 as a suitable modification site to prepare analogues with enhanced biological activity. 

The chemical structure of agelastatin A is revealed as a precarious scaffolding that stands on the delicate balance of electronic distribution. Let us imagine that agelastatin A behaves like a mine. Its molecular attachment to the host protein binding site could trigger a cascade of reactions that will ultimately prove fatal to the cell. Its mode of action closely resembles that of calicheamicin γ_1_^I^ [28].

In the author’s experience, sarcodictyins [29] and parthenolide [30,31] also behave like agelastatin A: (i) on hydrolysis of urocanate moiety, sarcodictyin C breaks down and releases 2-Penten-4-olide; (ii) on the opening of the epoxide ring, parthenolide undergoes an intramolecular electrophilic cyclisation. This indicates that molecular recognition is only the first process that the medicine induces to exert its pharmacological action. More attention should probably be paid to the intrinsic reactivity of biologically active compounds. Several papers review medicines according to their function; as far as I know, it is difficult to find a classification based on chemical properties [32,33].

## Figures and Tables

**Table 1:** Comparison of four reactions according to chromatographic and spectroscopic results. Full comments are given in the Supplementary (Figure S6a).

Reaction	Conditions	Compounds by ^1^H-NMR	Compound, t_R_ and Possible [M + H]^+^ (*m*/*z*) by LC-DAD-MS Experiments. Style by UV Bands.			
-	Mix of known compounds	**6e 1 1d 1m 1e 2m 2e**	**6e**/14.5 **1**/15.9 **1d**/17.6 **1m**/19.1 **1e**/21.3 **2m**/25.5 **2e**/29.2			
R6	**2e** + 0.1eq.MsOH, CHCl_3_, 70°, 24 h	**4e** (2%) **3e** (90%) **2e** (10%)	**2’e**/19.9	**4e**/24.2	***3e***/*25.9*	**2e**/28.6
R7	**2e** + 0.5eq.MsOH, CHCl_3_, 70°, 24 h	**3e**~**1e** 40% **4e** 10% **6e** 8% **5e** (2%)	**1’e**/13.6 (369–371) **1e**/20.3 (369–371)	**6e**/14.1 (273) **4e**/23.9 (351–353)	**5e**/14.4 (273) ***3e***/*25.7 (351–353)*	**2’e**/19.5 (383–385) **2e**/28.4 (383–385)
R8	**2e** + 1.1eq.MsOD, CDCl_3_, 70°, 1.5 h	**6e** (70%), **5e** (30%)		**6e**/14.1 (273)	**5e**/14.3 (273)	
R9	**2e** + 0.8eq.MsOH, CHCl_3_, 70°, 1.2 h	(First aliquot after 15’)		**6e**/14.1 **4e**/23.9	**5e**/14.3 ***3e***/*25.7*	**2e**/28.3
		**6e** (95%), **5e** (5%)		**6e**/14.1 (273)	**5e**/14.3 (273)	

## Data Availability

Data of the compounds are available in Supplementary Materials.

## Supplementary Materials

The following supporting information can be downloaded at https://www.mdpi.com/article/10.3390/molecules28196821/s1.

## Sample Availability

Only a few samples of the compounds are available from the authors upon request.
