# Supplementary material for: The N-Alkylation of Agelastatin A Modulates Its Chemical Reactivity
Source: Molecules. 2023 Sep 27;28(19):6821. doi: 10.3390/molecules28196821 (PMC10574546; doi:10.3390/molecules28196821)
Supplement: Supplementary file 1 [file molecules-28-06821-s001.zip › molecules-2611392-supplementary.pdf]

The *N*-Alkylation of Agelastatin A Modulates Its Chemical Reactivity

Michele D'Ambrosio

Laboratory of Bioorganic Chemistry, Department of Physics

Università degli Studi di Trento

Via Sommarive 14, 38123 Trento, Italy

Correspondence should be addressed to: michele.dambrosio@unitn.it

Figure S1. Proposed arbitrary numbering:

Arbitrary numbering is required to designate the many derivatives of agelastatin A. This arbitrary numbering meets the following criteria: **1)** never use a letter that also represents an element symbol; **2)** a number identifies the multiplicity of the C4-C5 bond, the absolute configuration (4S, 5S) and the substituent group at the C5 oxygen; **3)** a possible apostrophe indicates the opposite configuration (4R, 5R) ; **4)** a letter establishes the number and position of the methyl groups at the nitrogen N1, N3 and N9. Three tables exemplify the above rules:

C4 - C5 bond multiplicity	Single				Double
C4 and C5 configurations	*4S, 5S*		*4R, 5R*		-
C5-O- substituent	H	Me	H	Me	-
Adopted numbering	**1**	**2**	**1'**	**2'**	**3**

**1a**	**1**	**1j**	**1t**	**1m**	**1d**	**1z**	**1e**
**1'a**	**1'**	**1'j**	**1't**	**1'm**	**1'd**	**1'z**	**1'e**
**2a**	**2**	**2j**	**2t**	**2m**	**2d**	**2z**	**2e**
**2'a**	**2'**	**2'j**	**2't**	**2'm**	**2'd**	**2'z**	**2'e**
**3a**	**3**	**3j**	**3t**	**3m**	**3d**	**3z**	**3e**

Number of substituents	Zero	One			Two			Three
Substituted nitrogen(s)	-	1	3	9	1,3	1,9	3,9	1,3,9
Designating letter	**a**	**-**	**j**	**t**	**m**	**d**	**z**	**e**

Forty possible derivatives of agelastatin A. Compounds not yet prepared are in blue

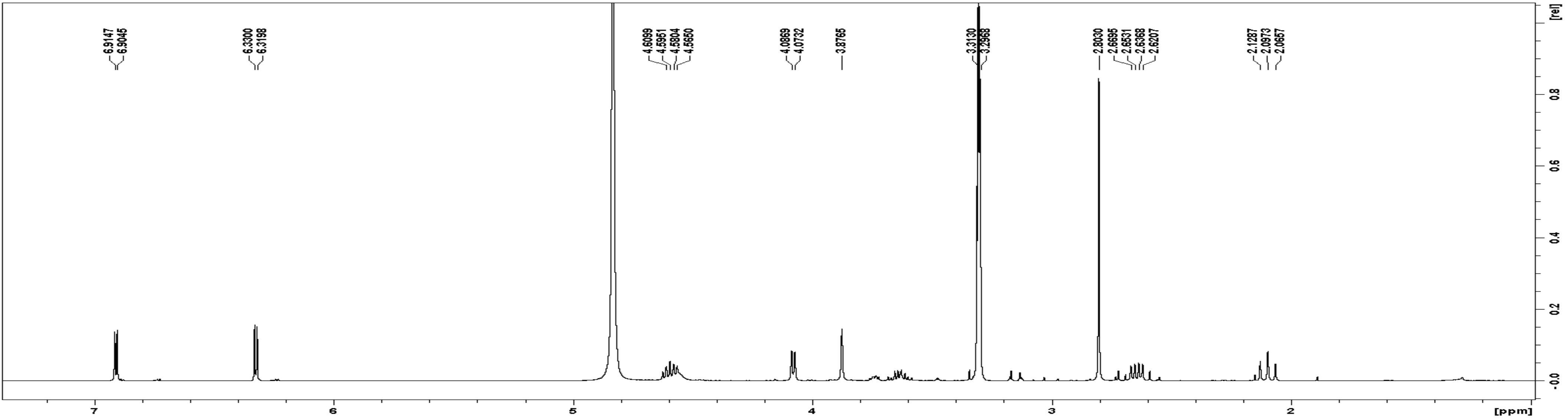

Figure S1a. - ↑ – CD_3OD **1** ↓ – $CDCl_3$

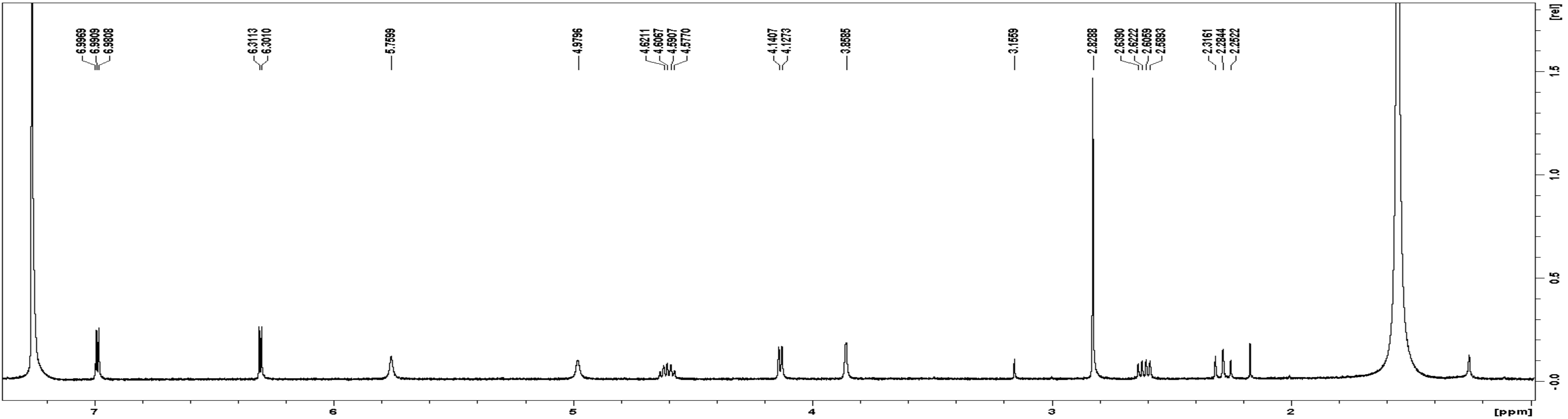

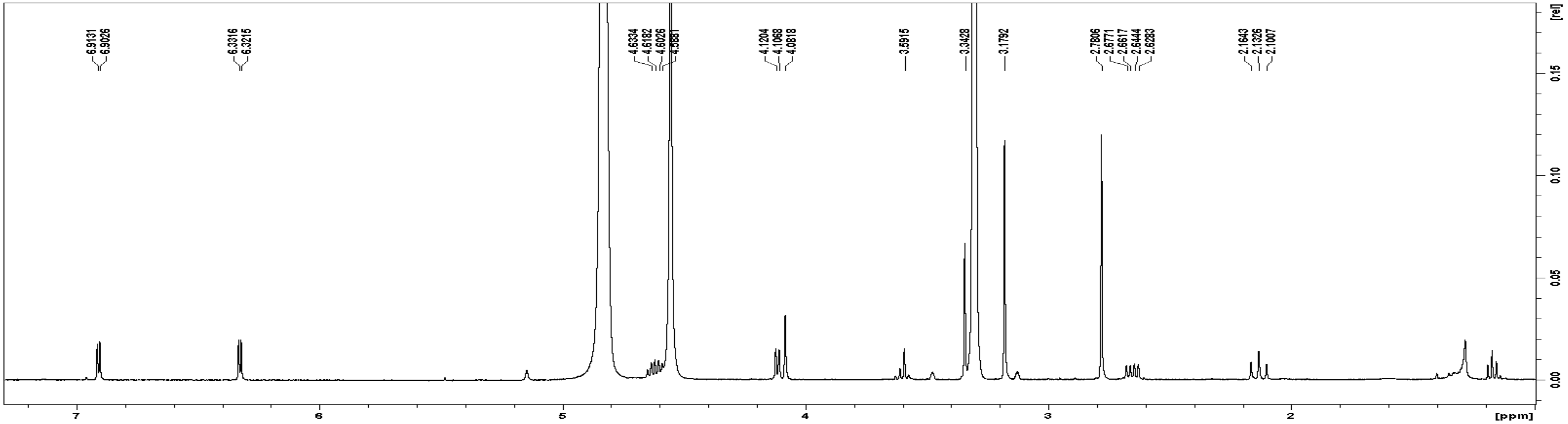

Figure S1b. - ↑ – CD_3OD **2** ↓ – $CDCl_3$

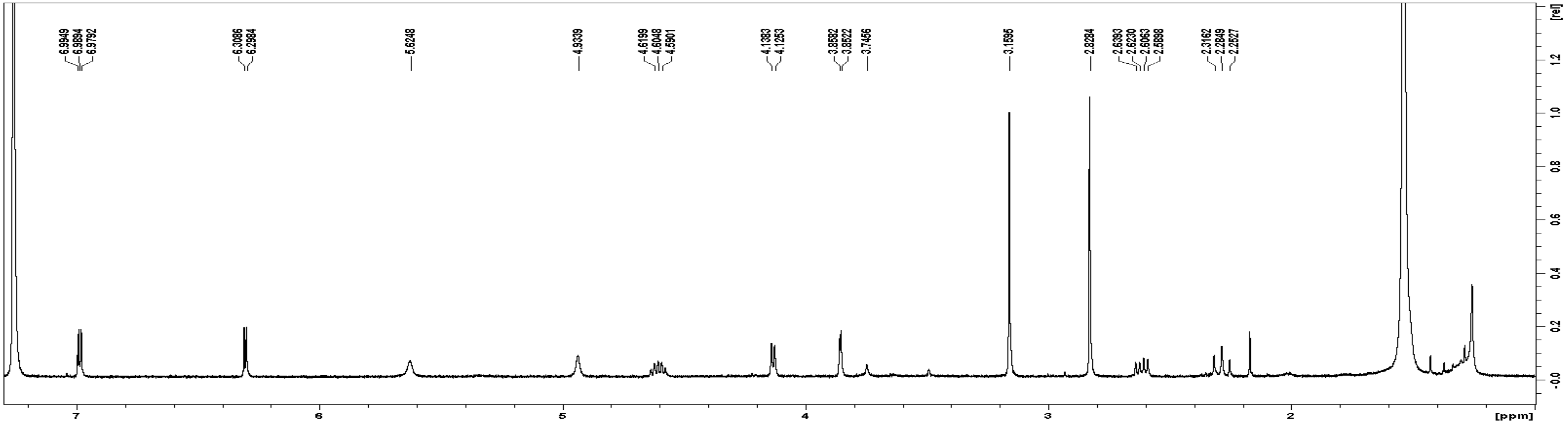

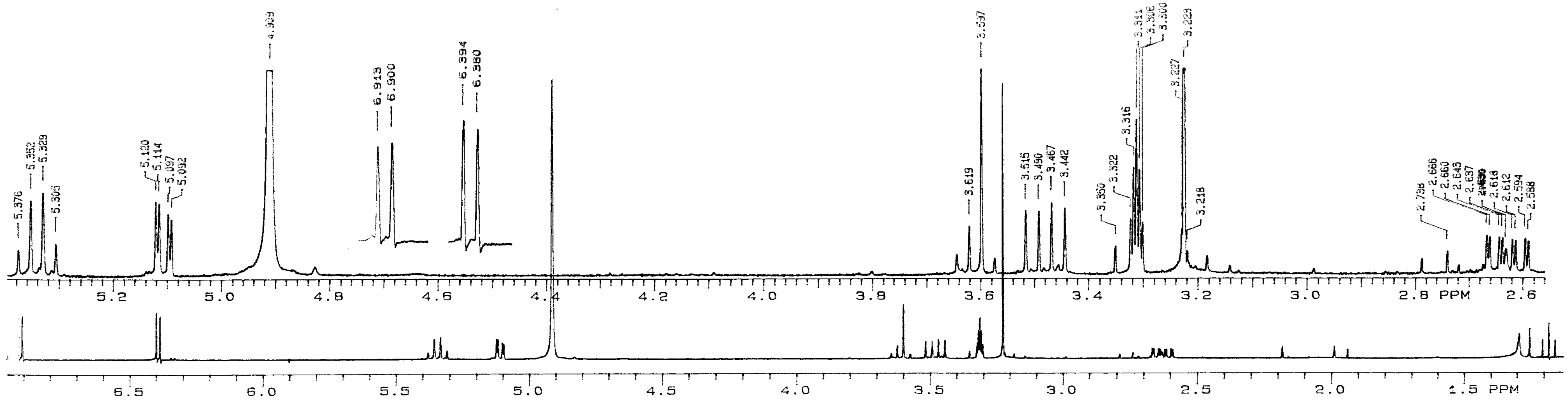

Figure S1c. - ↑– **3** – CD_3OD ↓ – **3e** $CDCl_3$

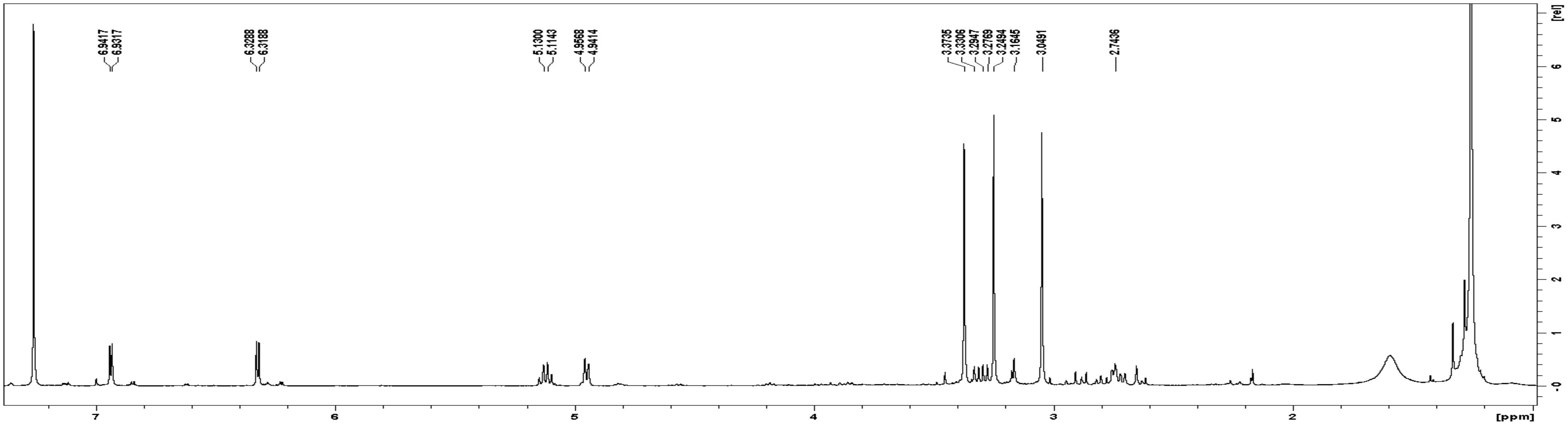

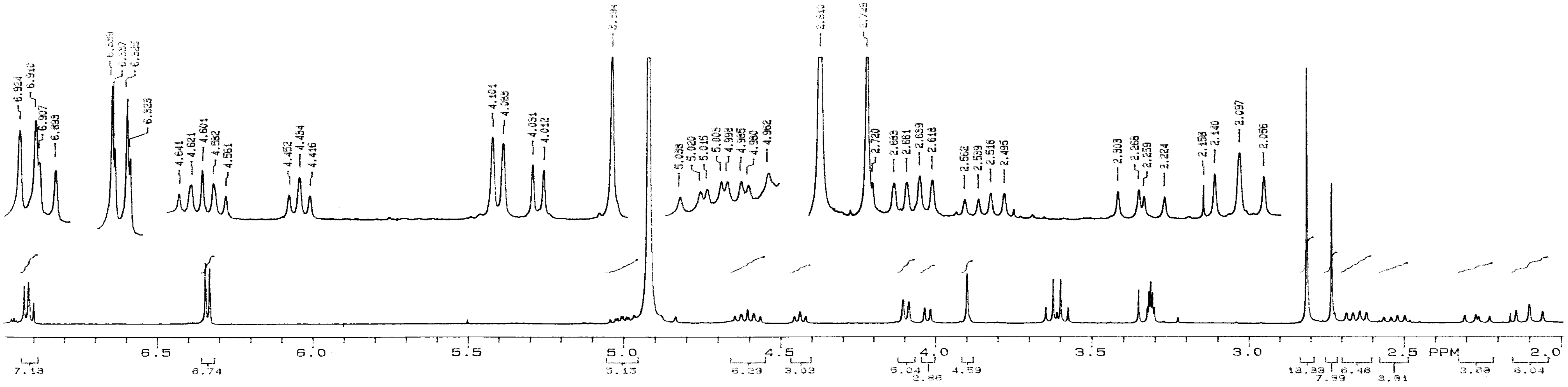

Figure S1d. - ↑ – **1+1'** – CD_3OD ↓ – **2+2'** – CD_3OD

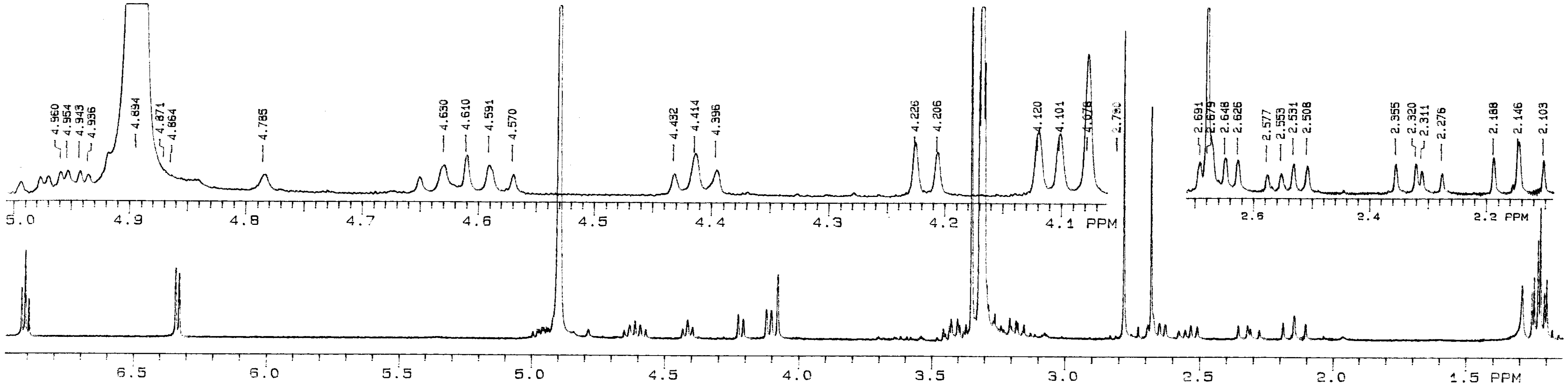

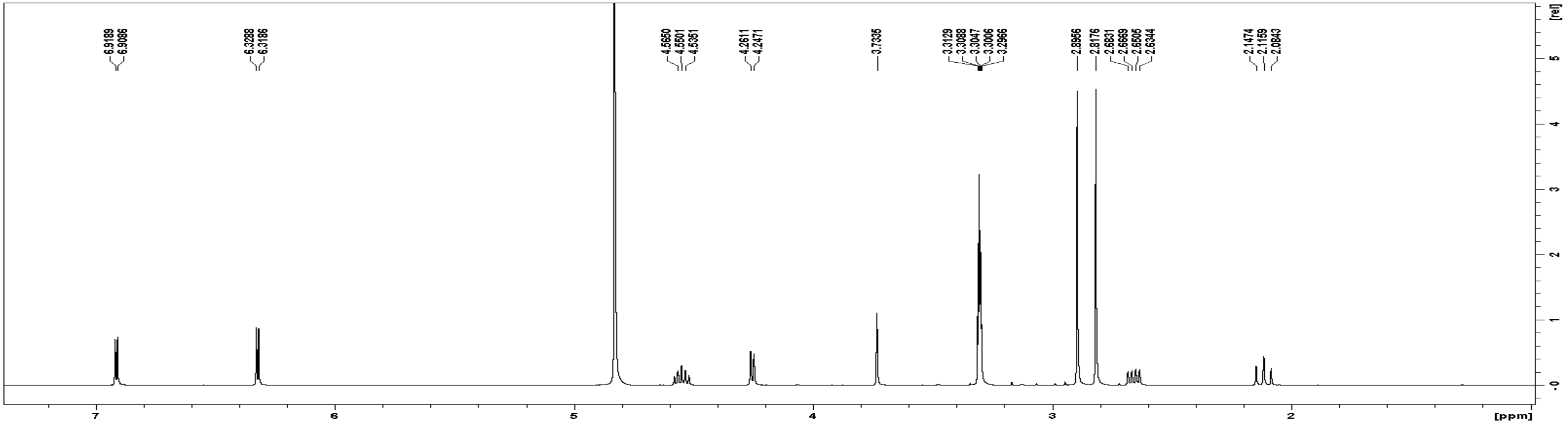

Figure S1e. - ↑ – 1m ↓ – 2m > 2e – CD_3OD

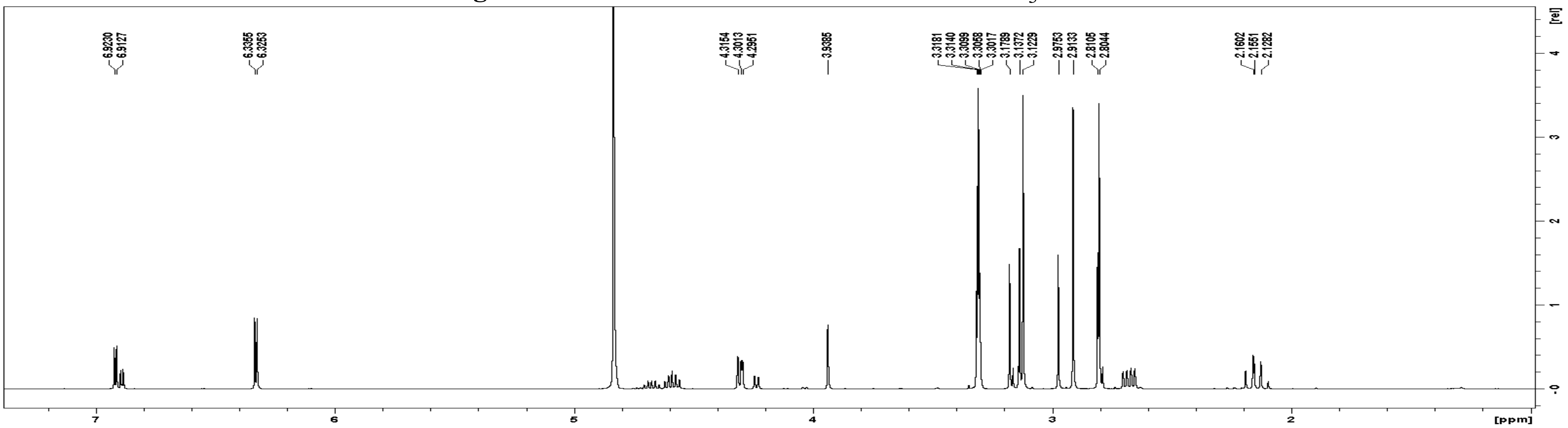

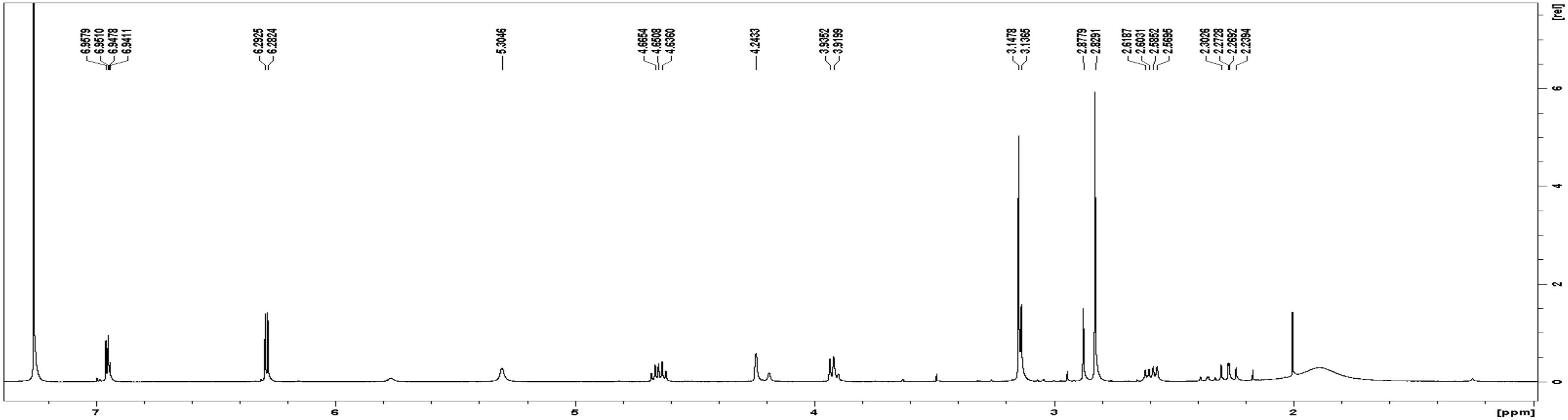

Figure S1f. - ↑ – 1d ↓ – 2d

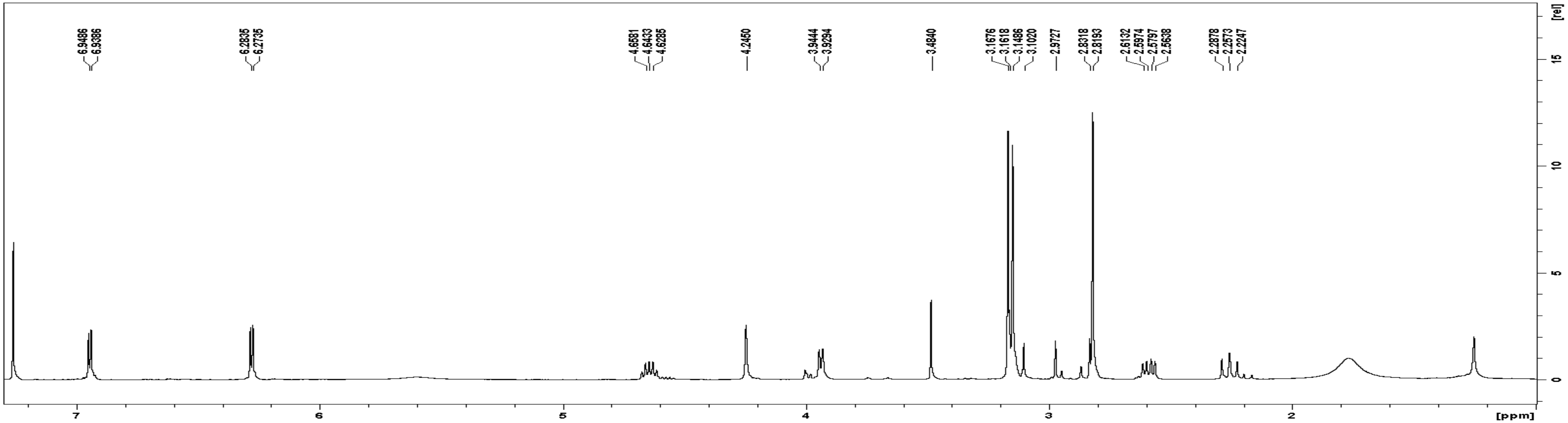

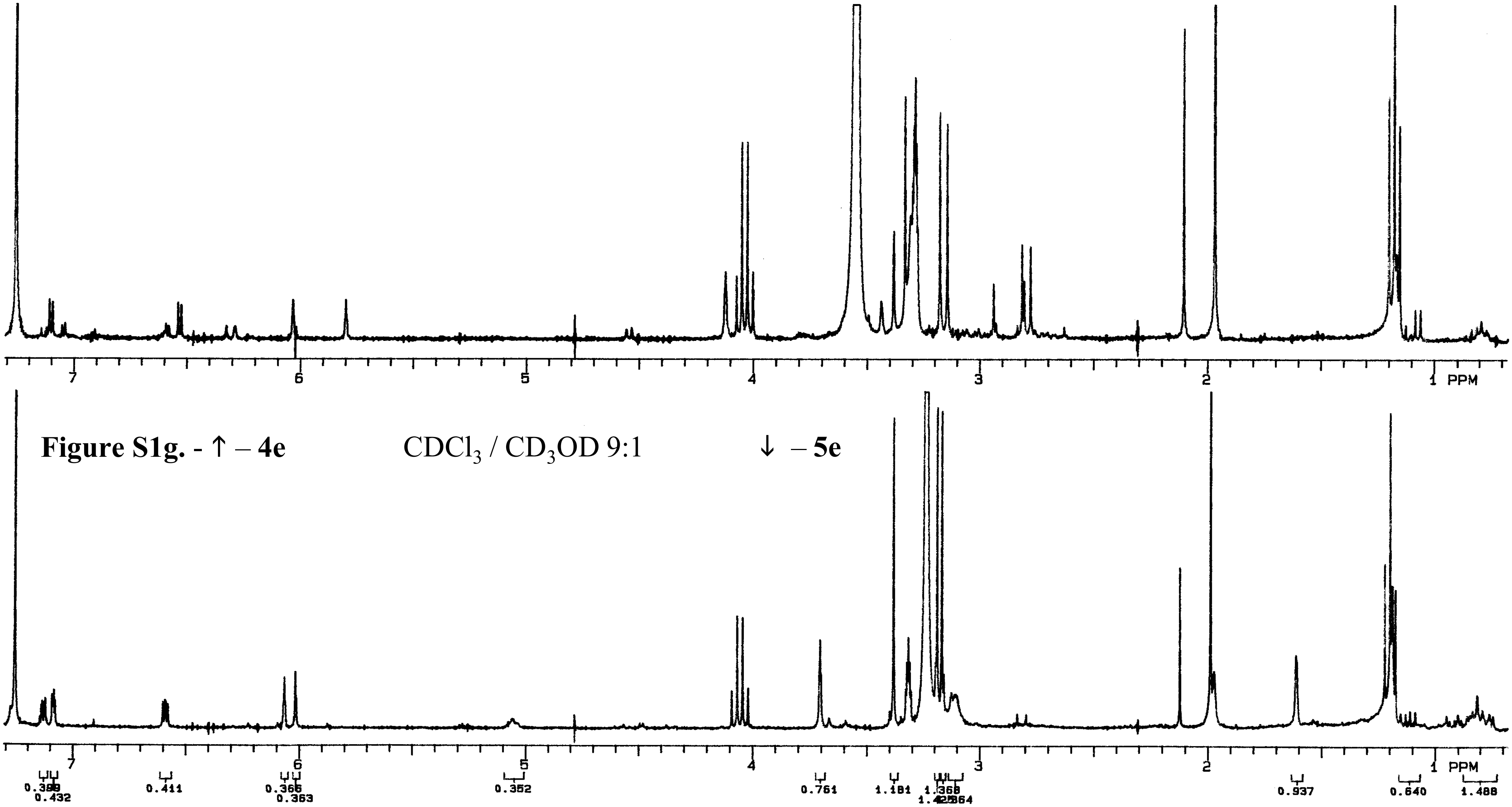

Figure S1g. - ↑ – 4e $CDCl_3$ / CD_3OD 9:1 ↓ – **5e**

Figure S1h. – 6e

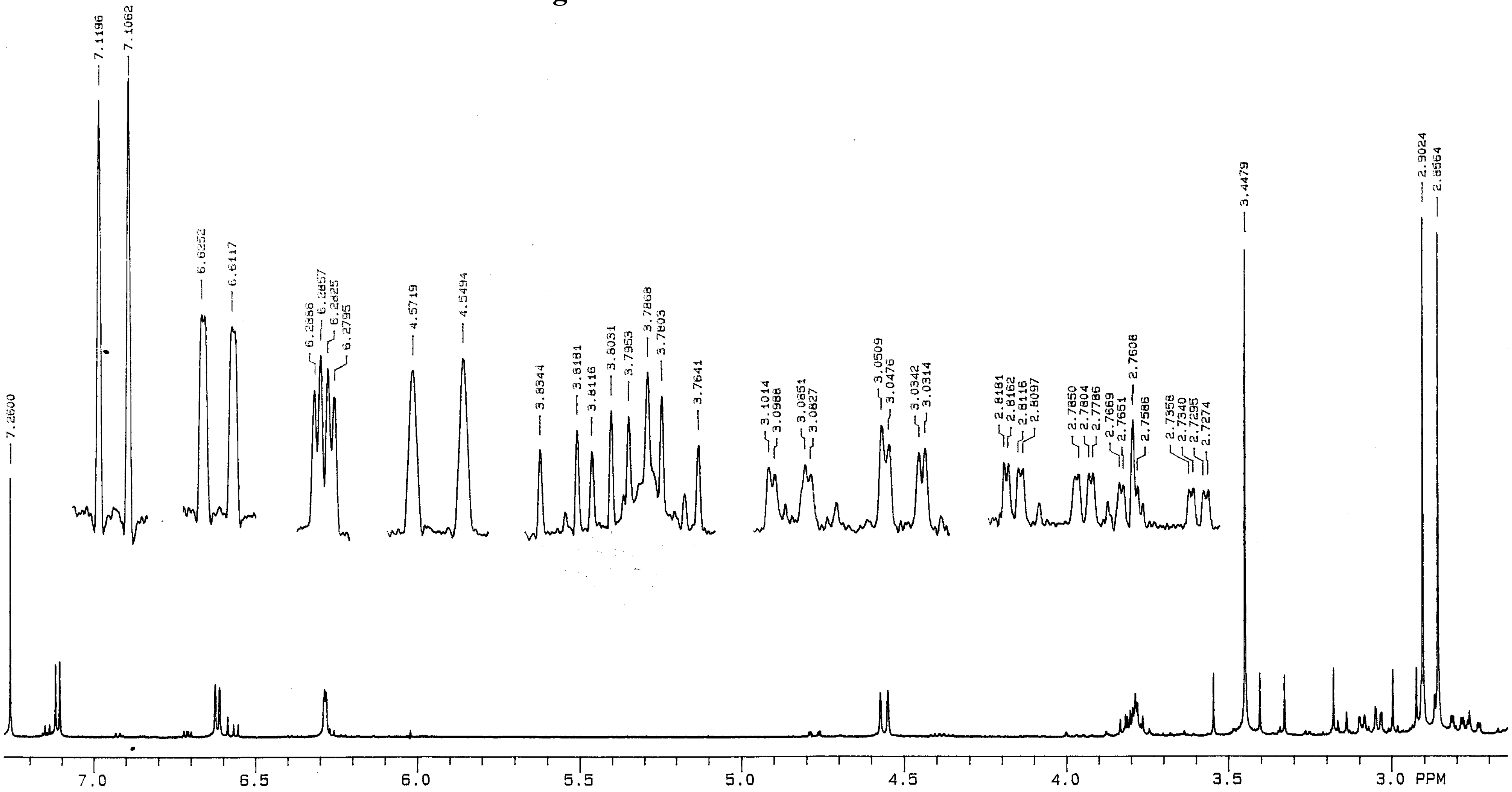

Figure S1i. – 13Cdept – **6e**

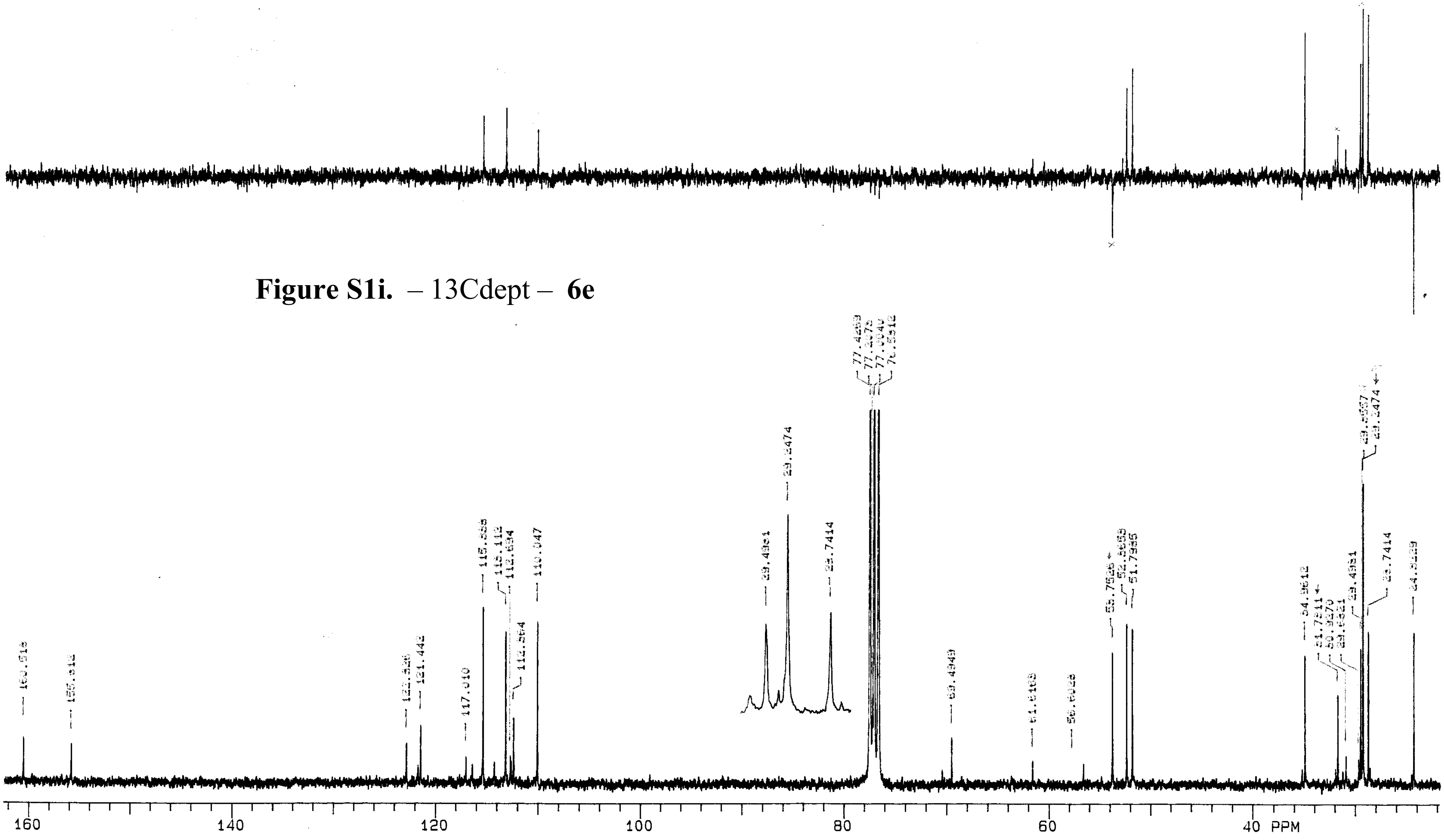

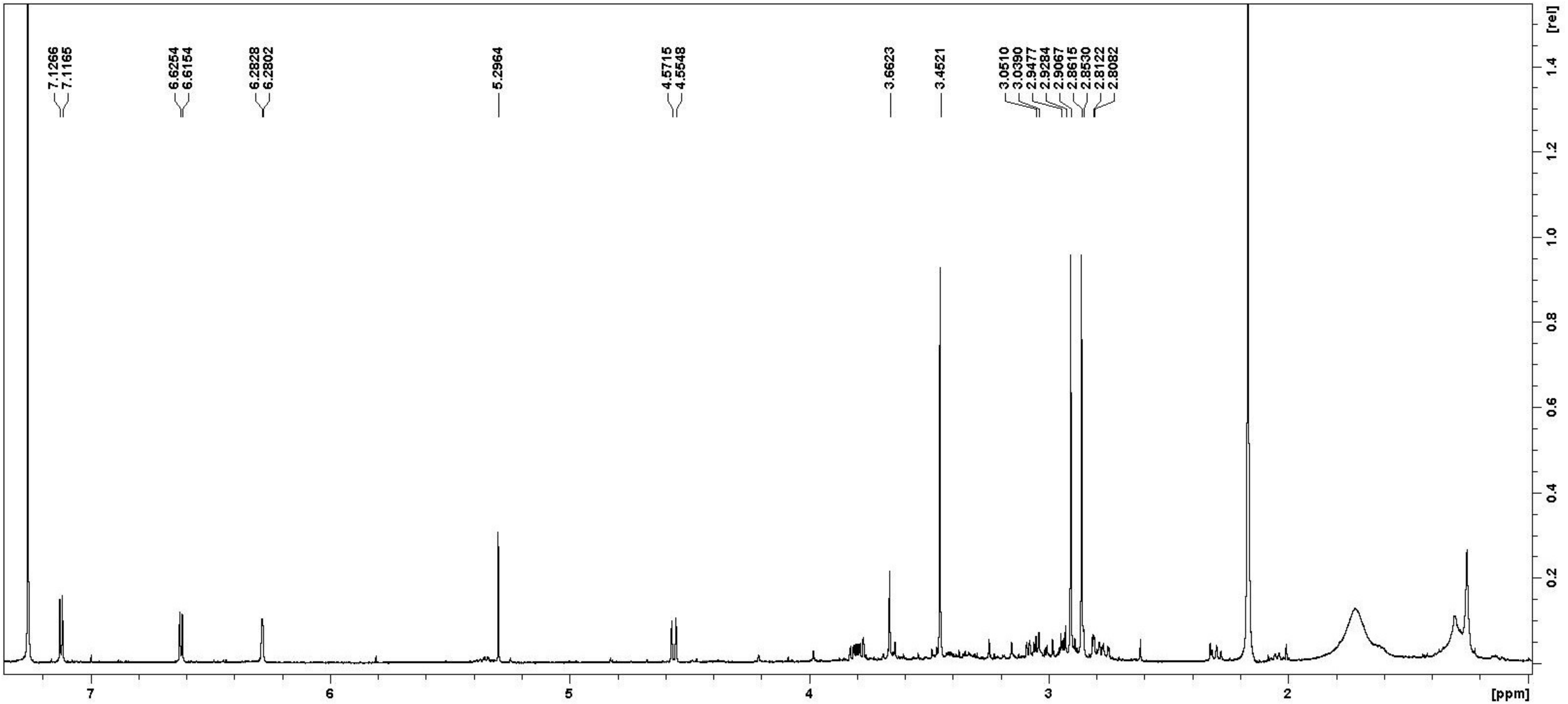

Figure S2a. – **2e** + 2 equiv. MsOH, reflux – solvent $CHCl_3$

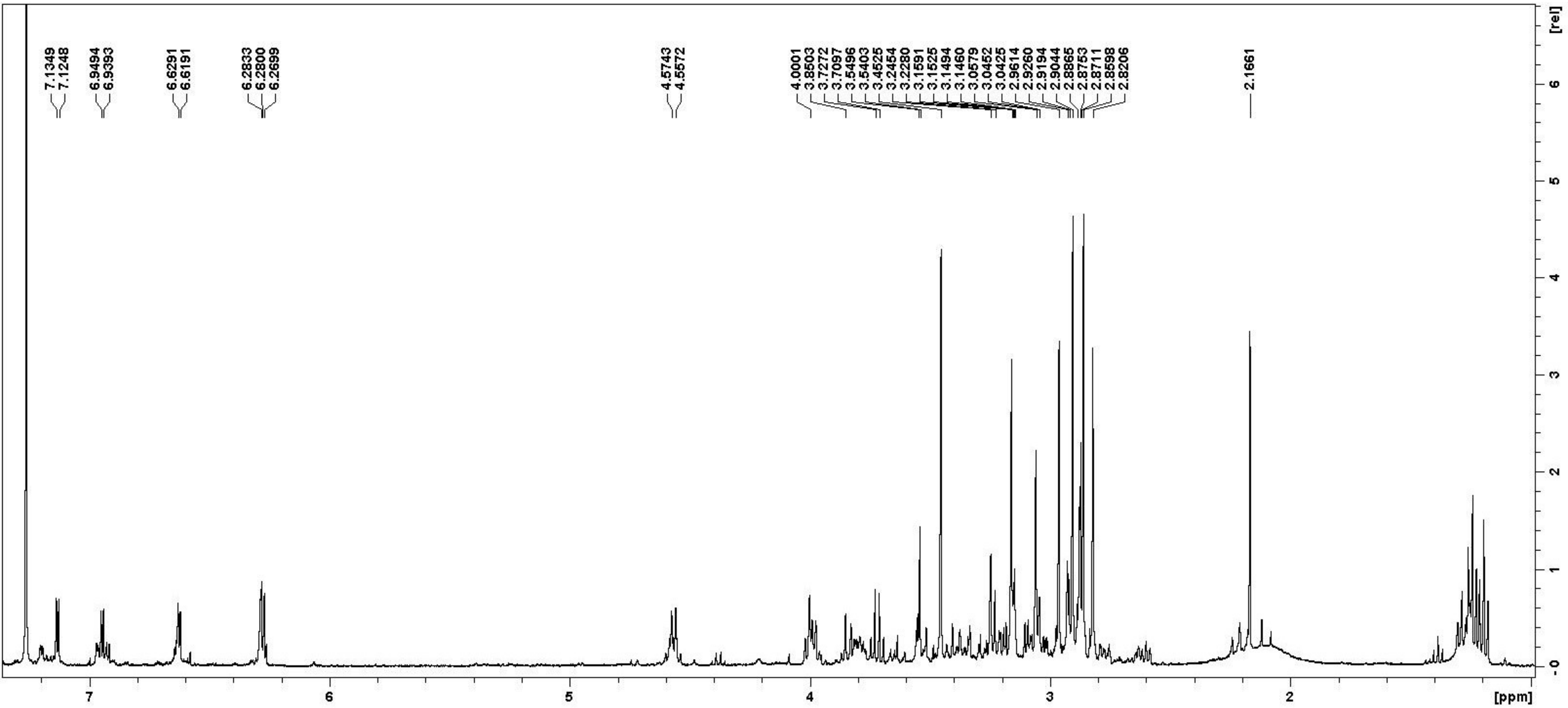

Figure S2b. – **2e** + 2 equiv. MsOH, reflux – solvent CH_2Cl_2

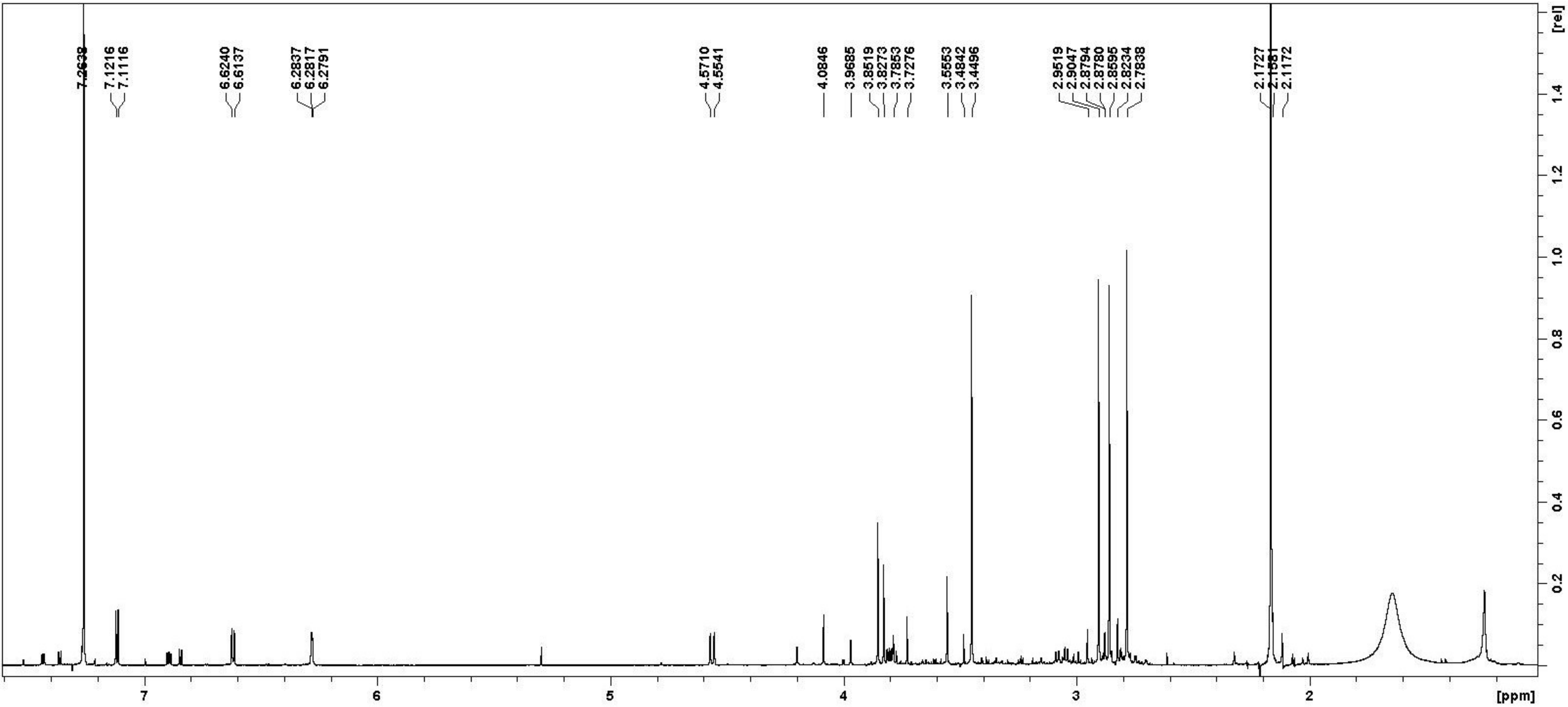

Figure S2c. – **2e** + 2 equiv. MsOH, reflux – solvent CH_2ClCH_2Cl

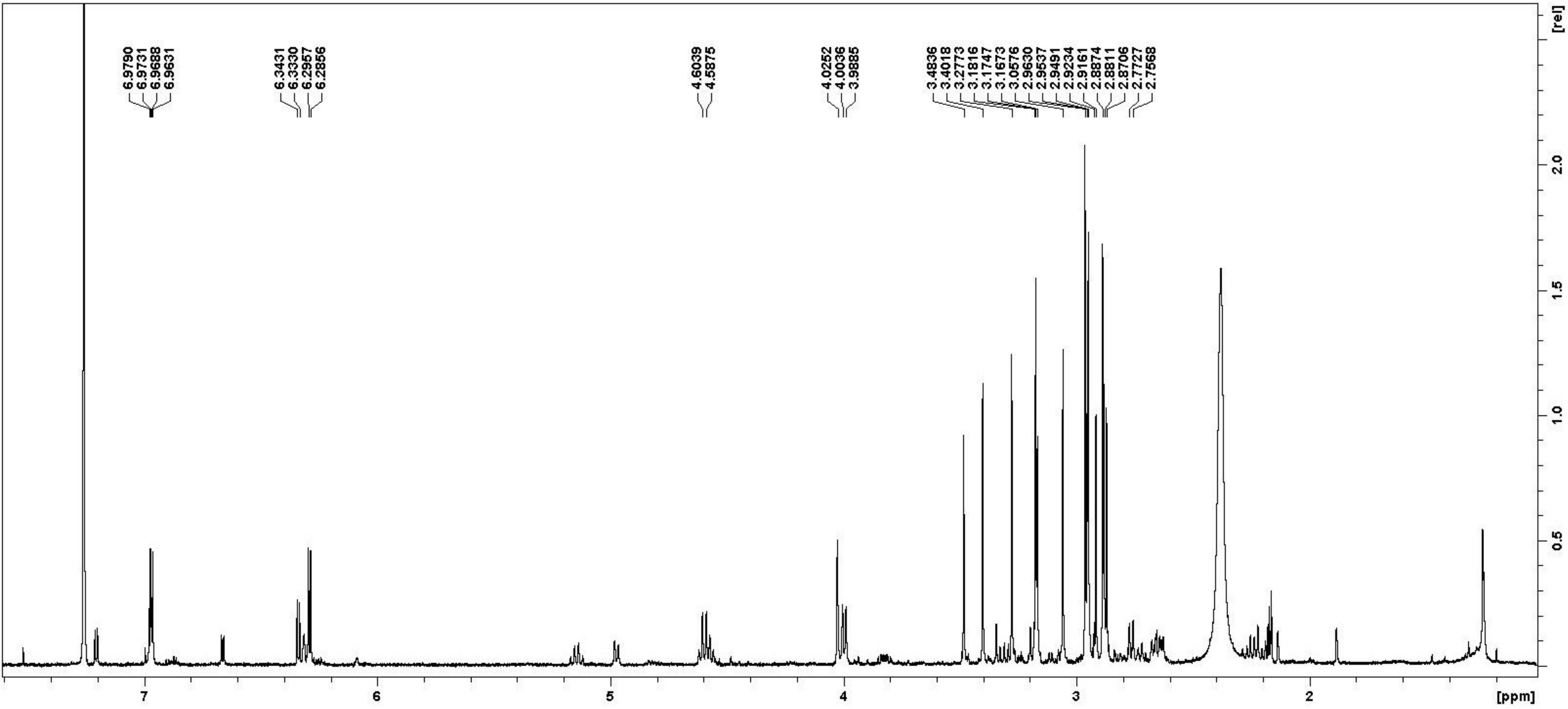

Figure S2d. – **2e** + 2 equiv. MsOH, reflux – solvent $(CH_3)_2C{=}O$

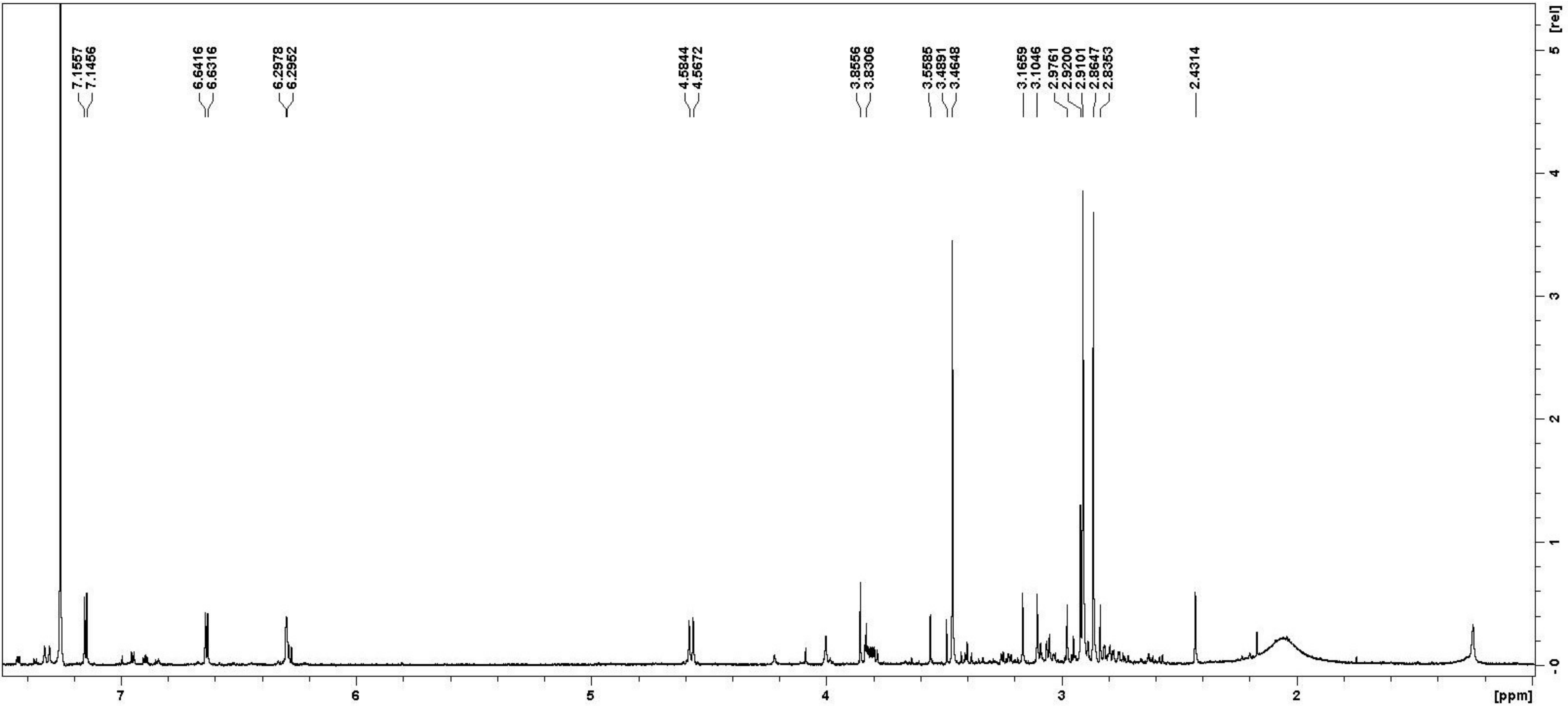

Figure S2e. – **2e** + 2 equiv. MsOH, $CHCl_3$ - room temperature, 6 days

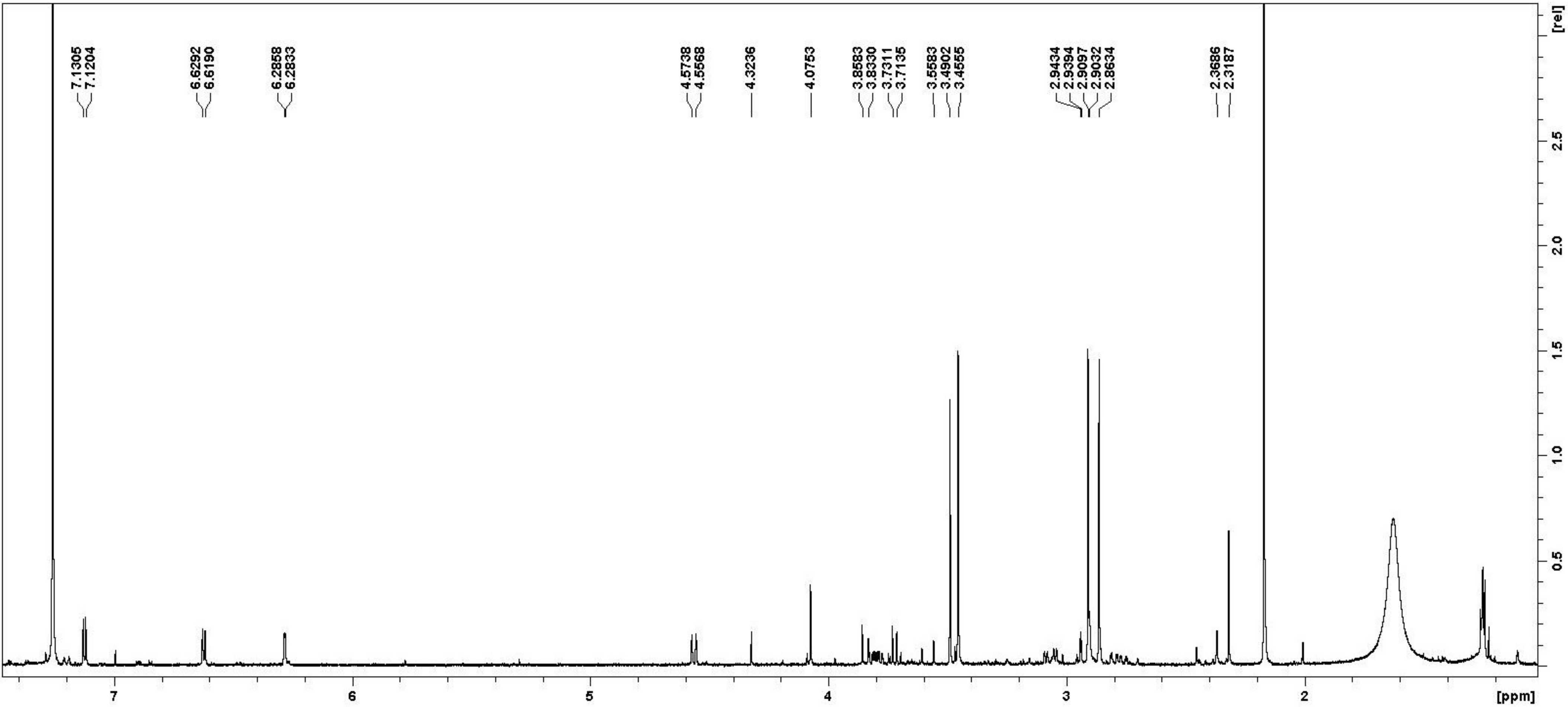

Figure S3a. – **2e** in $CHCl_3$ reflux, 2 equiv. pTsOH

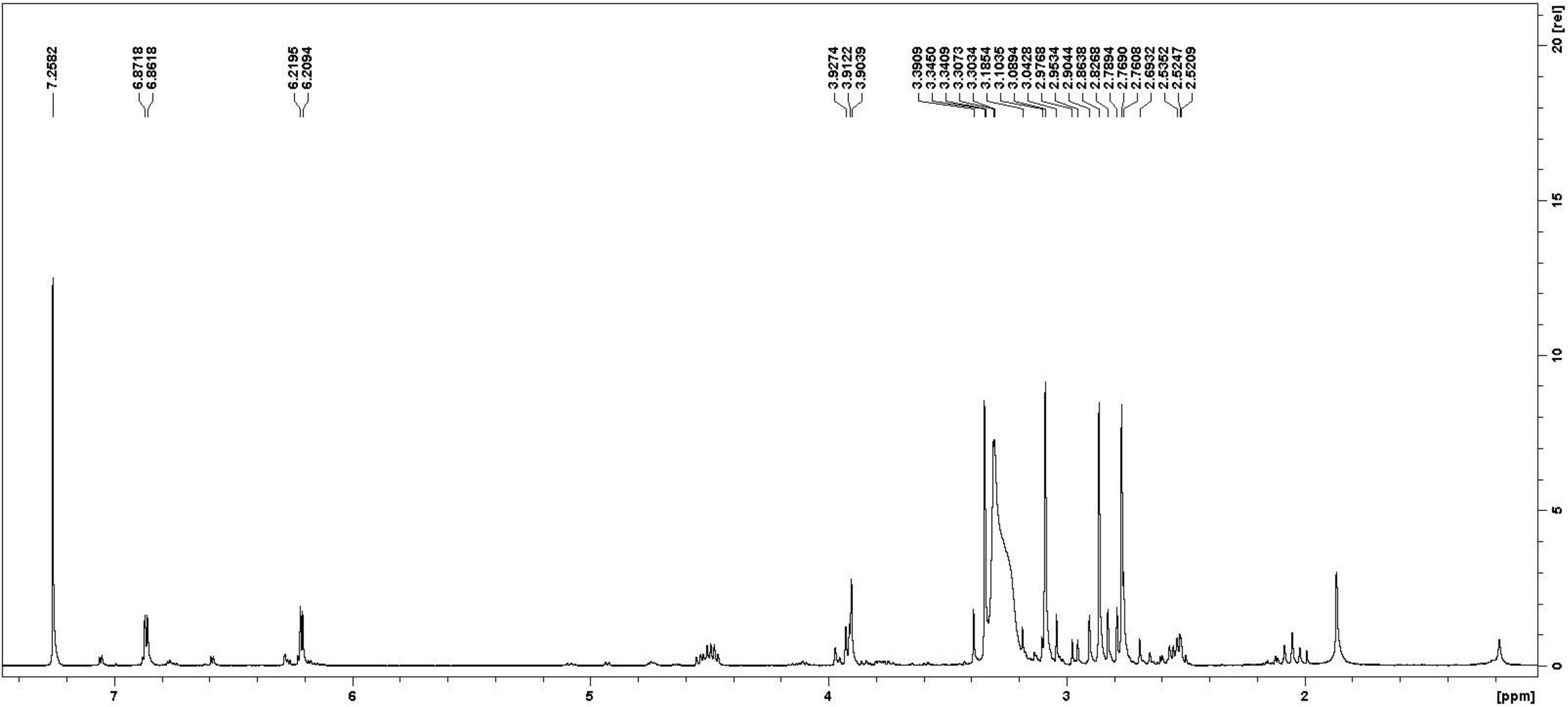

Figure S3b. – **2e** in $CHCl_3$ reflux, 2 equiv. AcCl

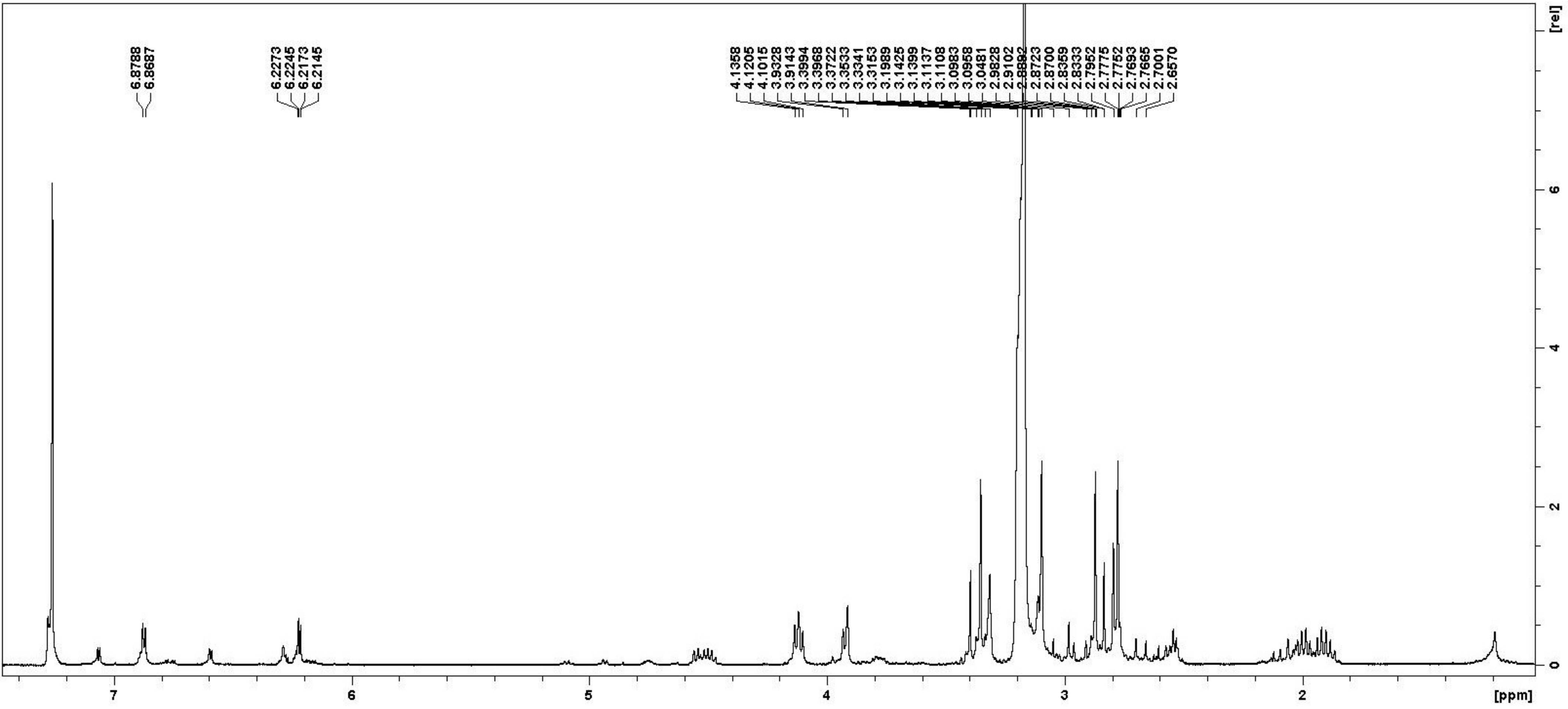

Figure S3c. – **2e** in $CHCl_3$ reflux, 2 equiv. PrCOBr

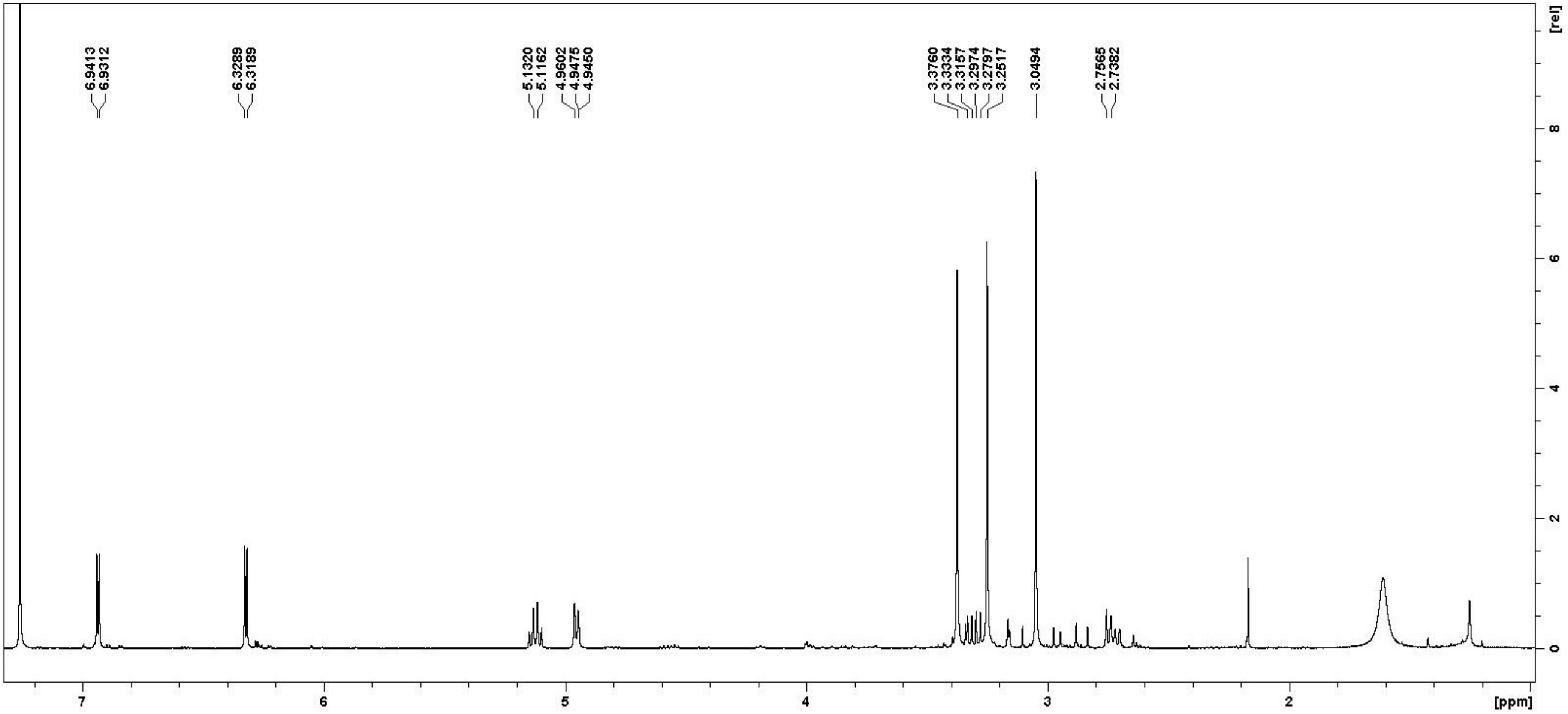

Figure S3d. – **2e** in $CHCl_3$ reflux, 2 equiv. CF3COOH

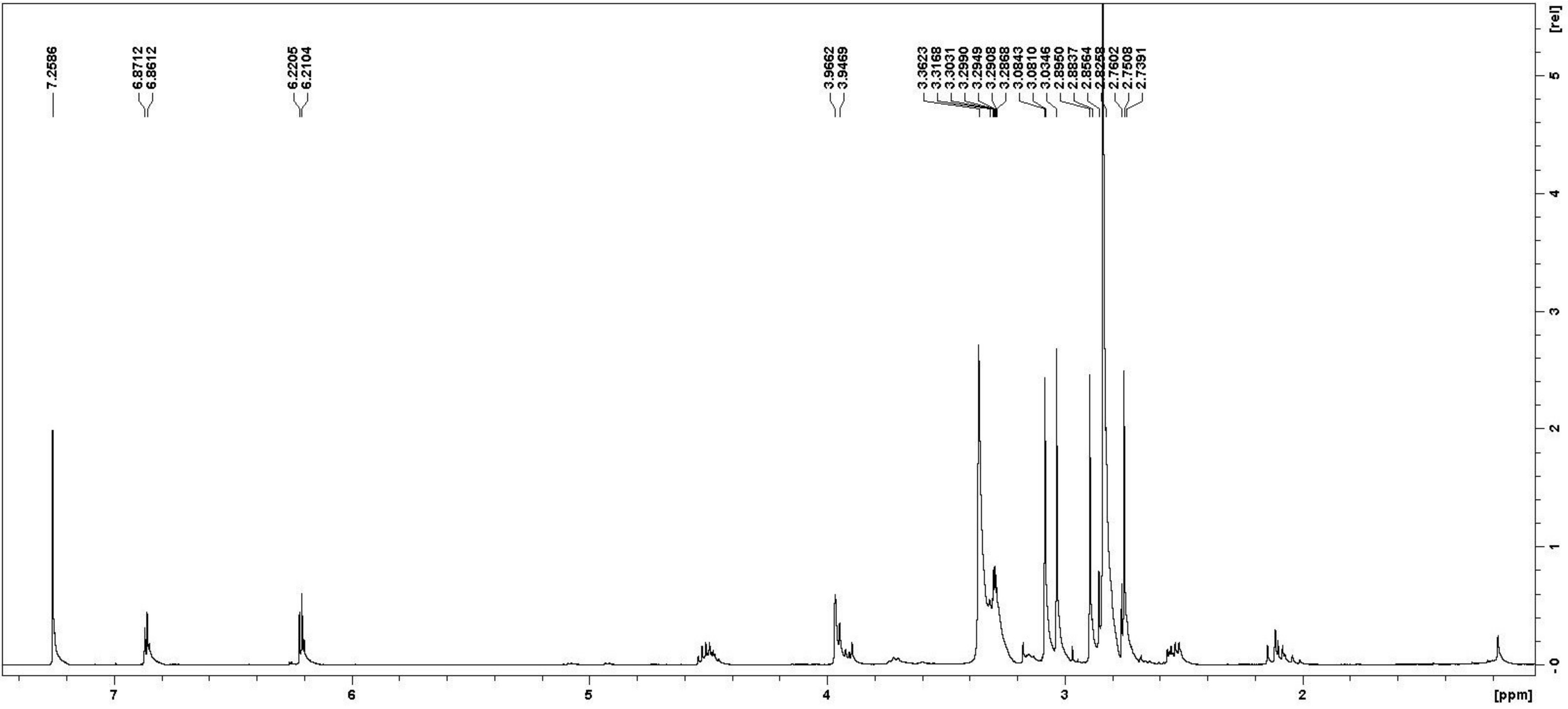

Figure S3e. – **2e** in $CHCl_3$ reflux, no strong acid

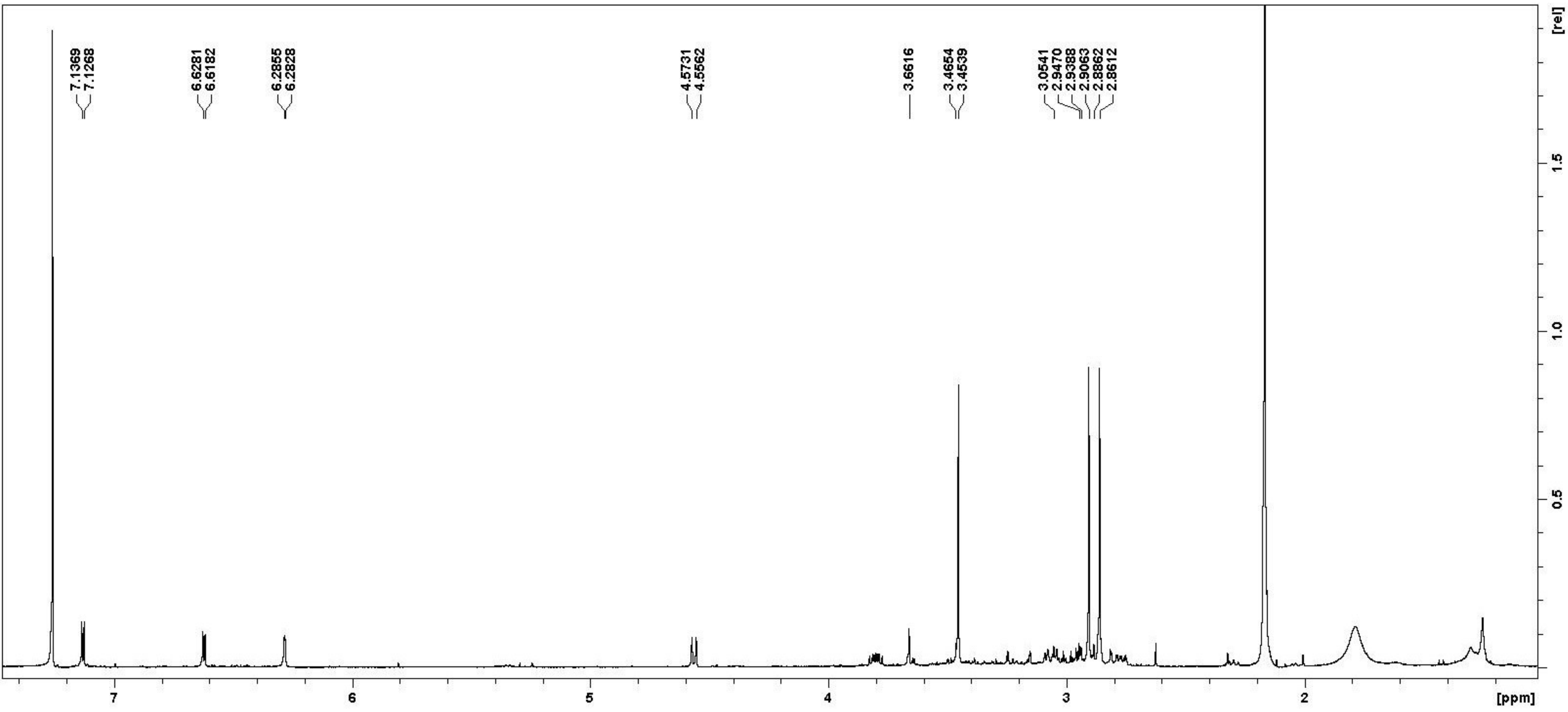

Figure S4a. – **2e** in $CHCl_3$ reflux, 1 equiv. MsOH

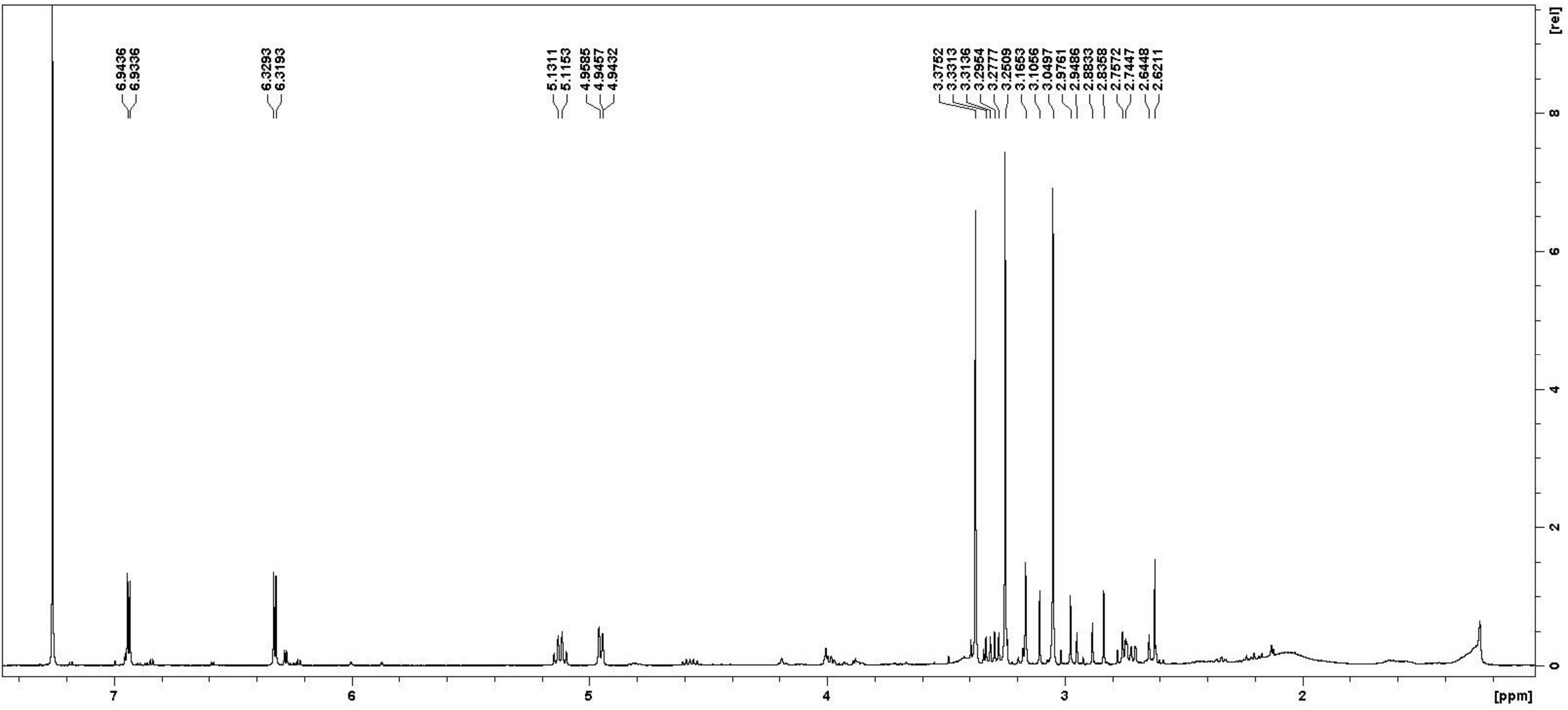

Figure S4b. – **2e** in $CHCl_3$ reflux, 0,1 equiv. MsOH, **3e**/**1e**/**4e** ~ 90:10:2

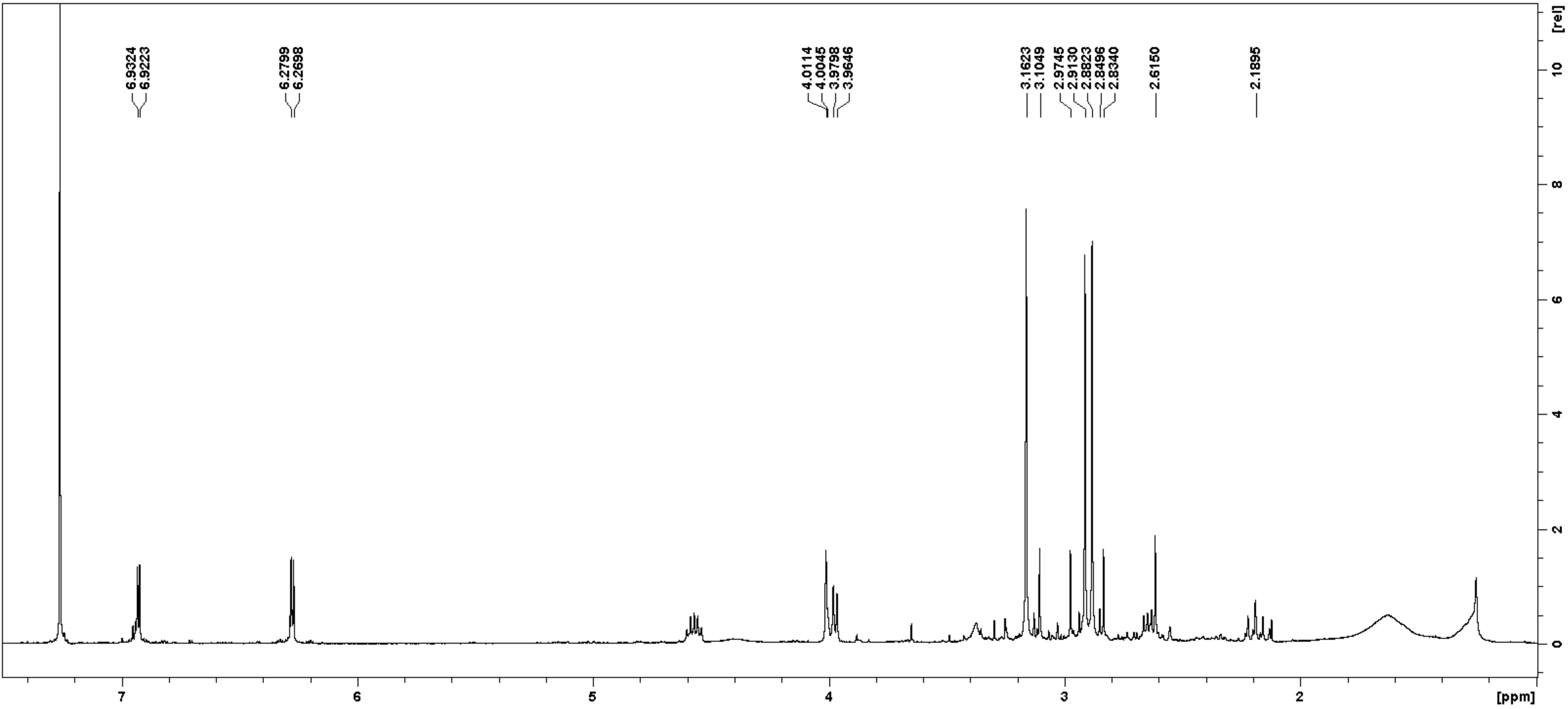

Figure S4c. – **2e** in $CHCl_3$ reflux, 0,1 equiv. MsOH after one week

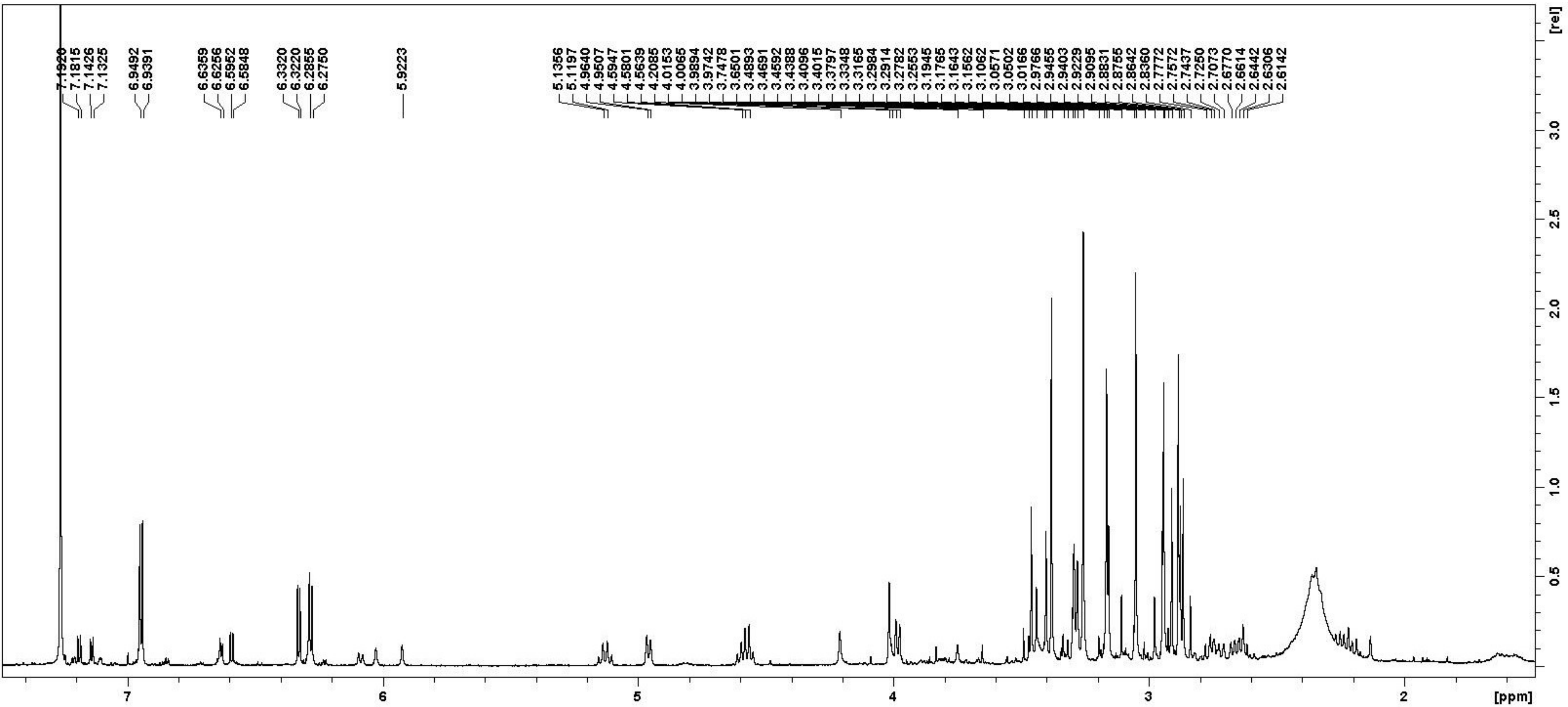

Figure S4d. – **2e** in $CHCl_3$ reflux, 0,5 equiv. MsOH, **3e** ~ **1e**/**4e**/**6e**/**5e** ~ 40:40:10:8:2

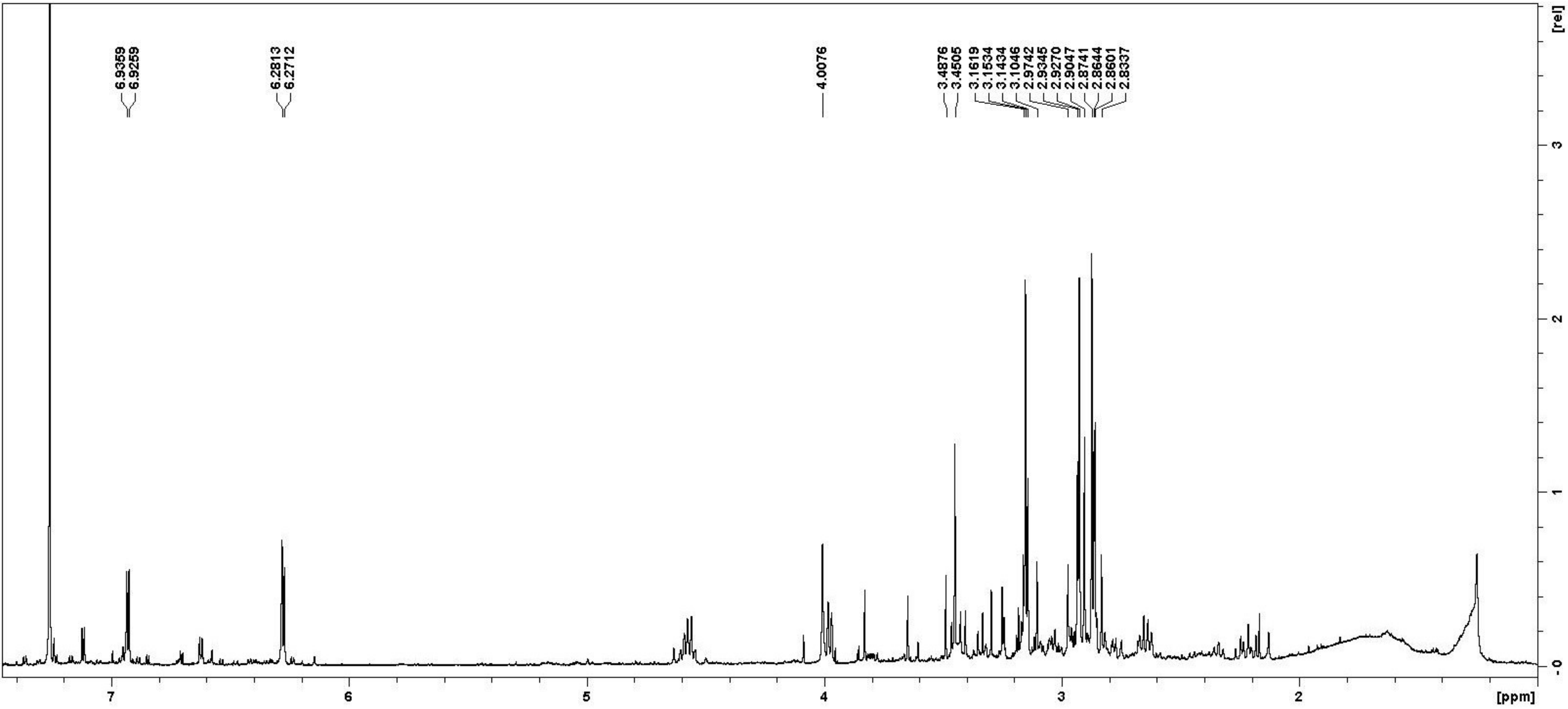

Figure S4e. – **2e** in $CHCl_3$ reflux, 0,5 equiv. MsOH after 2 weeks

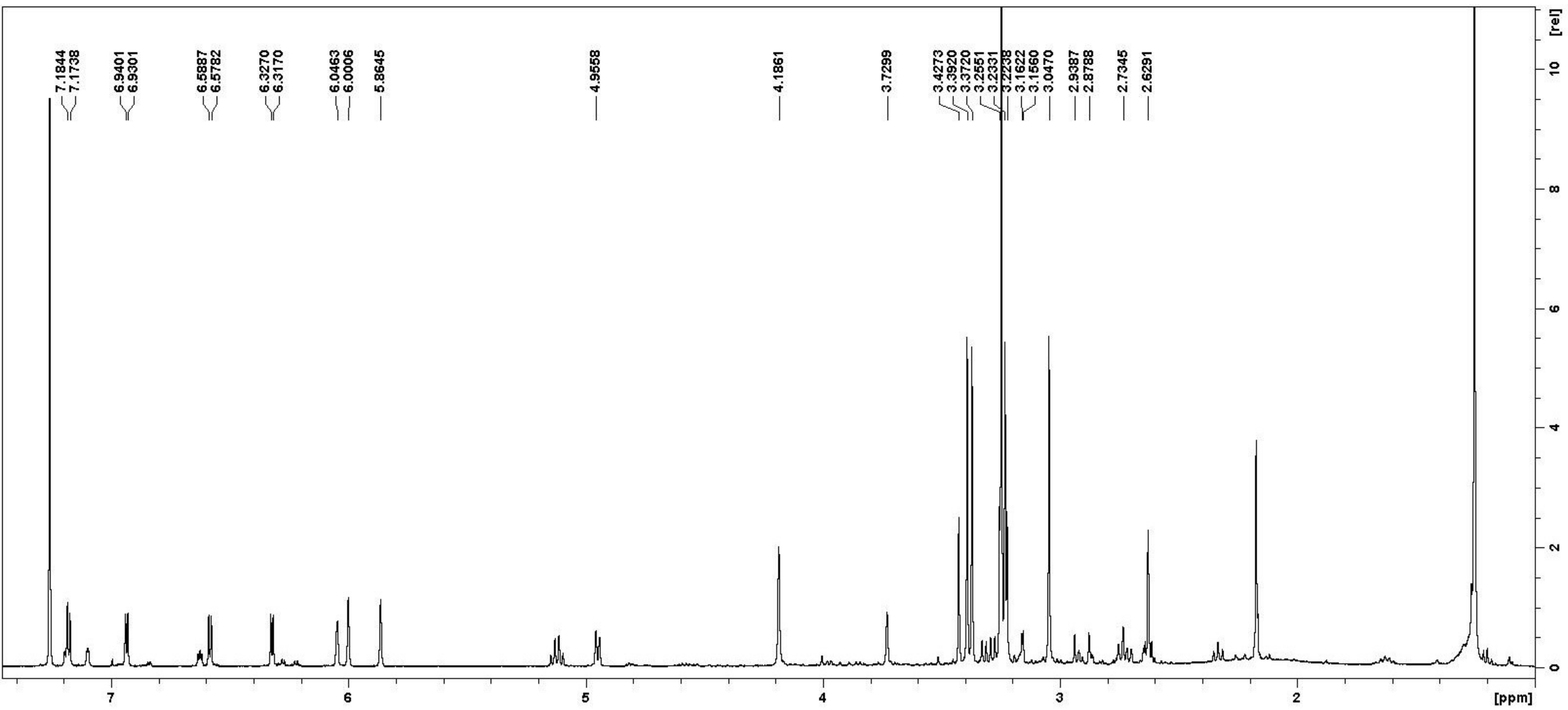

Figure S5a. – **2e** + 1 equiv. MsOH, $CHCl_3$ reflux, 15' – FC final fractions **3e**/**4e**/**5e** ~ 3:3:1

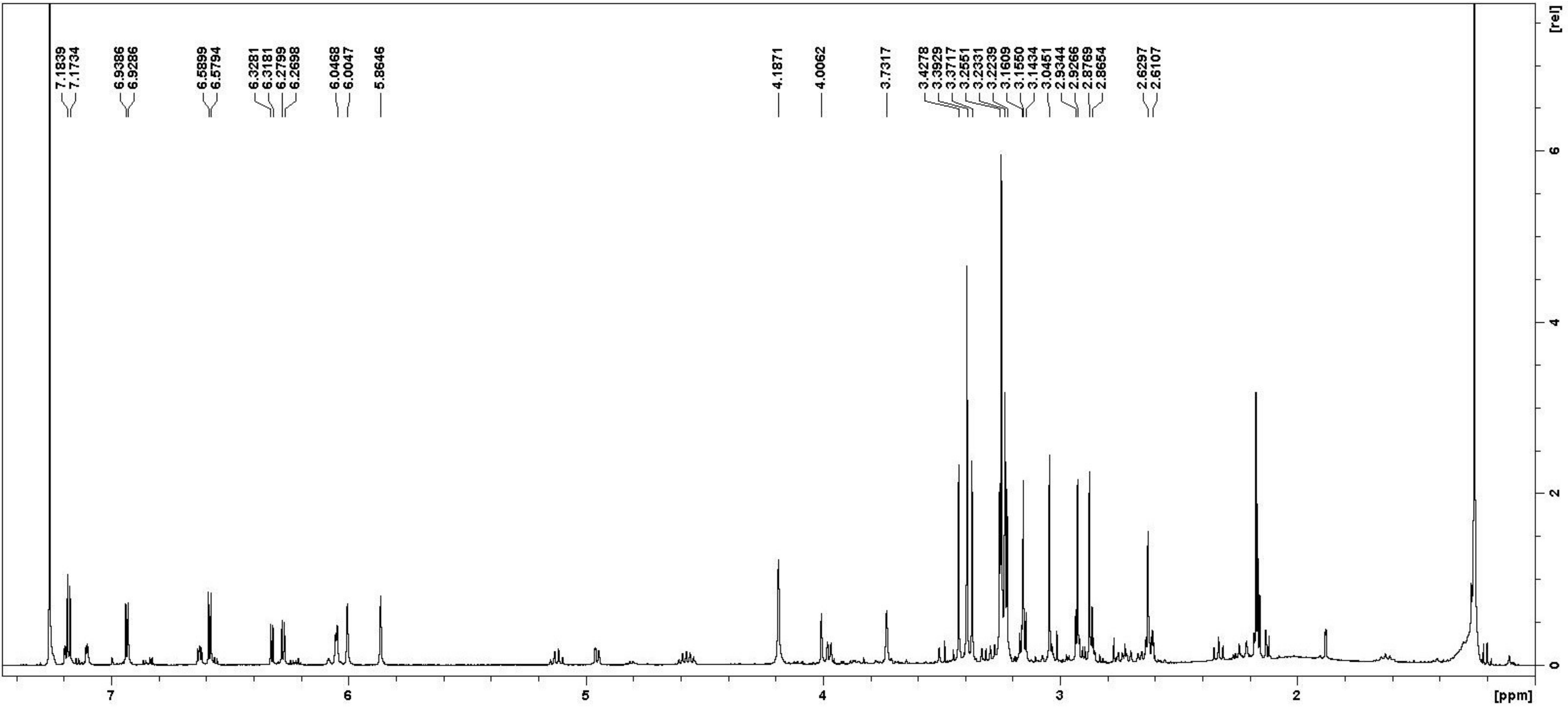

Figure S5b. – **2e** + 1 equiv. MsOH, $CHCl_3$ reflux, 15' – FC final fractions after one week **1e**/**3e**/**4e**/**5e** ~ 1.5:1.5:3:1

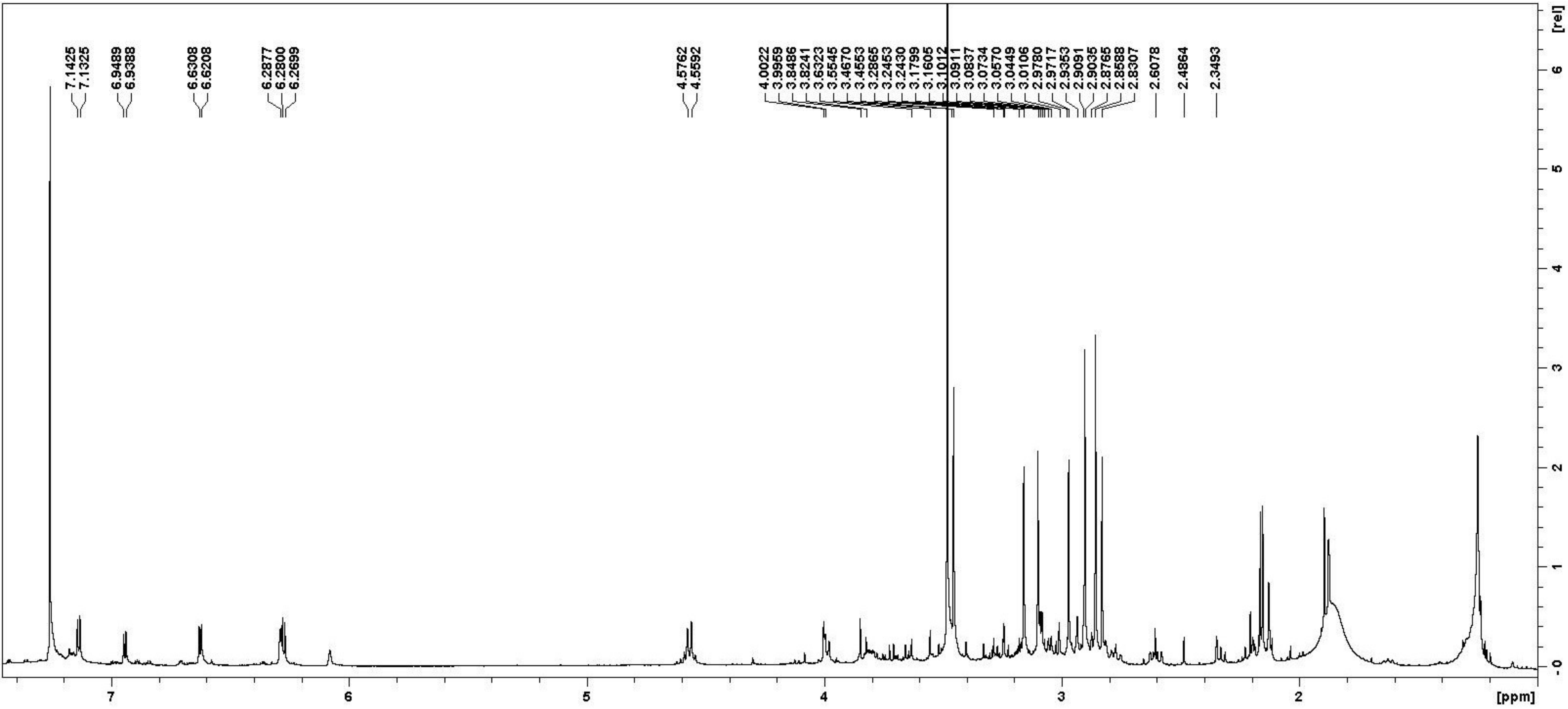

Figure S5c. – **2e** + 1 equiv. MsOH, $CHCl_3$ reflux, 15' – FC final fractions after one month **1e**/**6e** ~ 3:4

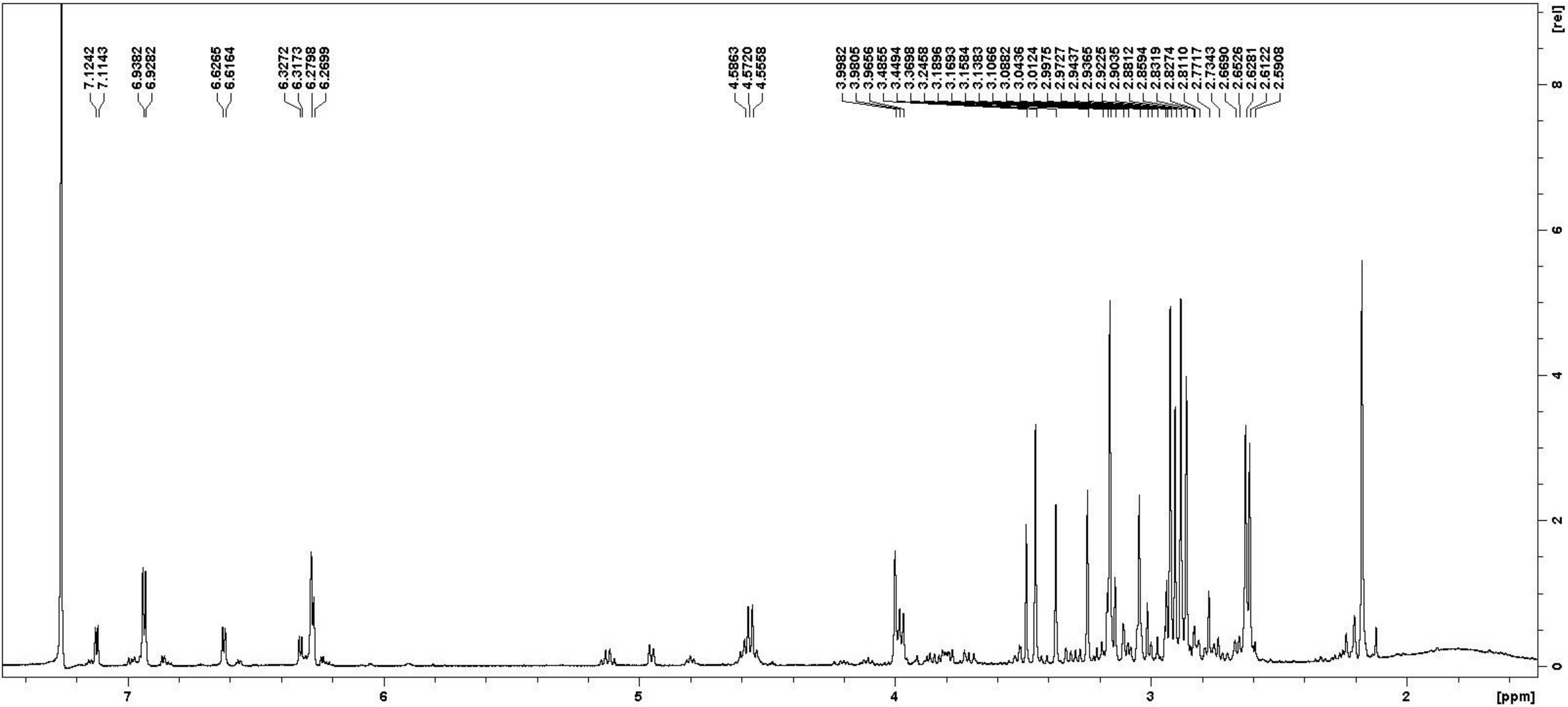

Figure S5d. – **2e** + 1 equiv. MsOH, $CHCl_3$ reflux, 15' – FC central fractions **2e** >> **6e** ~ **3e** >> **1'e**

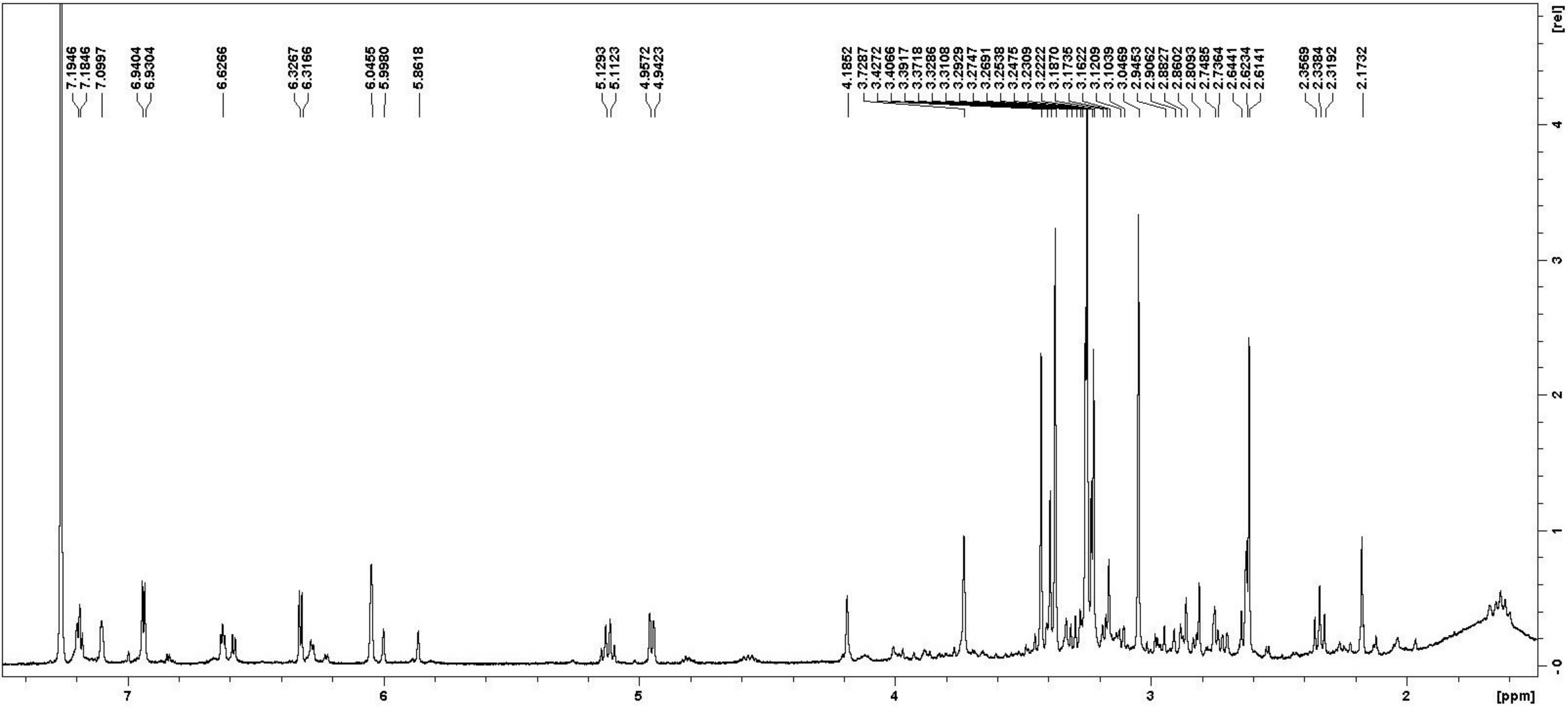

Figure S5e. – **2e** + 1 equiv. MsOH, $CHCl_3$ reflux, 15' – FC final fractions **3e**/**5e**/**4e** ~ 4:2.5:1

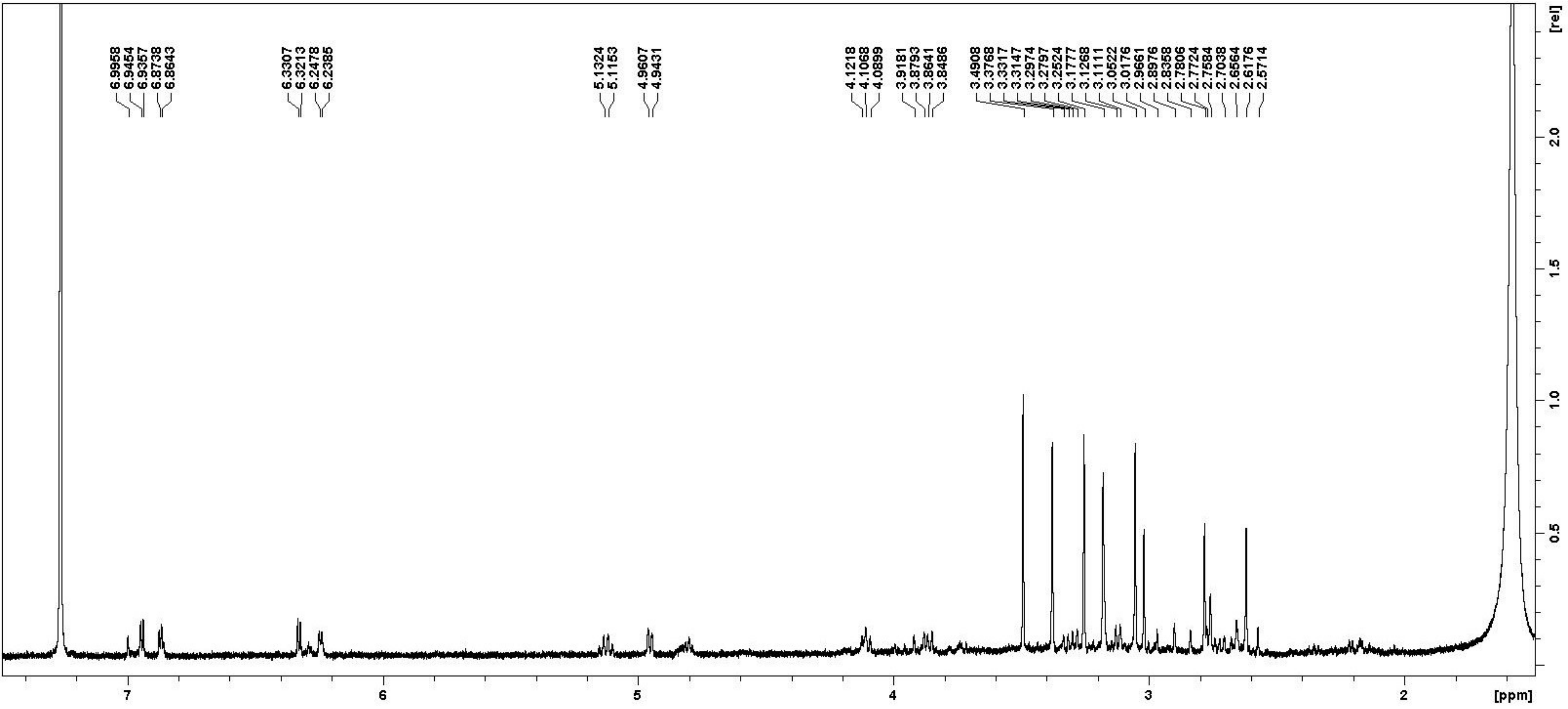

Figure S5f. – **2e** + 1 equiv. MsOH, $CHCl_3$ reflux, 15' – FC ff than HPLC $\mathbf{t_R}$ = 9' (**3e** + **1'e**)

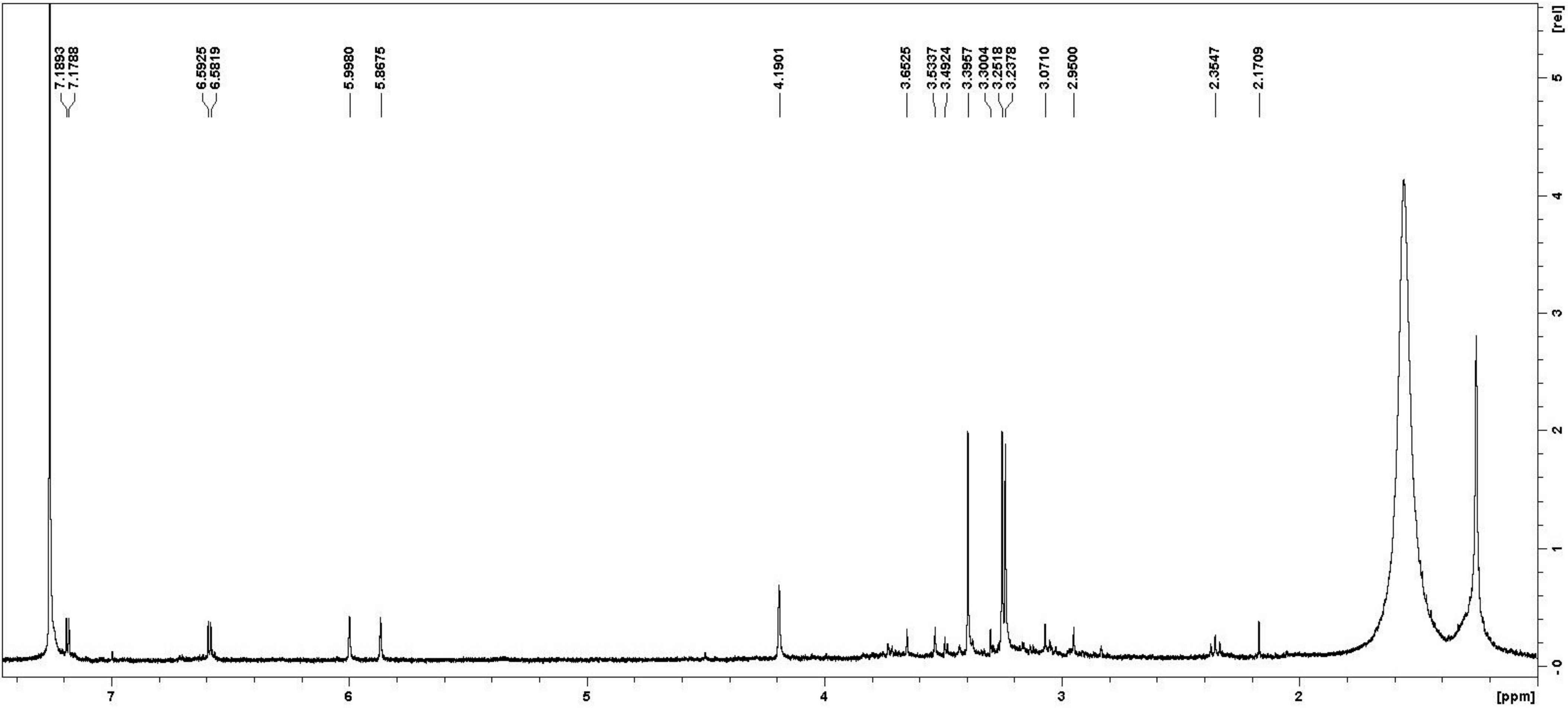

Figure S5g. – **2e** + 1 equiv. MsOH, $CHCl_3$ reflux, 15' – FC ff than HPLC $\mathbf{t_R}$ = 15' **4e**

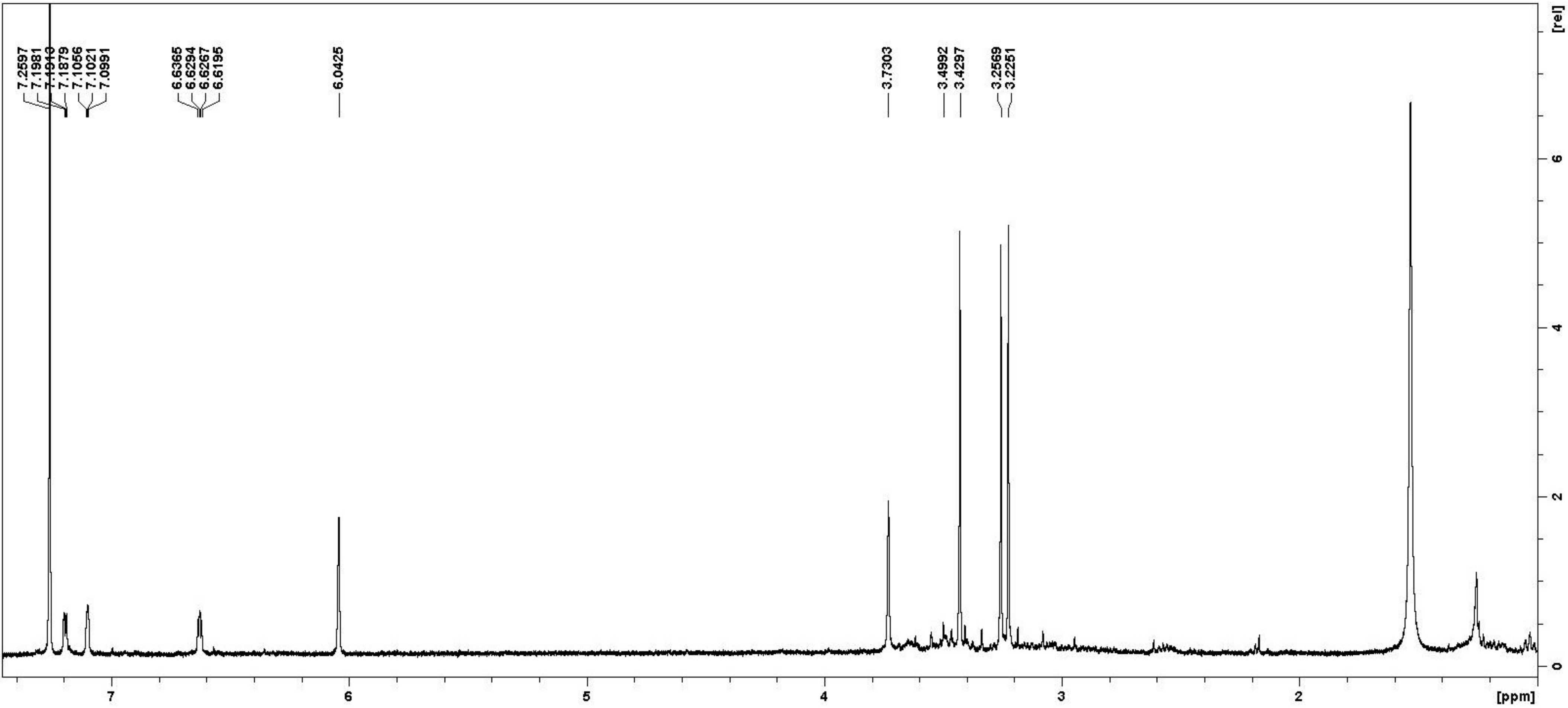

Figure S5h. – **2e** + 1 equiv. MsOH, $CHCl_3$ reflux, 15' – FC ff than HPLC $\mathbf{t_R}$ = 19' **5e**

Reaction	Conditions	Compounds by ^{1}H-NMR	Compound, t_R and possible $[M+H]^+$ (*m/z*) by LC-DAD-MS experiments. Style by UV bands.			
-	Mix of known compounds	**6e** **1** **1d** **1m** **1e** **2m** **2e**	**6e**/14.5 **1**/15.9 **1d**/17.6 **1m**/19.1 **1e**/21.3 **2m**/25.5 **2e**/29.2			
R6	**2e** + 0.1eq.MsOH, CHCl3, 70°, 24h	**4e**(2%) **3e**(90%) **2e**(10%)	**2'e**/19.9	**4e**/24.2	***3e**/25.9*	**2e**/28.6
R7	**2e** + 0.5eq.MsOH, CHCl3, 70°, 24h	**3e** ~ **1e** 40% -- **4e** 10% -- **6e** 8% -- **5e** (2%)	**1'e**/13.6(369-71)<br>**1e**/20.3(369-71)	**6e**/14.1(273)<br>**4e**/23.9(351-53)	**5e**/14.4(273)<br>***3e**/25.7(351-53)*	**2'e**/19.5(383-85)<br>**2e**/28.4(383-85)
R8	**2e** + 1.1eq.MsOD, CDCl3, 70°, 1.5h	**6e** (70%), **5e** (30%)		**6e**/14.1(273)	**5e**/14.3(273)	
R9	**2e** + 0.8eq.MsOH, CHCl3, 70°, 1.2h	(First aliquot after 15')		**6e**/14.1<br>**4e**/23.9	**5e**/14.3<br>***3e**/25.7*	**2e**/28.3
		**6e** (95%), **5e** (5%)		**6e**/14.1(273)	**5e**/14.3(273)	

Figure S6a. Table 1. Comparison of four reactions according to chromatographic and spectroscopic results. Retention times may differ slightly depending on eluent preparation and day of acquisition.
Style normal: substrates $\lambda_{max} \sim 200, 230, 280$ and $\varepsilon_{200} > \varepsilon_{280} > \varepsilon_{230}$; style underline: products $\lambda_{max} \sim 230, 280$ and $\varepsilon_{230} > \varepsilon_{280}$; style italics: compound $\lambda_{max} \sim 200, 230, 280$ and $\varepsilon_{200} > \varepsilon_{230} > \varepsilon_{280}$.

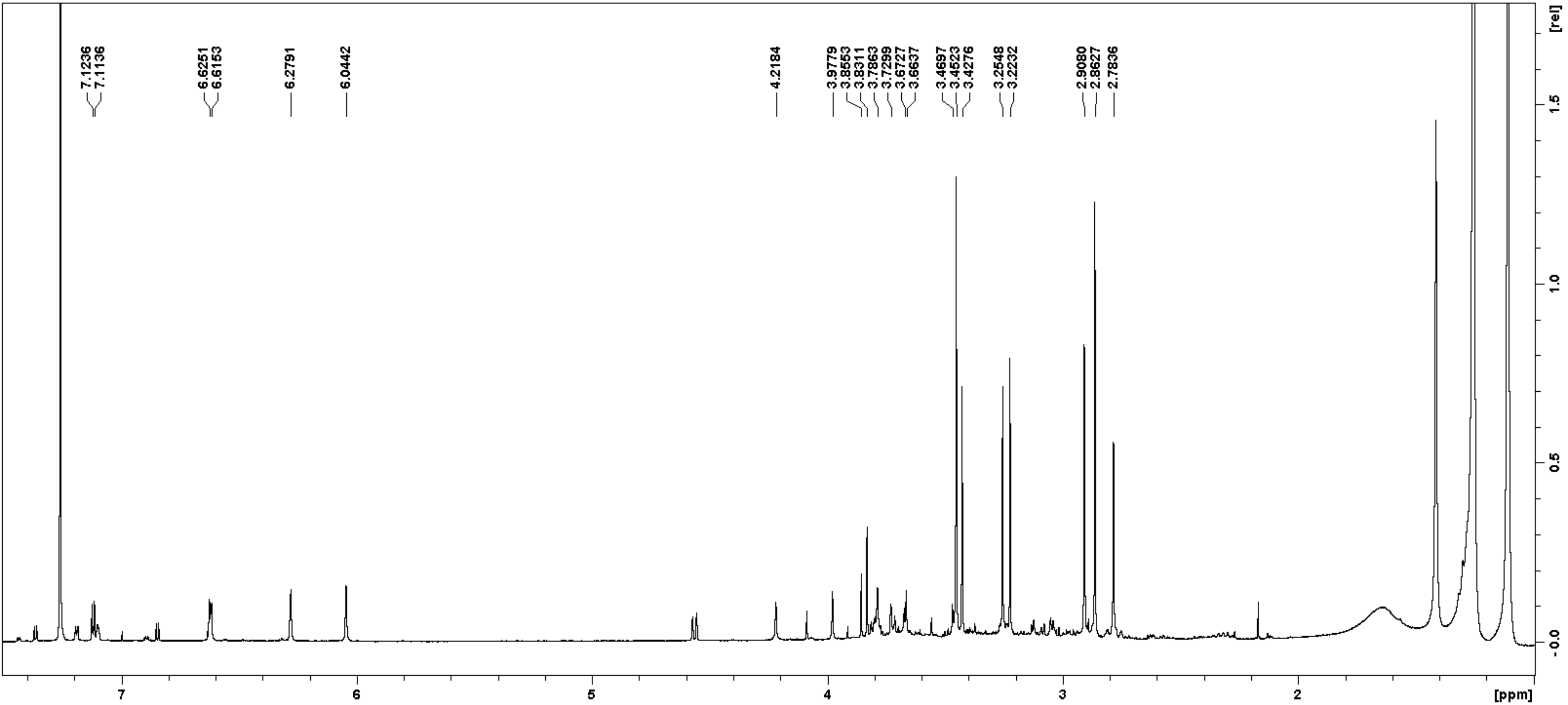

Figure S6b. – **2e** in $CDCl_3$ reflux, 1.1 equiv. MsOD, 90' **6e**/**5e** ~ 70:30 ; see also **S4b-e**

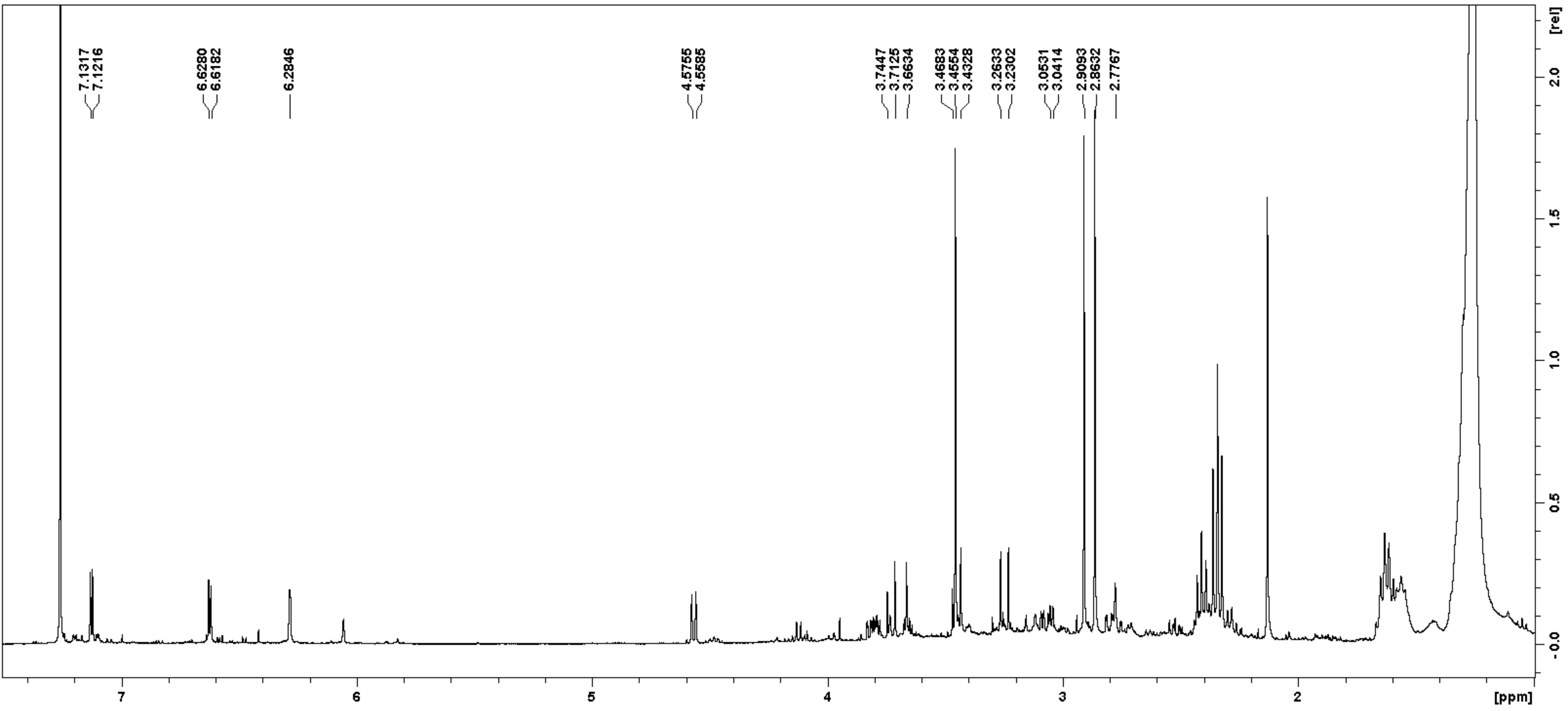

Figure S6c. – **2e** in $CHCl_3$ reflux, 0,8 equiv. MsOH, 75' **6e**/**5e** ~ 95:5

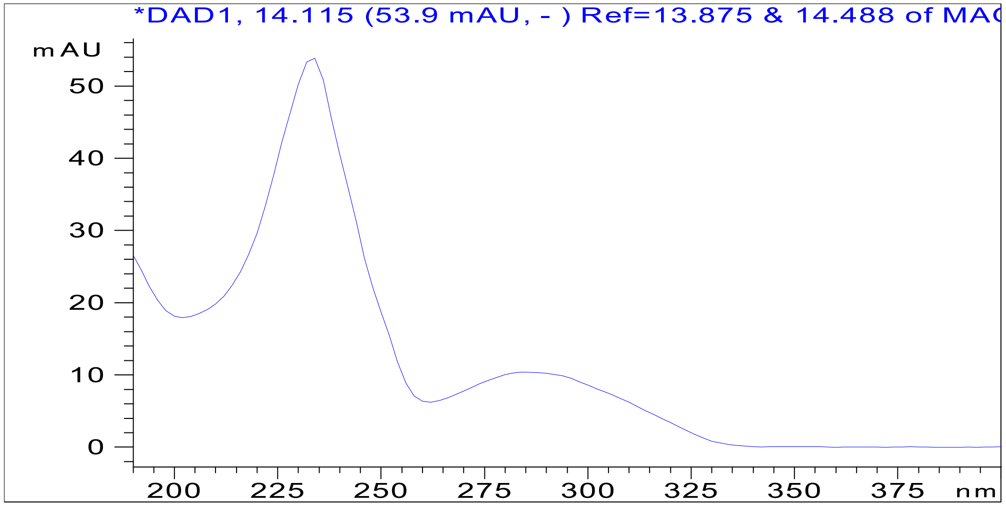

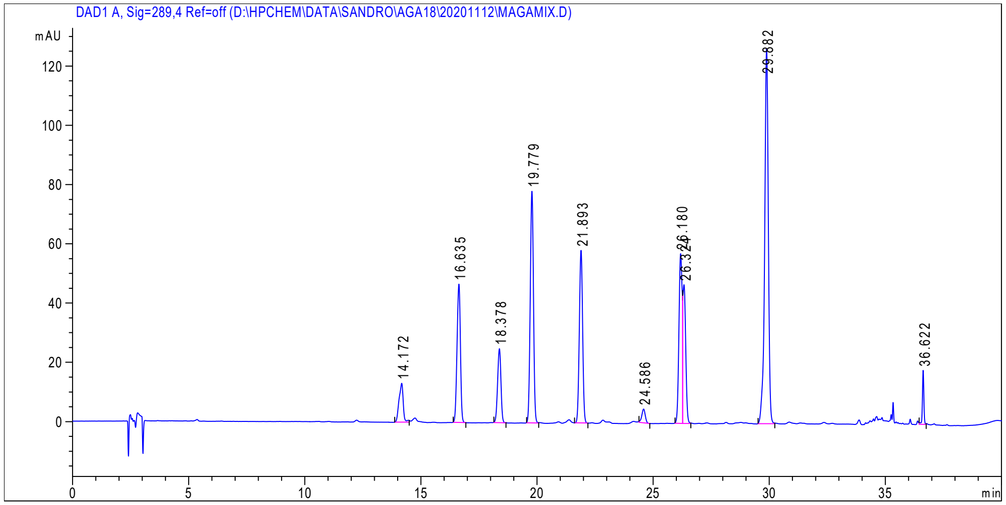

Figure S6d. HPLC-DAD UV spectrum of **6e**

HPLC chromatogram at λ = 289 nm of **6e**, **1**, **1d**, **1m**, **1e**, **2m**, **2e**,

HPLC-DAD UV spectrum of **1d**

HPLC-DAD UV spectrum of **1**

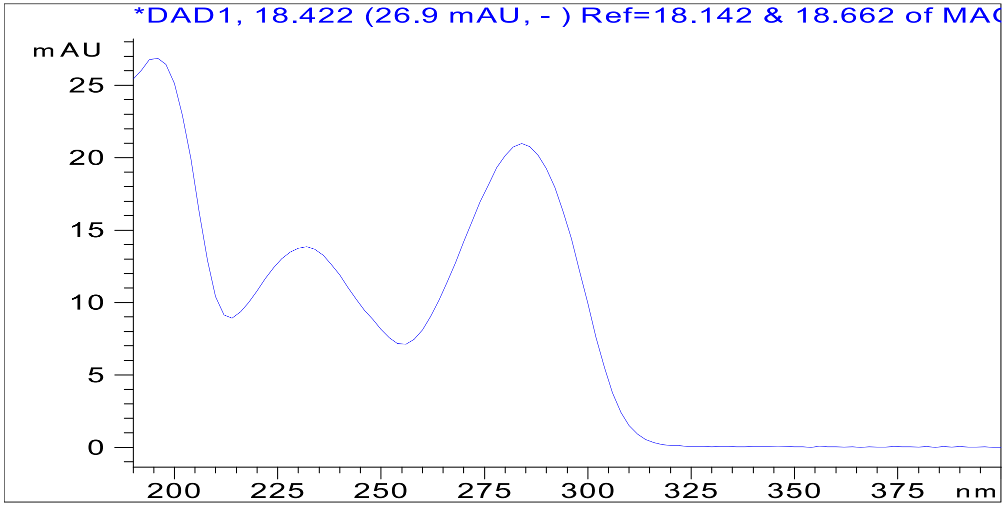

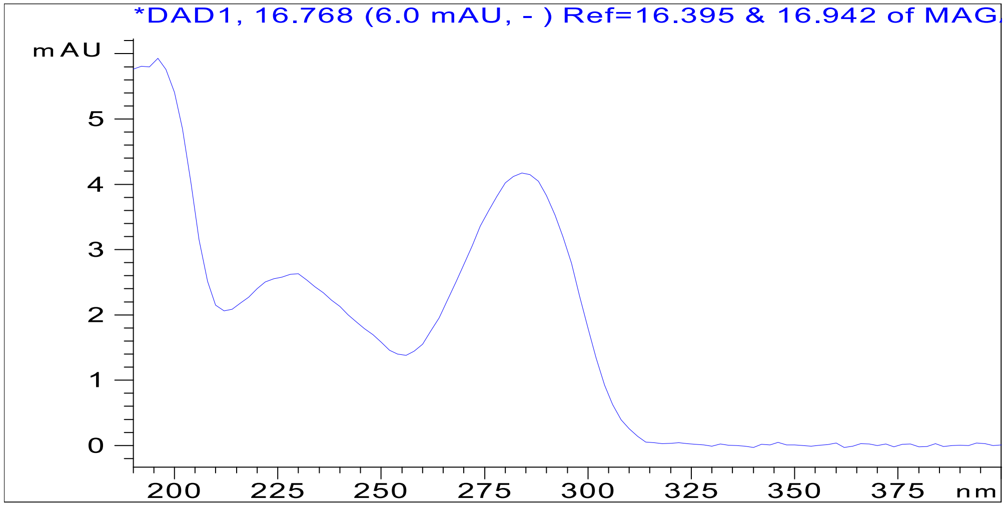

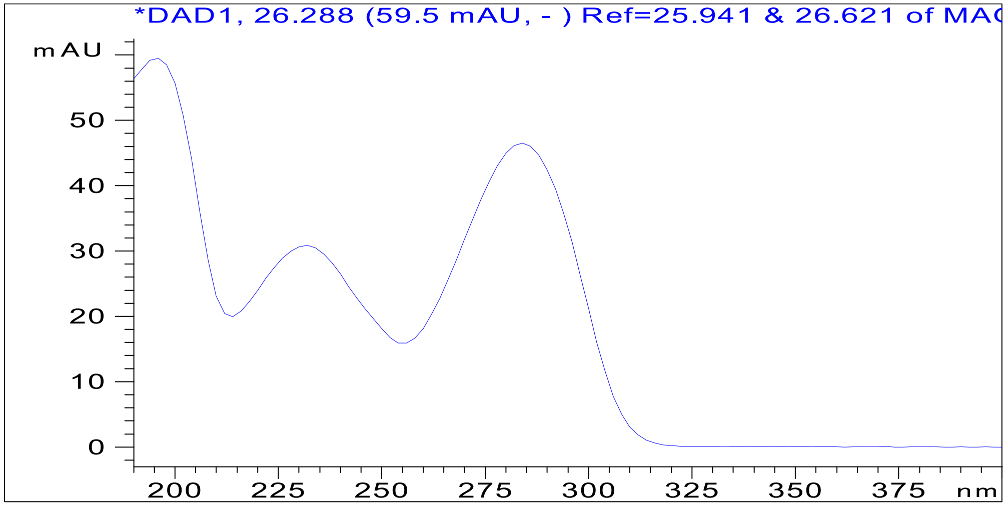

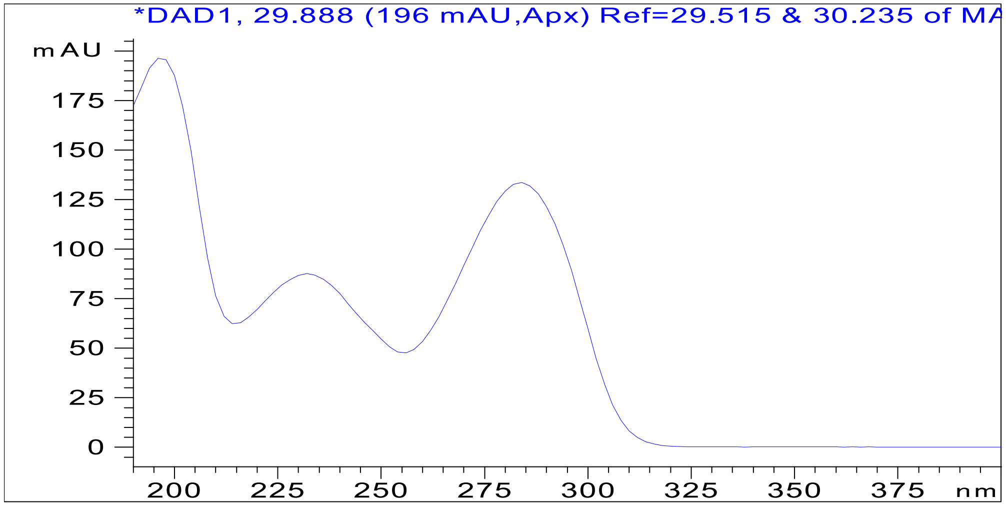

Figure S6e. HPLC-DAD UV spectrum of **2m**

HPLC-DAD UV spectrum of **1m**

HPLC-DAD UV spectrum of **2e**

HPLC-DAD UV spectrum of **1e**

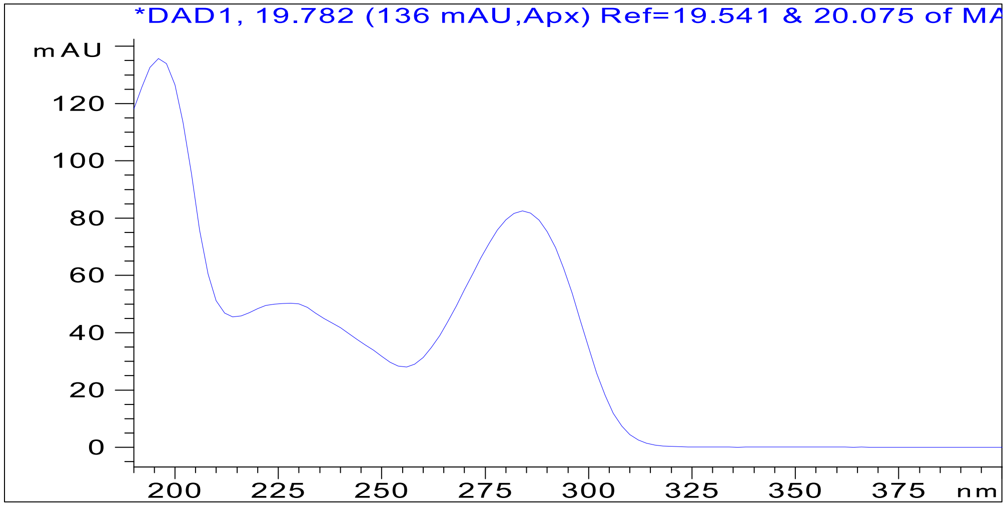

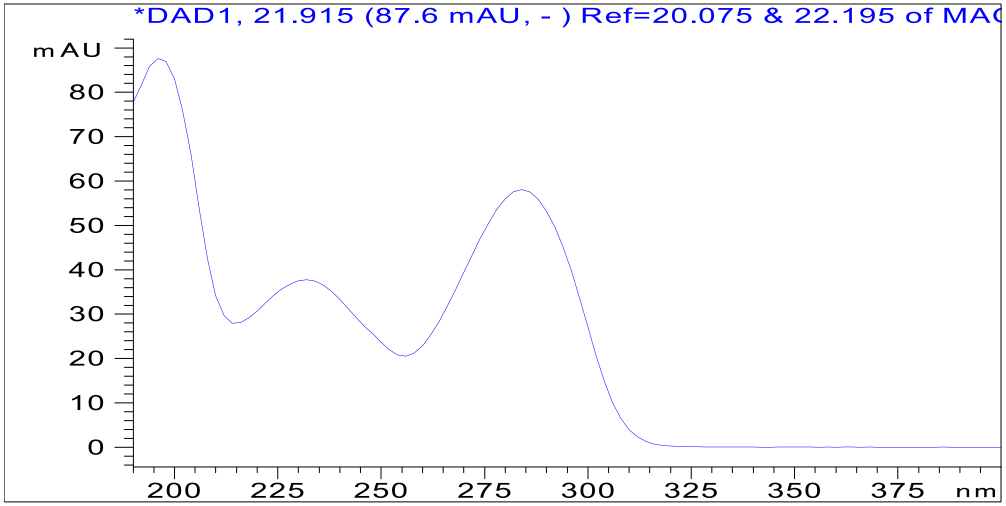

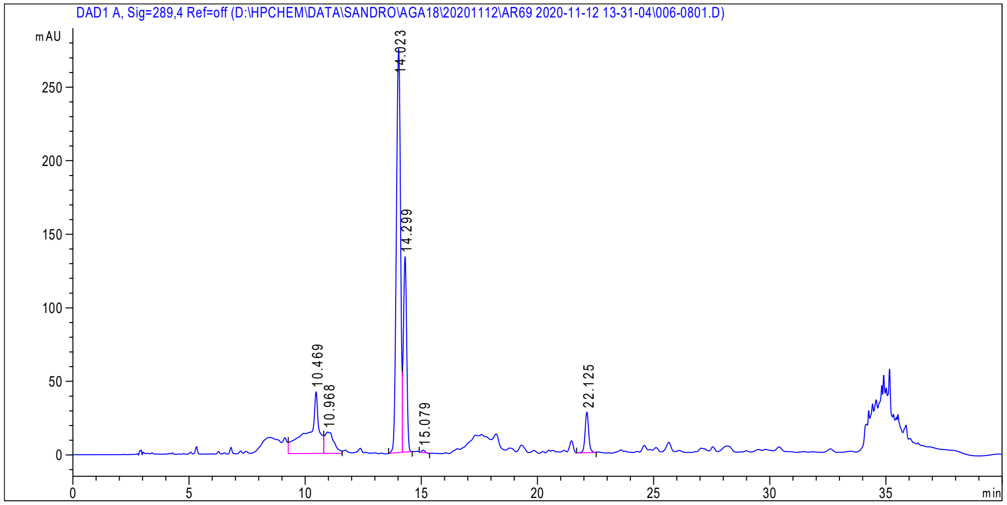

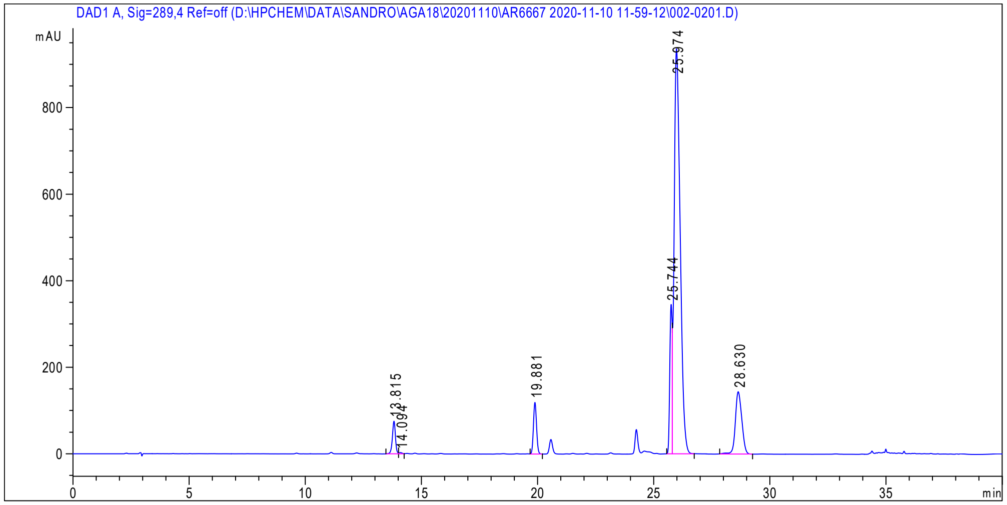

Figure S6f. HPLC chromatogram at λ = 289 nm of **R8**
HPLC chromatogram at λ = 289 nm of **R9-1**

HPLC chromatogram at λ = 289 nm of **R6**
HPLC chromatogram at λ = 289 nm of **R7**

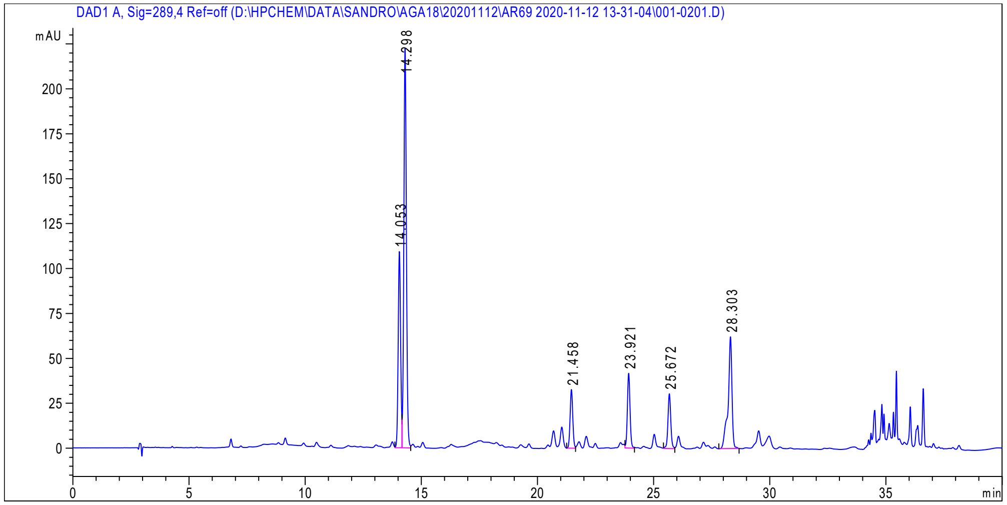

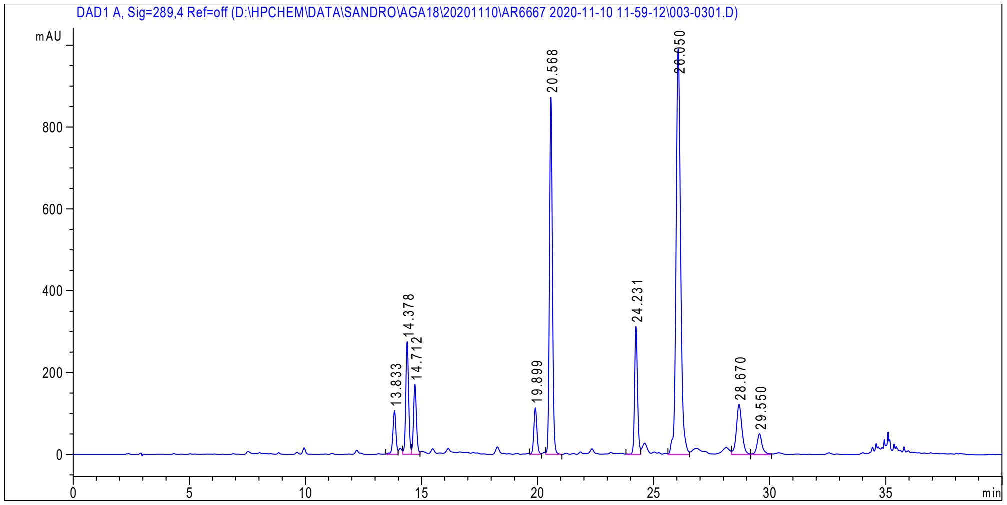

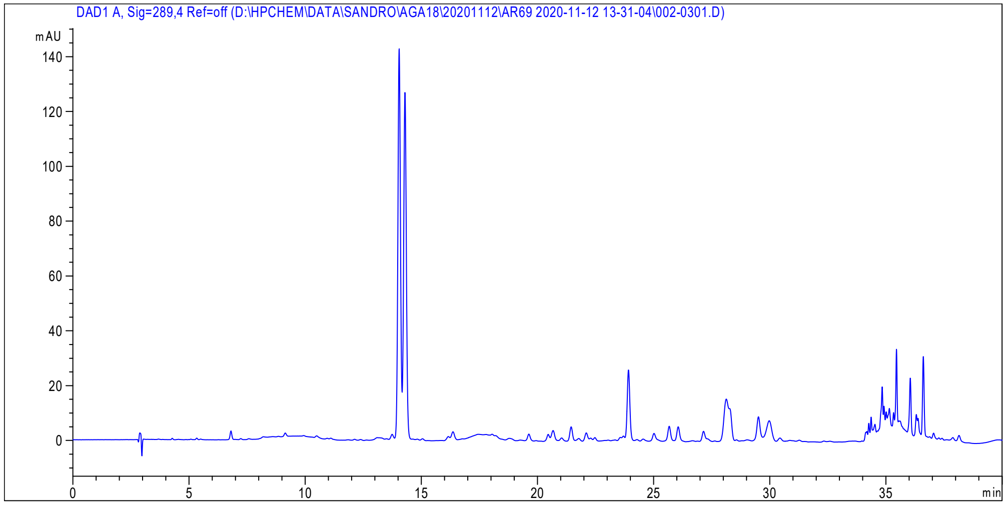

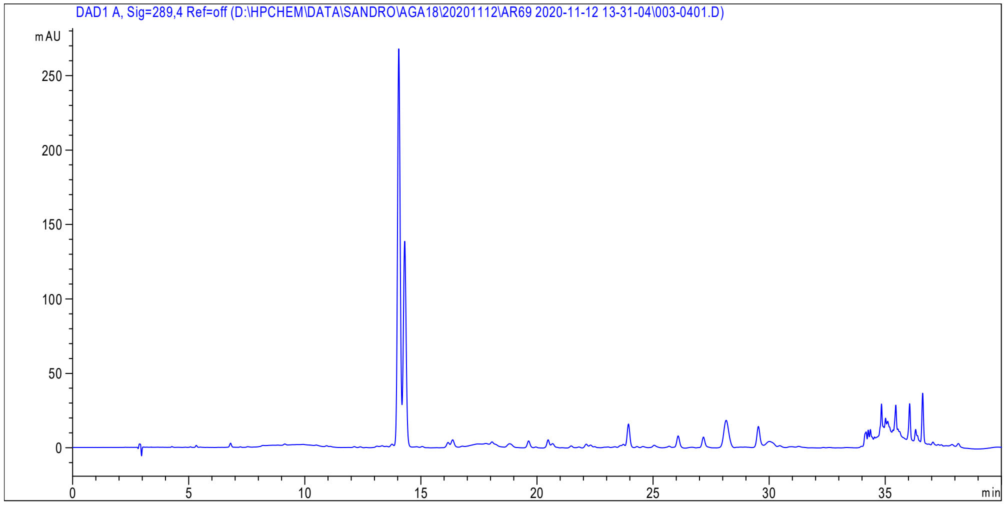

Figure S6g. HPLC chromatogram at λ = 289 nm of **R9-2**
HPLC chromatogram at λ = 289 nm of **R9-4**

HPLC chromatogram at λ = 289 nm of **R9-3**
HPLC chromatogram at λ = 289 nm of **R9-5**

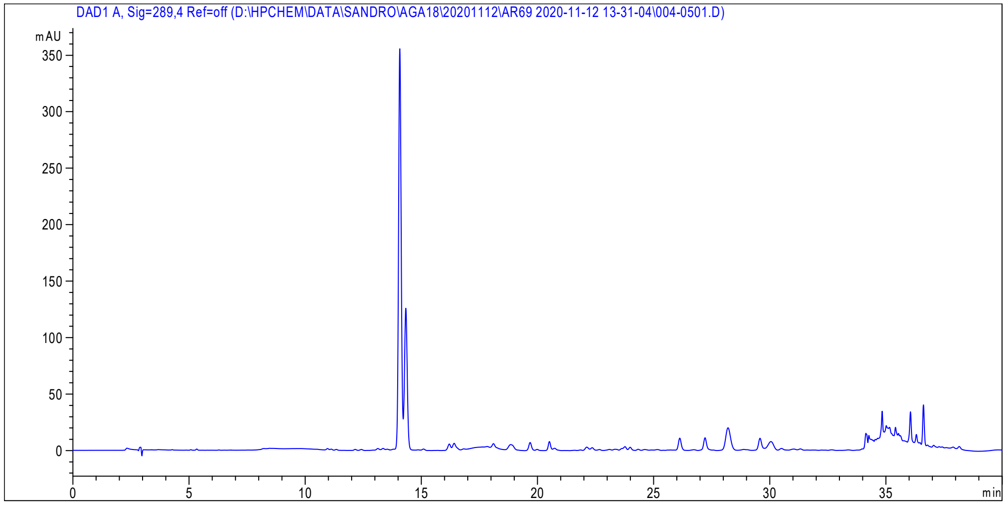

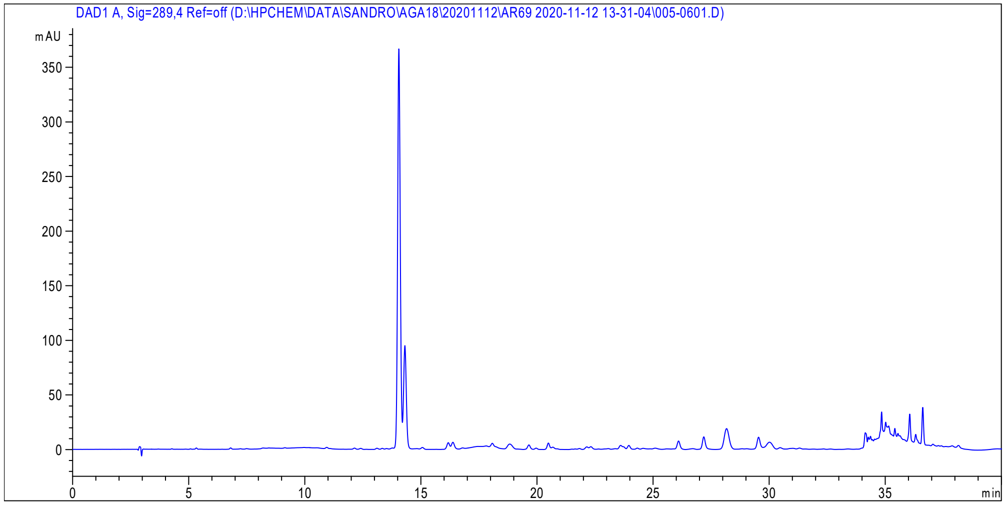

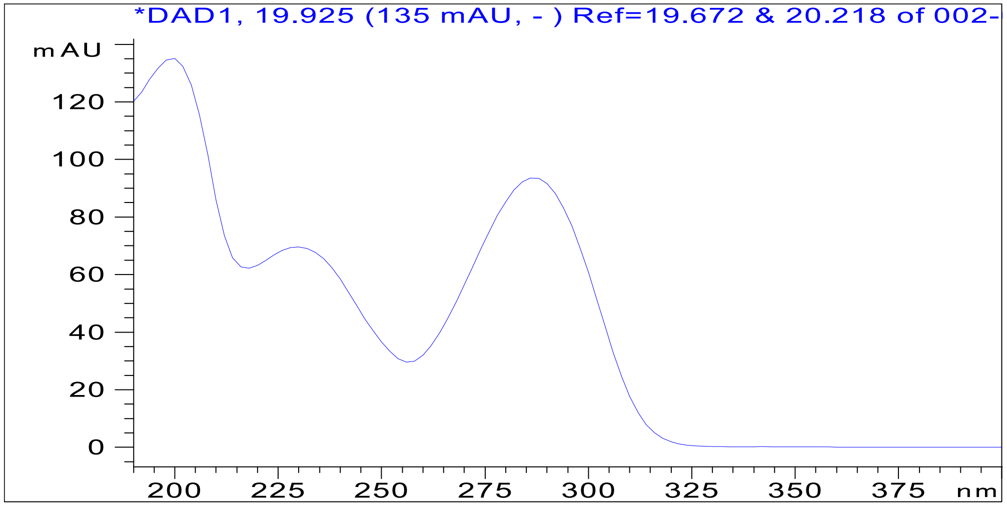

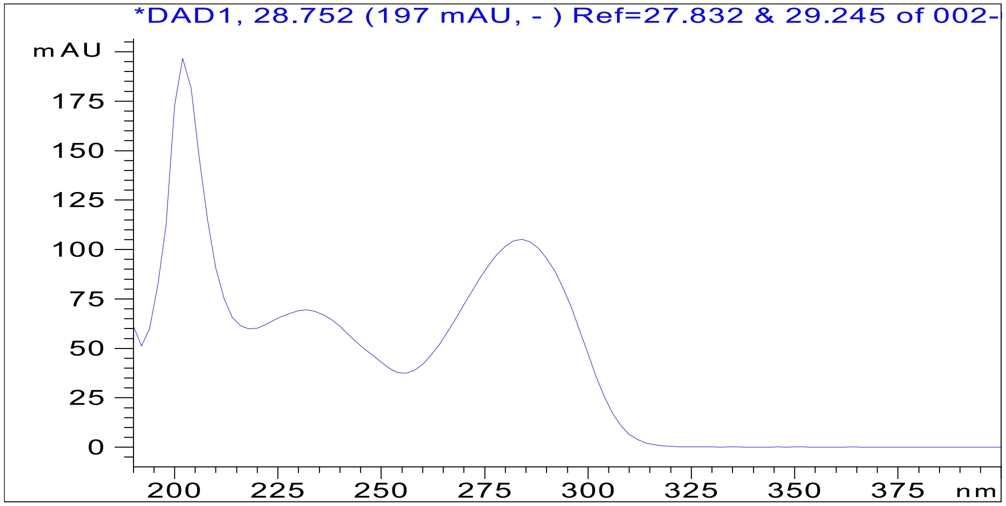

Figure S6h. HPLC-DAD UV spectrum of **R6 2'e** HPLC-DAD UV spectrum of **R6 2e**

HPLC-DAD UV spectrum of **R6 4e** HPLC-DAD UV spectrum of **R6 3e**

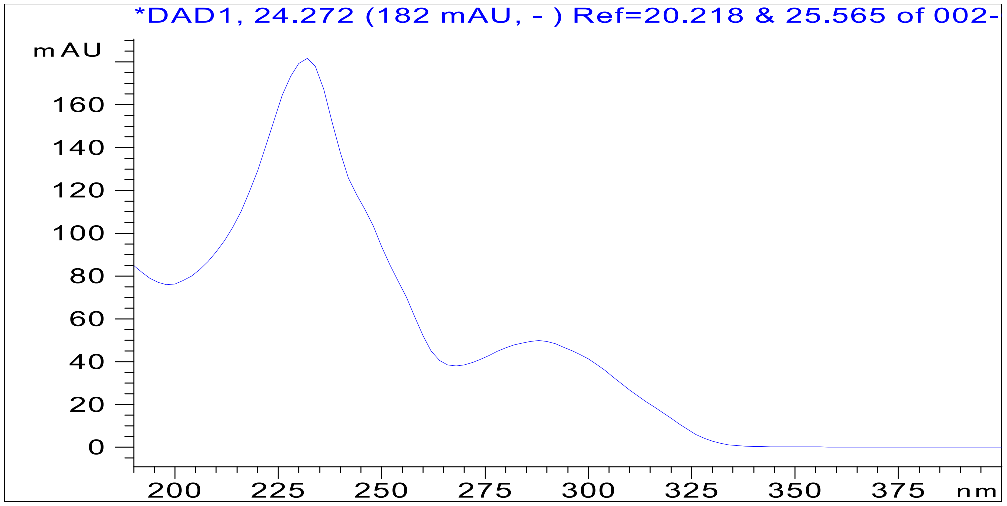

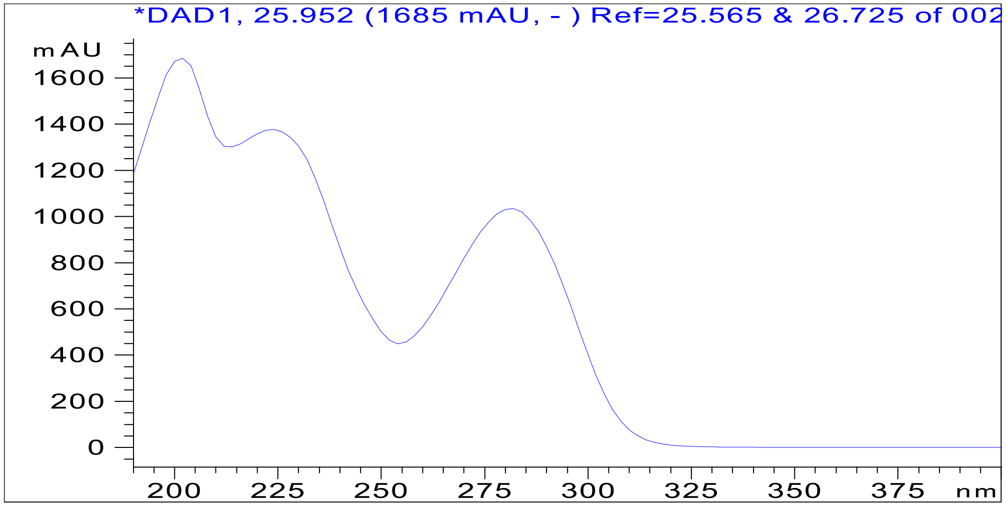

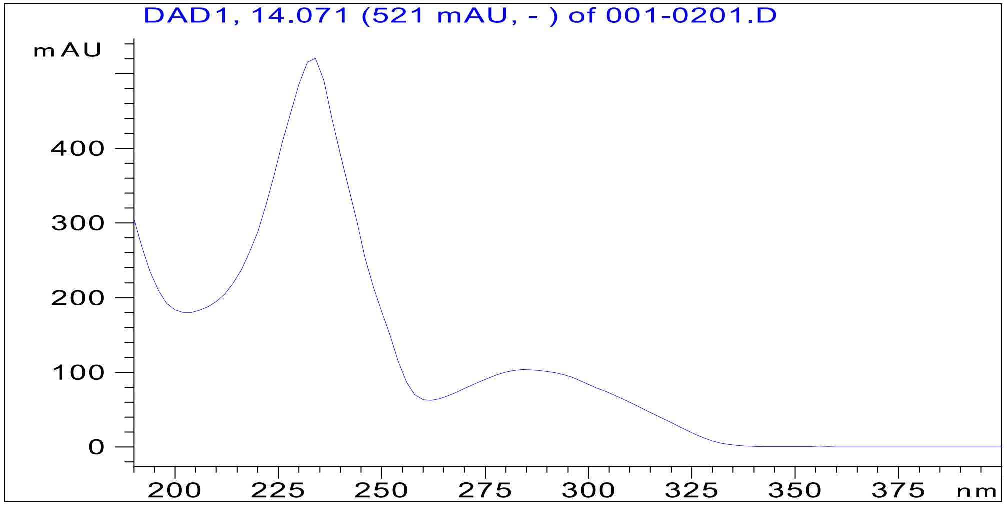

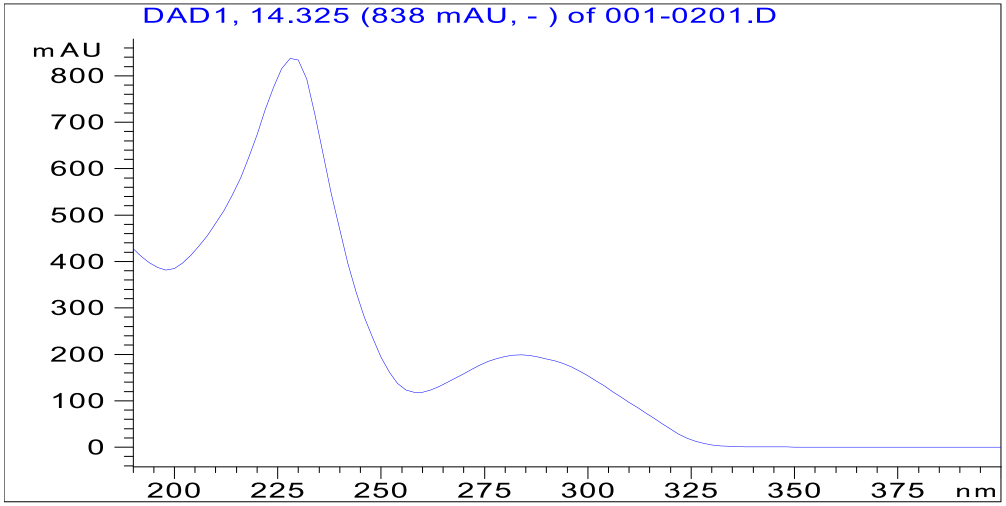

Figure S6i.

HPLC-DAD UV spectrum of **R9-1 6e**

HPLC-DAD UV spectrum of **R9-1 5e**

HPLC-DAD UV spectrum of **R9-1 4e**

HPLC-ELSD chromatogram of **R7**

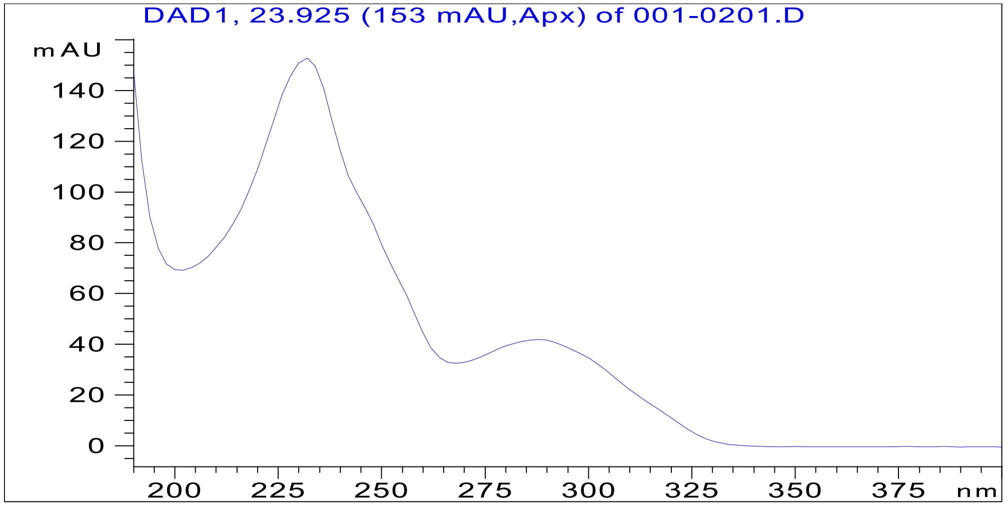

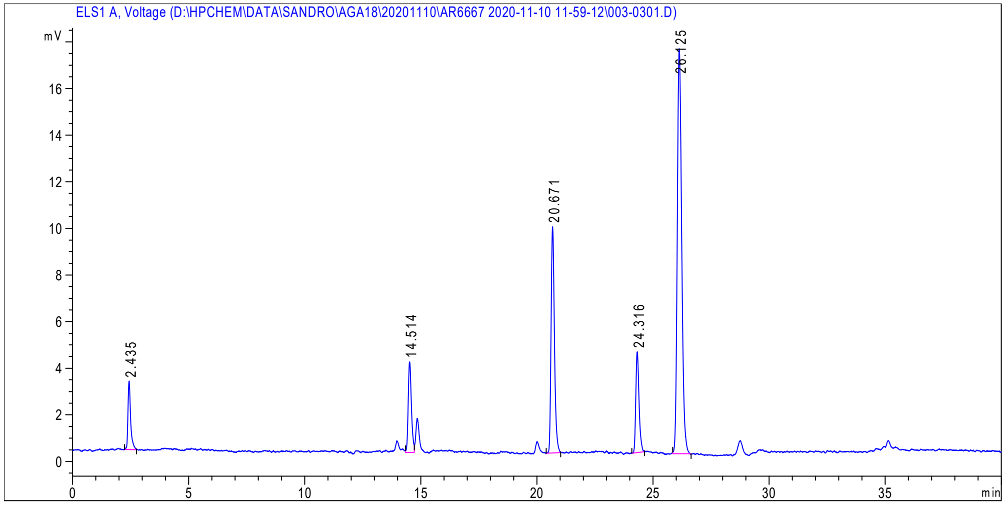

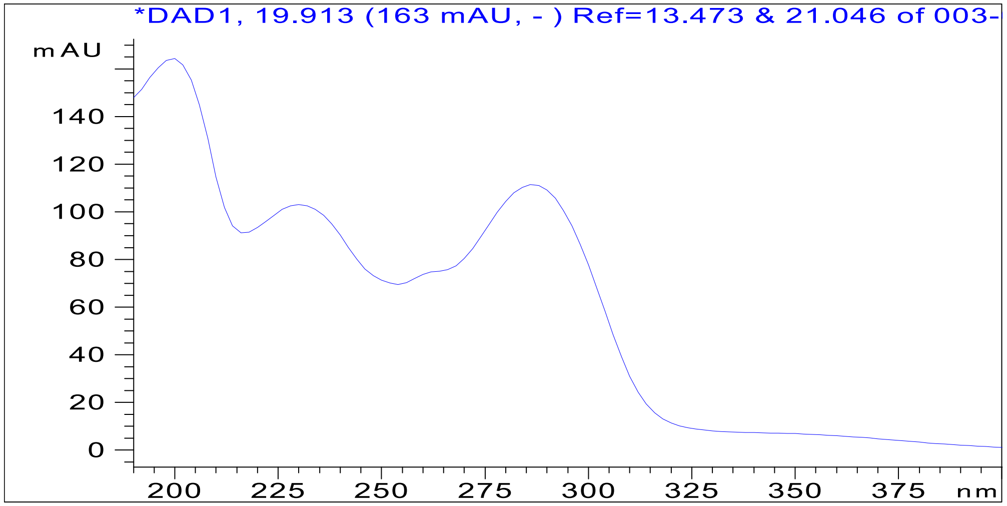

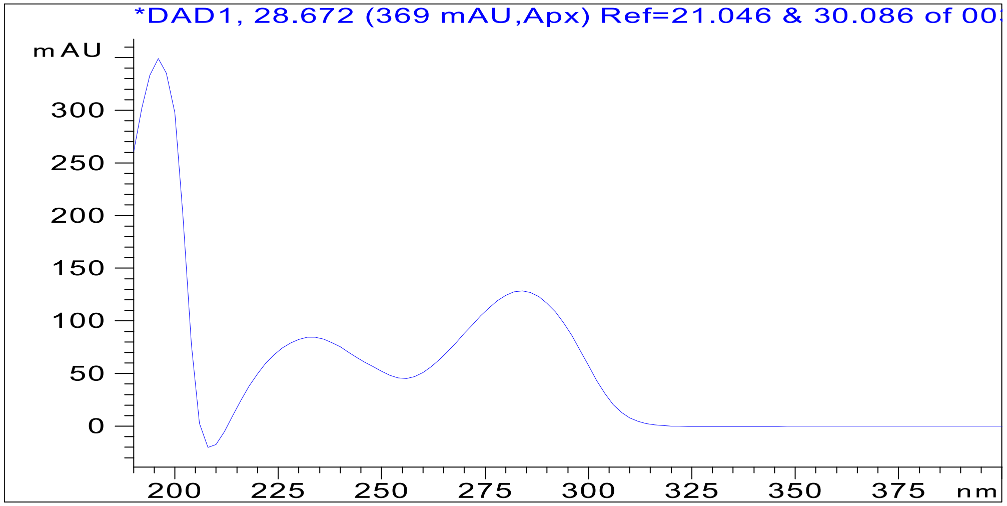

FigureS6j. HPLC-DAD UV spectrum of **R7 2'e**

HPLC-DAD UV spectrum of **R7 2e**

HPLC-DAD UV spectrum of **R7 1'e**

HPLC-DAD UV spectrum of **R7 1e**

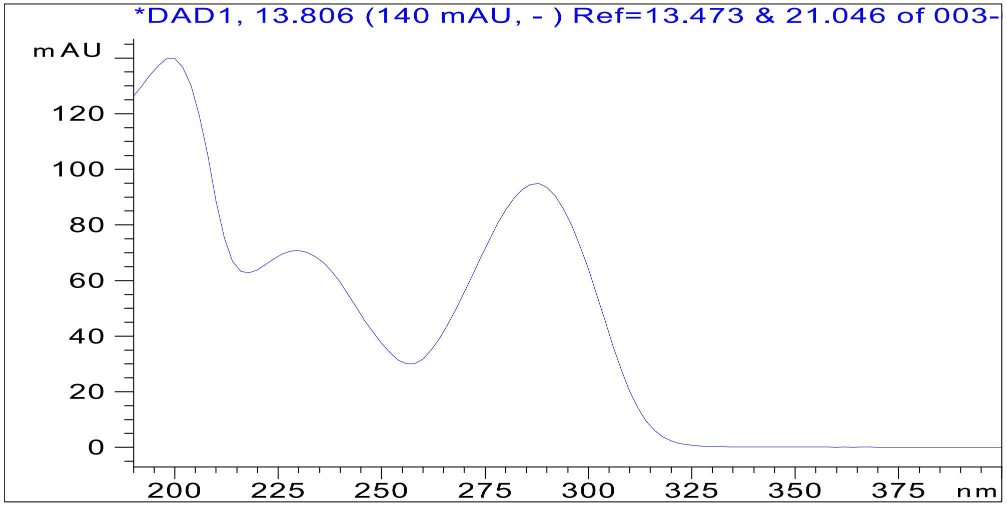

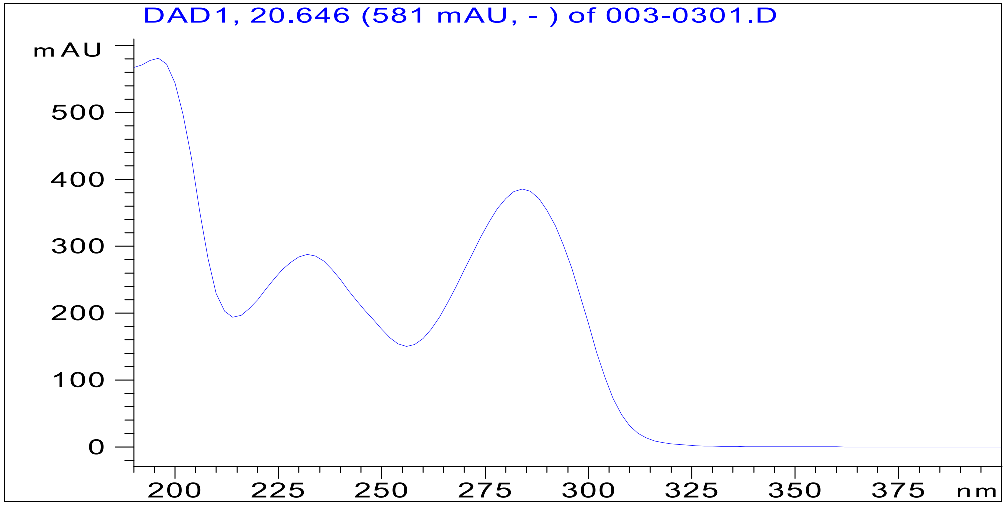

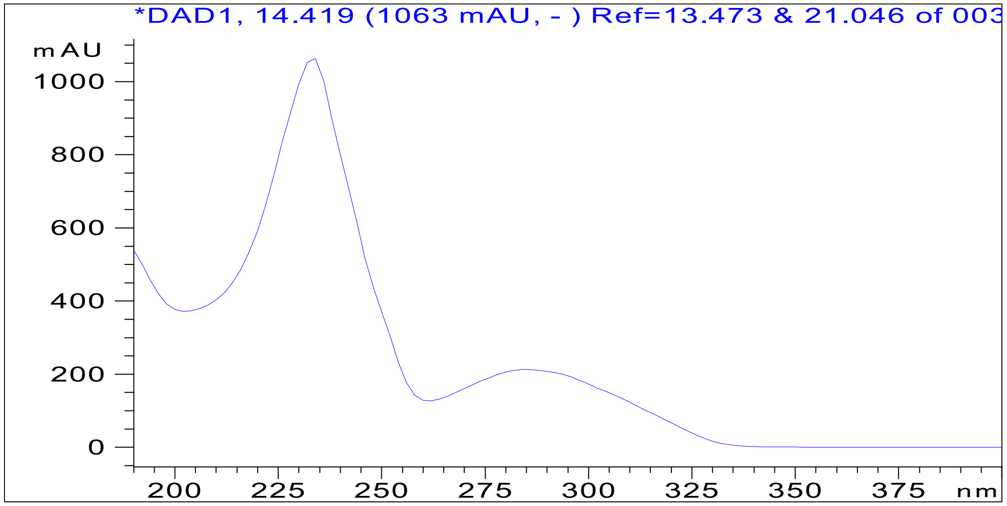

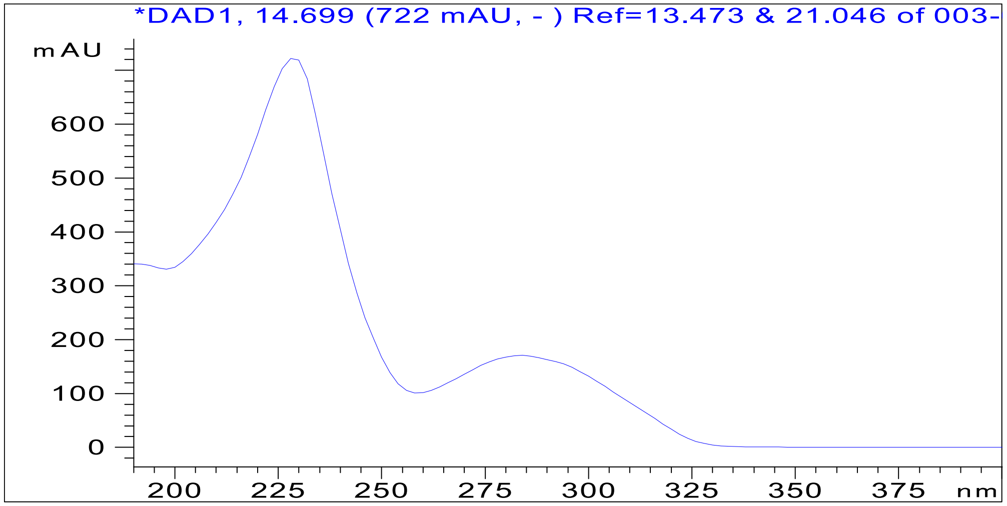

Figure S6k. HPLC-DAD UV spectrum of **R7 6e** HPLC-DAD UV spectrum of **R7 5e**

HPLC-DAD UV spectrum of **R7 4e** HPLC-DAD UV spectrum of **R7 3e**

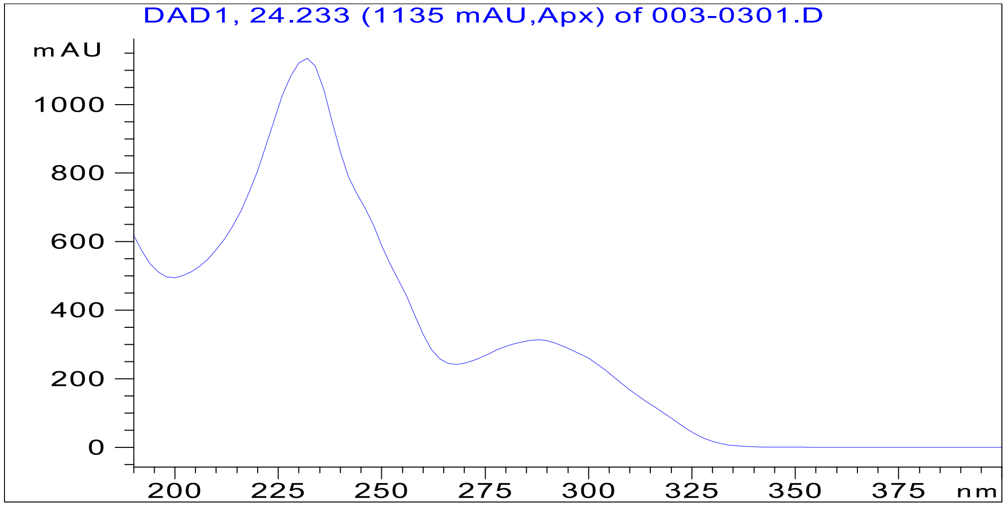

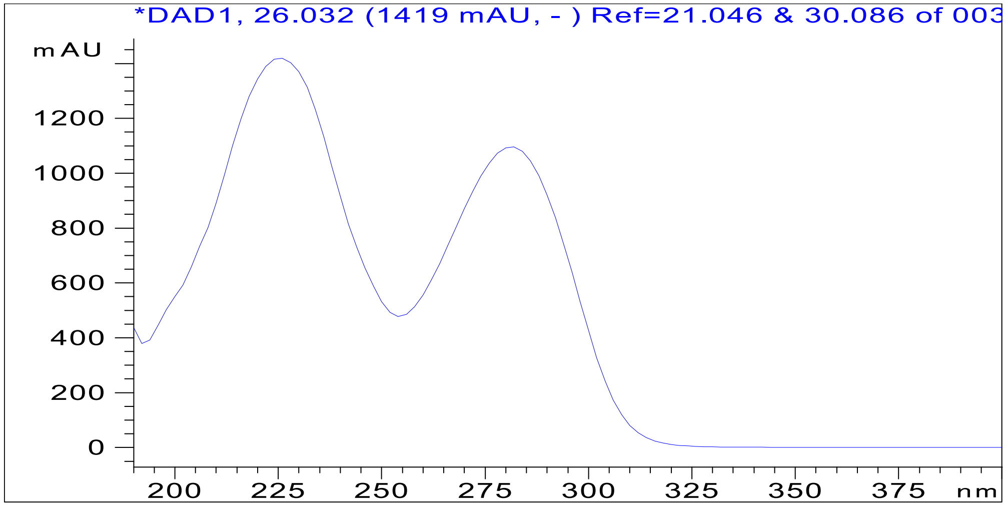

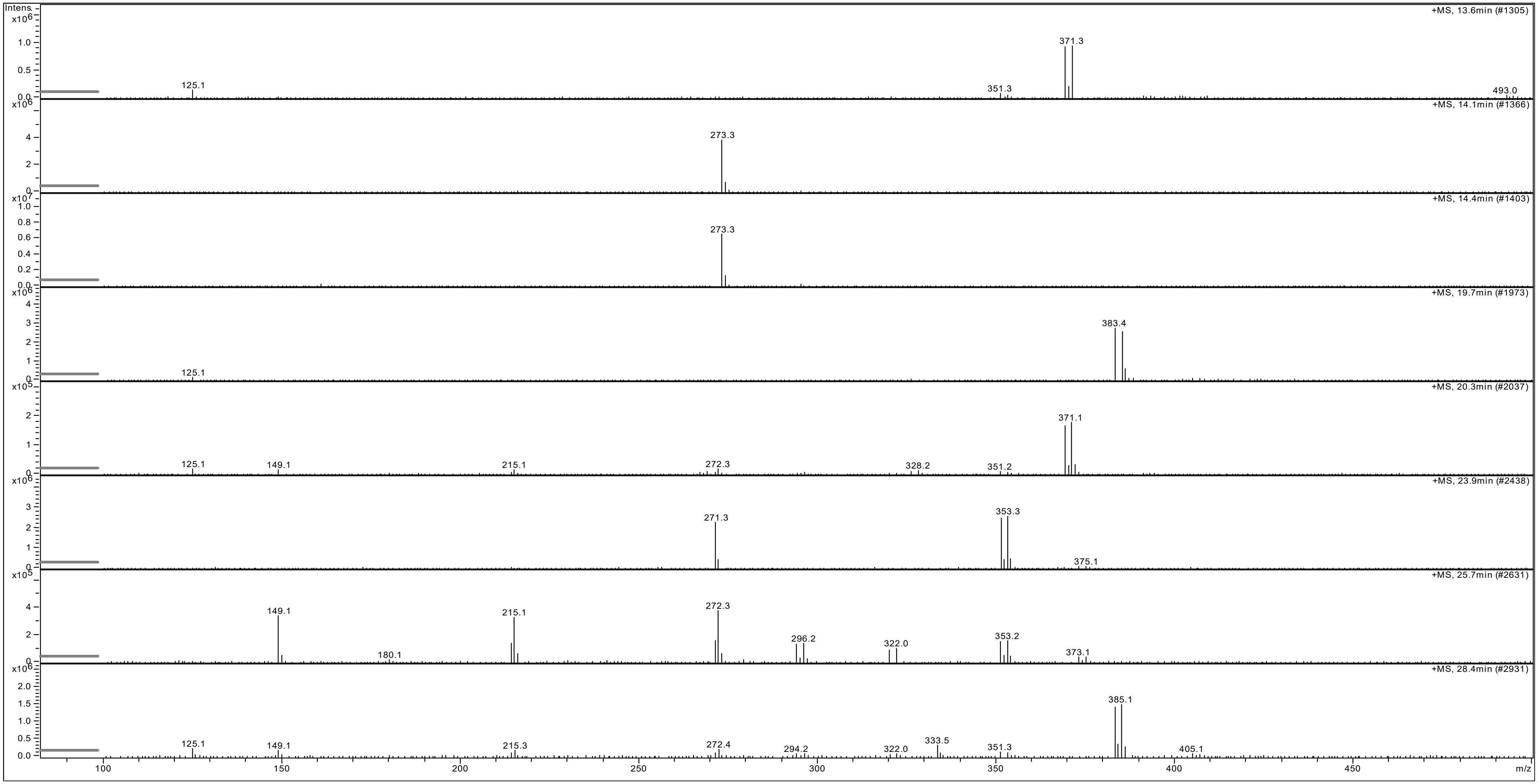

Figure S6l. molecular ions of **R7** at $\mathbf{t_R}$: 13,6 (**1'e**), 14,1 (**6e**), 14,4 (**5e**), 19,7 (**2'e**), 20,3 (**1e**), 23,9 (**4e**), 25,7 (**3e**), 28,4 (**2e**)

Figure S6m. **Comments to foregoing slides:**

– Agelastatin A and five methylated derivatives (**6e**, **1d**, **1m**, **1e**, **2m**, **2e**) were analyzed by HPLC-DAD. All substrates showed very similar UV spectra, having λ_{max} ~ 200, 230, 280 and $\varepsilon_{200} > \varepsilon_{280} > \varepsilon_{230}$ The product **6e** showed UV spectrum having λ_{max} ~ 230, 280 and $\varepsilon_{230} > \varepsilon_{280}$. Subsequently, four reactions were carried out at different conditions as reported in Table 1.

– The chromatograms at λ = 289 are shown for reactions R6, R7, R8. Four aliquots were taken from R9 every 15' before the reaction be quenched after 75'. Noteworthy, the peak at t_R = 23.9 (**4e**) is abundant in R9-1 then slowly disappears, the relative intensity of peaks at t_R = 14.0 (**6e**) and t_R = 14.3 (**5e**) inverts, as well. The peak at t_R = 14.3 (**5e**) is higher in R8 than in other chromatograms. All chromatographic peaks were compared with substrates of known t_R and the new peaks tentatively assigned to products on the base of their ^{1}H-NMR spectra and relative abundance. The examinations of their UV spectra and MS data will give support to these initial assignments.

– In R6, the peak at t_R ~ 20 (**2'e**) showed UV similar to the substrate **2e** (t_R ~ 29). Peak at t_R ~ 24 (**4e**) showed UV similar to product **6e** whereas the peak at t_R ~ 26 (**3e**) showed a different UV spectrum, having λ_{max} ~ 200, 230, 280 and $\varepsilon_{200} > \varepsilon_{230} > \varepsilon_{280}$.

– All peaks in the sample R9-1 showed UV spectra similar to products (**4e**, **5e**, **6e**) which bear the same chromophore. The area of peaks in an ELSD chromatogram generally correspond better than UV chromatograms to the effective amount of each sample: R7 actually contains eight compounds.

– In R7, UV spectra of peaks at t_R = 13,6 (**1'e**), t_R = 19,5 (**2'e**), t_R = 20,3 (**1e**), t_R = 28,4 (**2e**) were similar to substrates; UV spectra of peaks at t_R = 14,1 (**6e**), t_R = 14,4 (**5e**), t_R = 23,9 (**4e**) were similar to products. The peak at t_R = 25,7 (**3e**) confirmed its unique UV bands.

– The (ESI+)-MS measurements on molecular ions confirm the preceding assignments. In particular, the elusive compound eluting at t_R = 23,9 has $[M+H]^+$ at *m/z* = 351-53, as expected for **4e**.

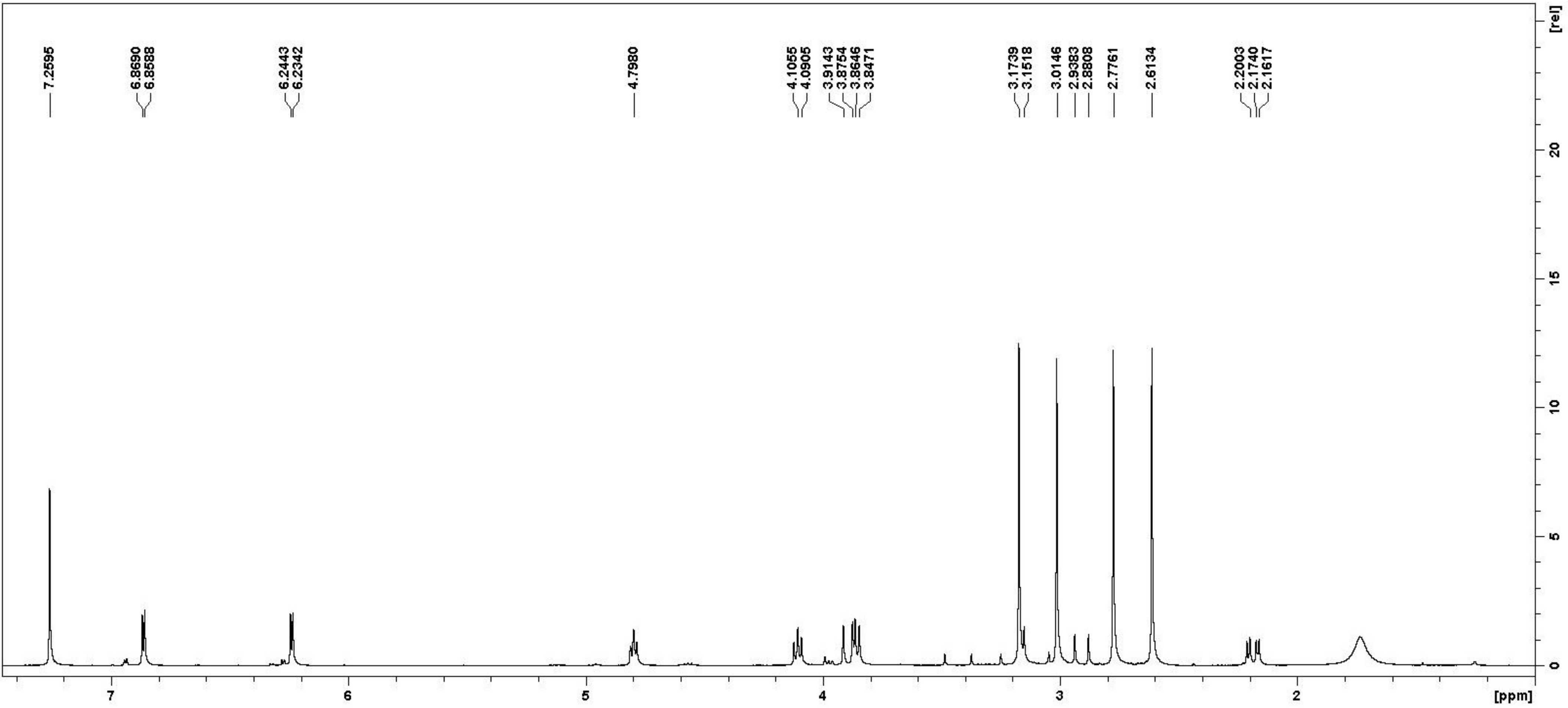

Figure S7a. – pure **2'e**

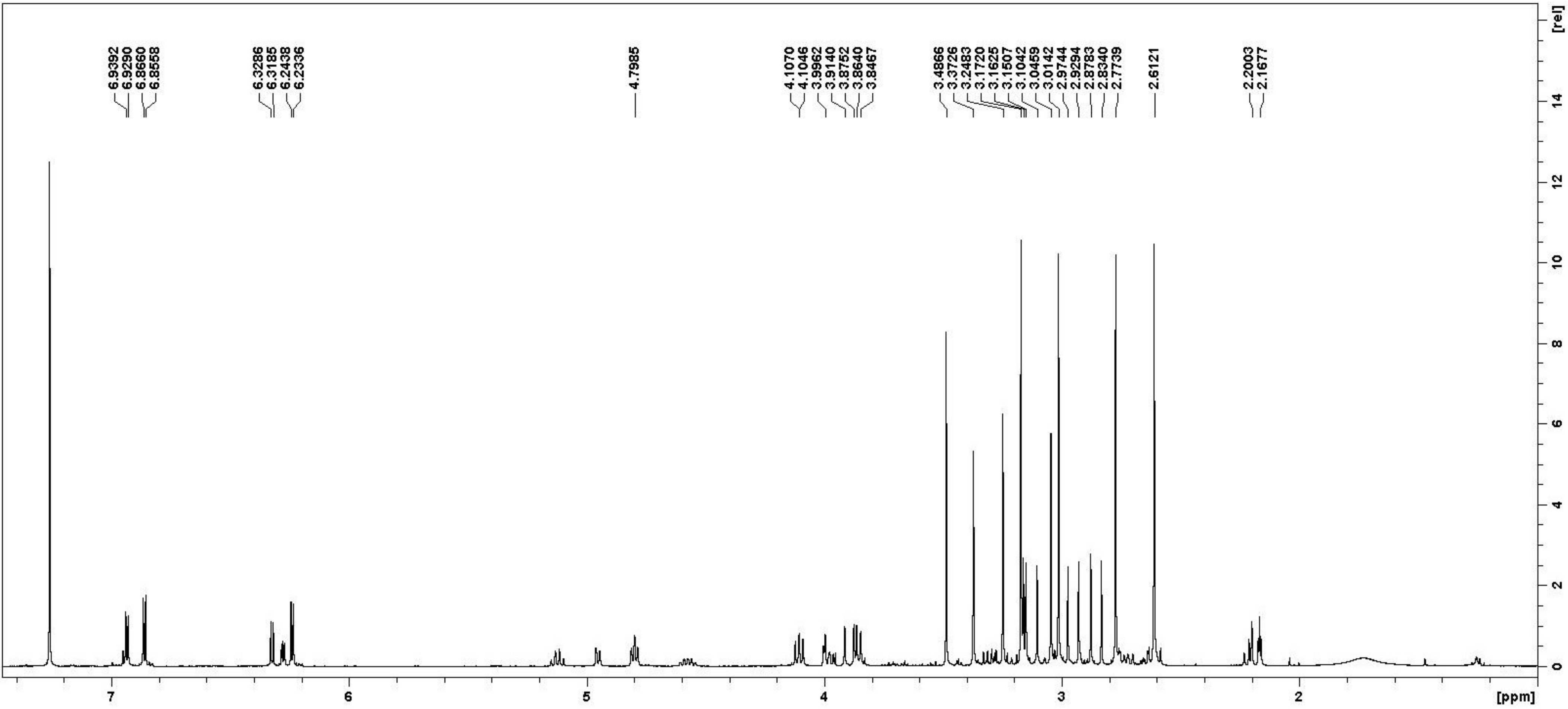

Figure S7b. – pure **2'e** forms a mixture of **2'e**/**3e**/(**1e**+**2e**) ~ 1.5:1.5:(4)

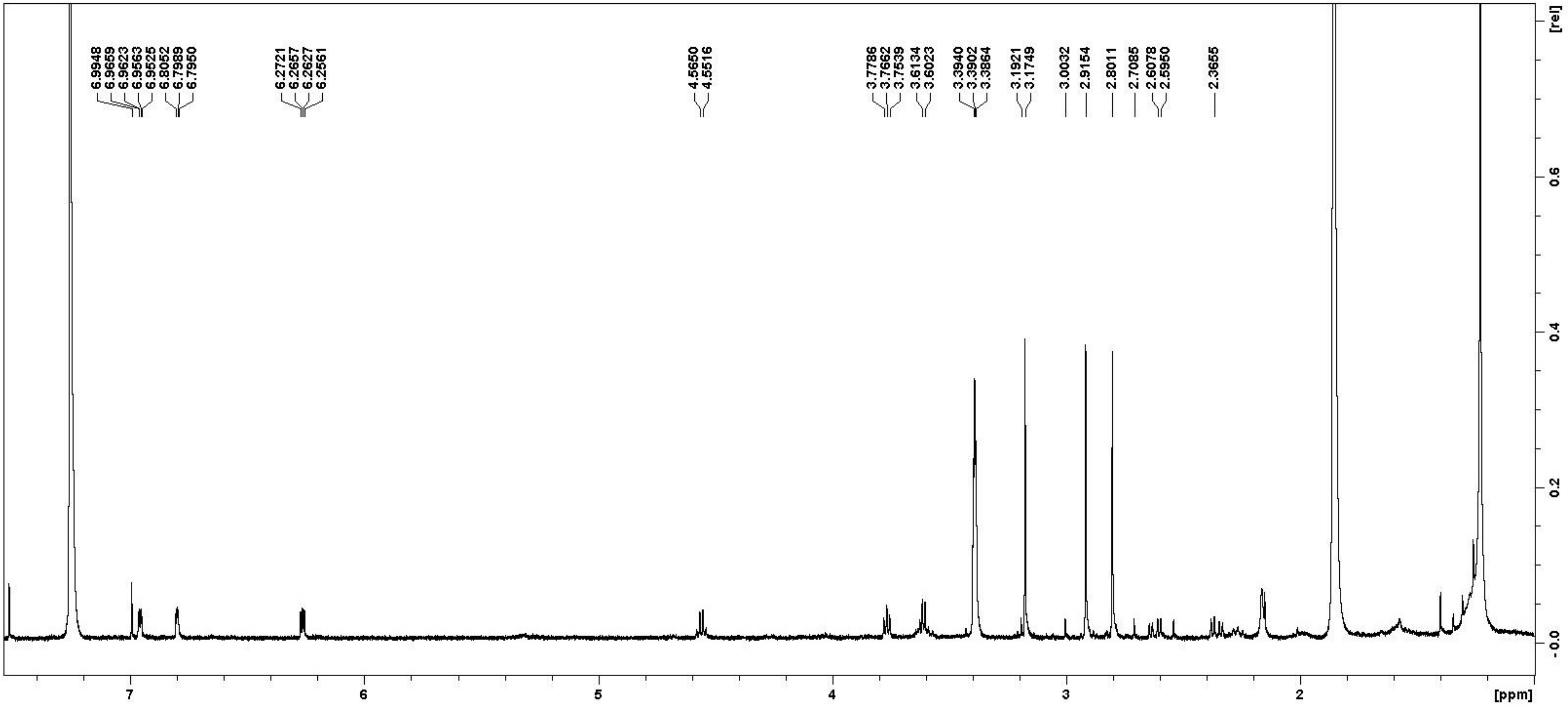

Figure S7c. – **8e** pure

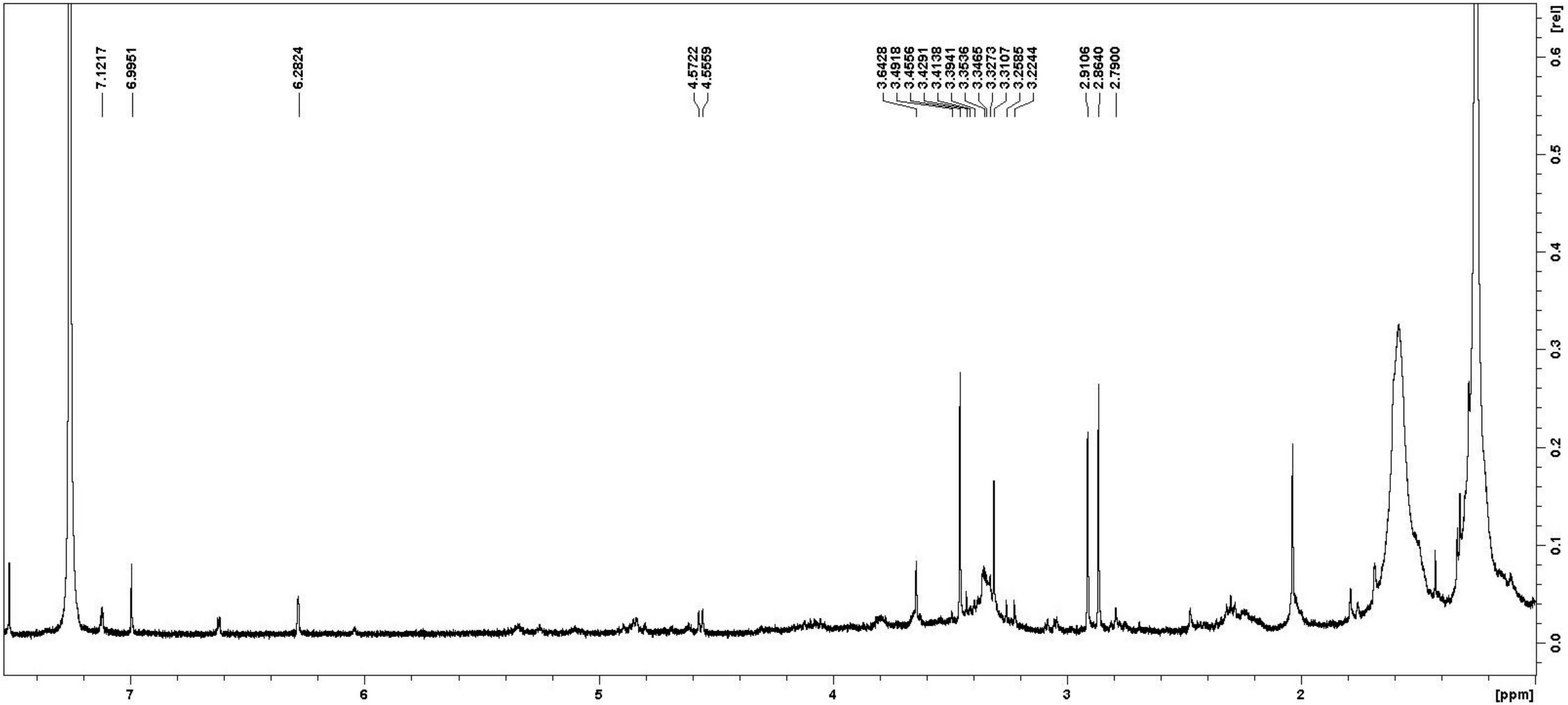

Figure S7d. – **8e** + MsOH 70° (4h + 3h) then A21 => **6e**

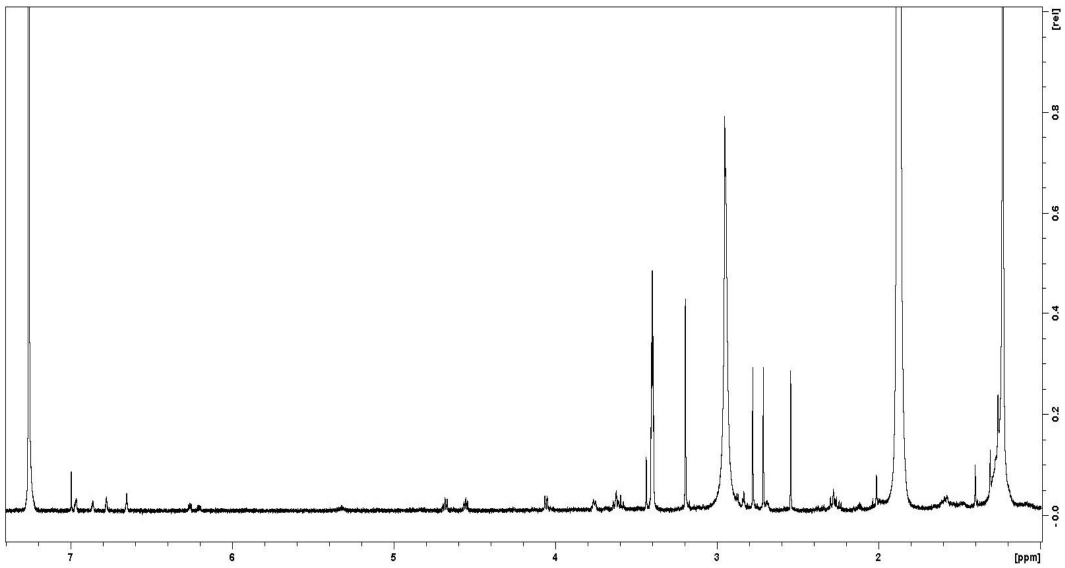

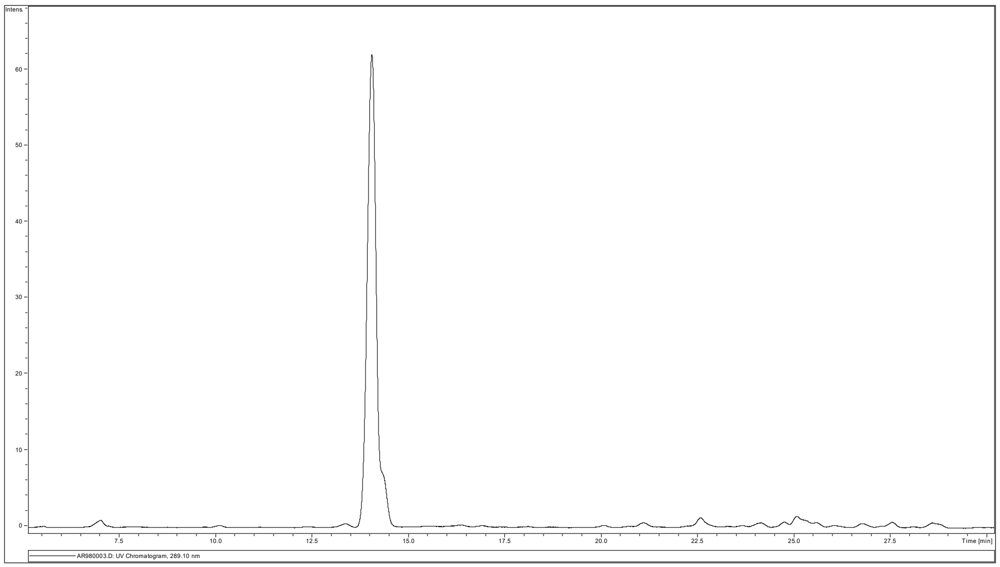

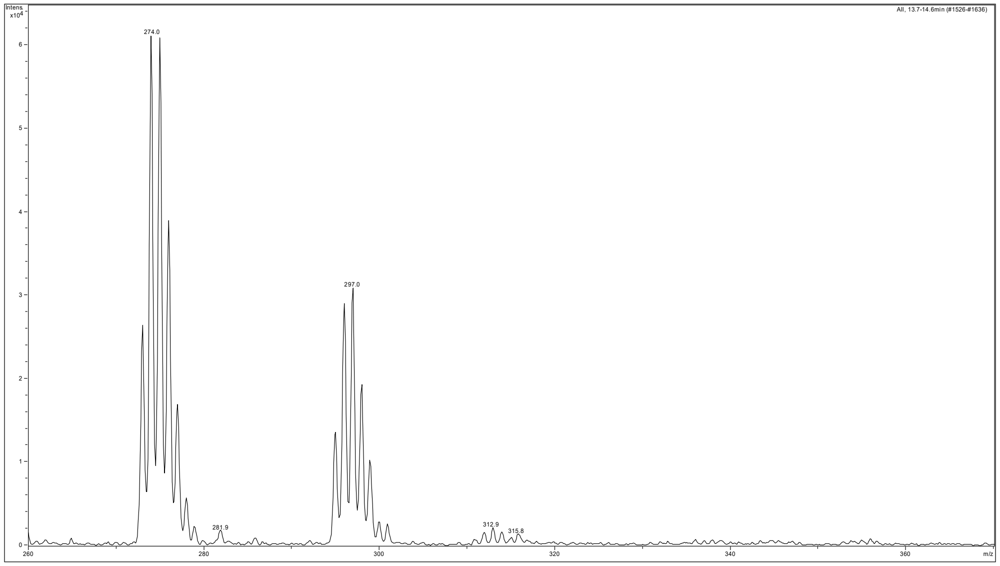

Figure S7e. – **8e** in $CDCl_3$ / CD_3OD 95:5 + MsOH

70° 4h => **8e** / **6e** ~ 50:50

LC chromatogram of raw products after 7h

MS of peak at $\mathbf{t_R}$ ~ 14' (*m/z* = 273)

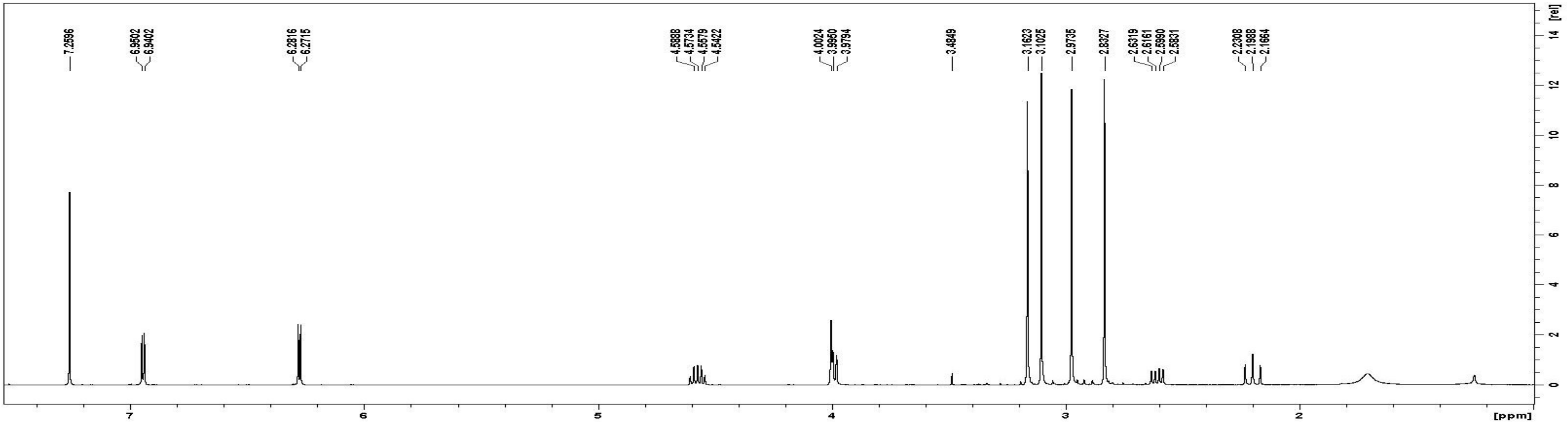

Figure S8a. - ↑ – **2e** for deuterium incorporation ↓ – **5e** for deuterium incorporation

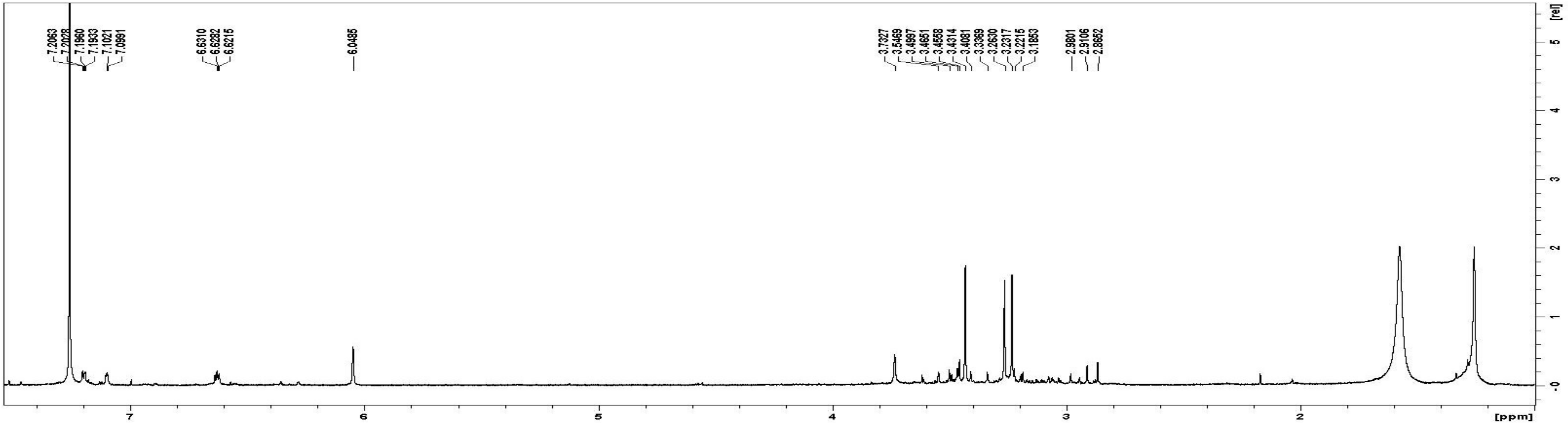

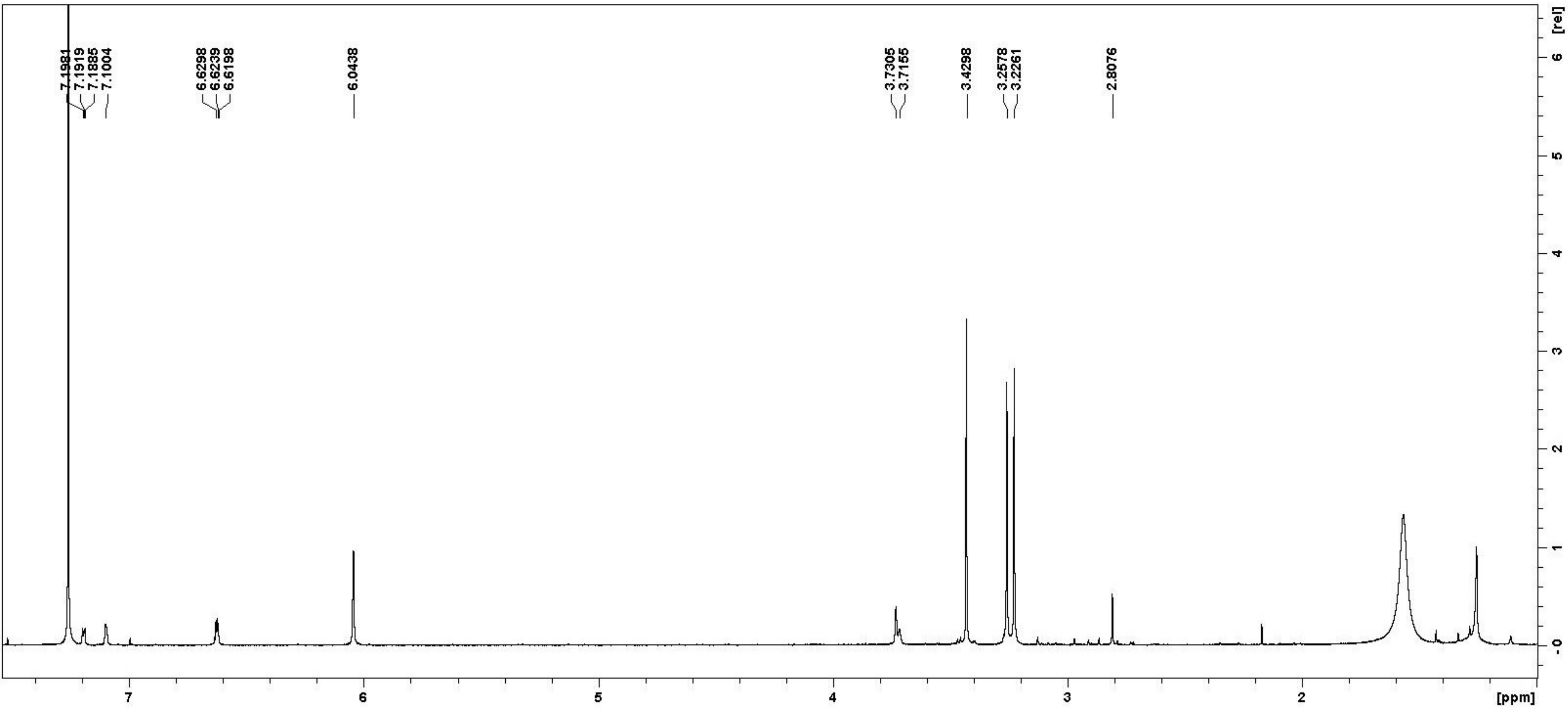

Figure S8b. – **5e**(2e)

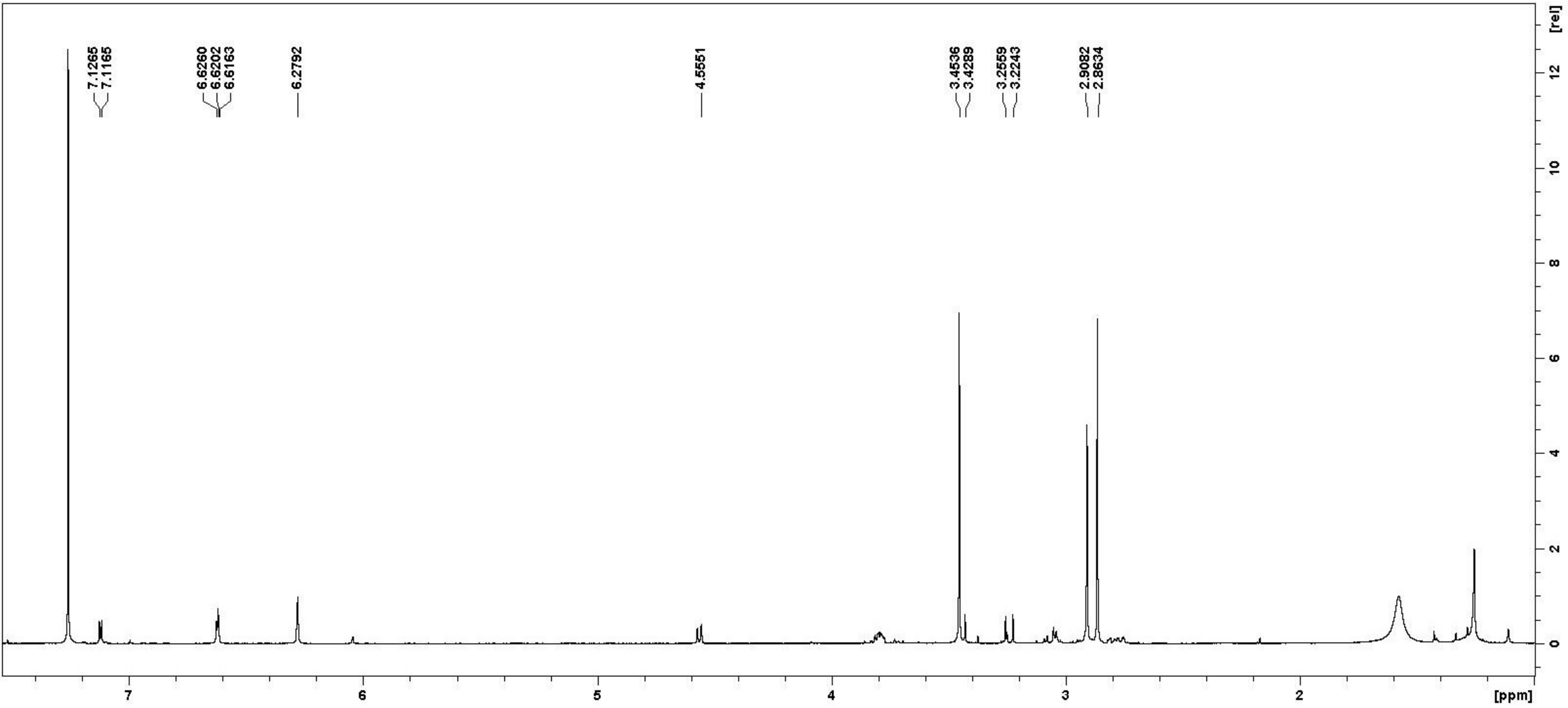

Figure S8c. – **6e**(2e)

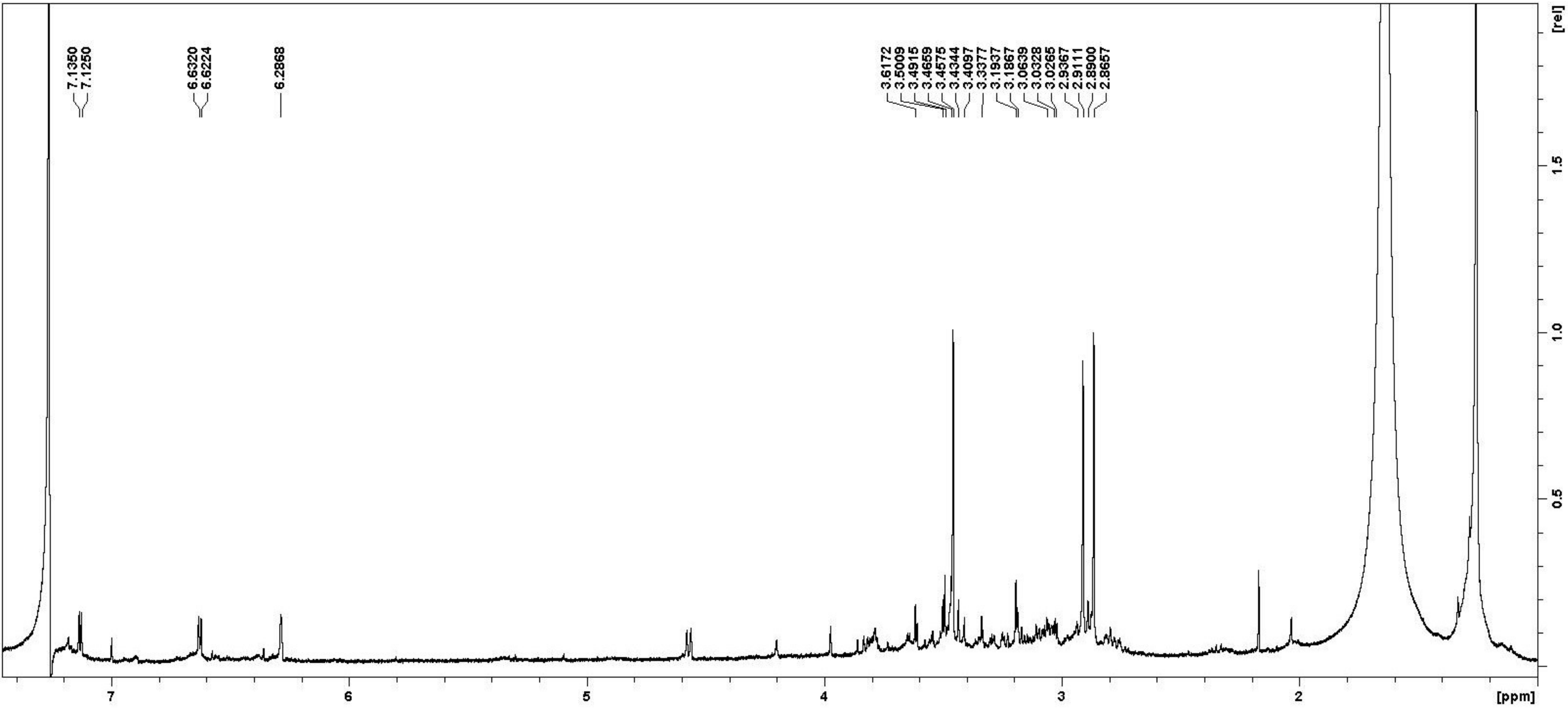

Figure S8d. – **6e**(5e)

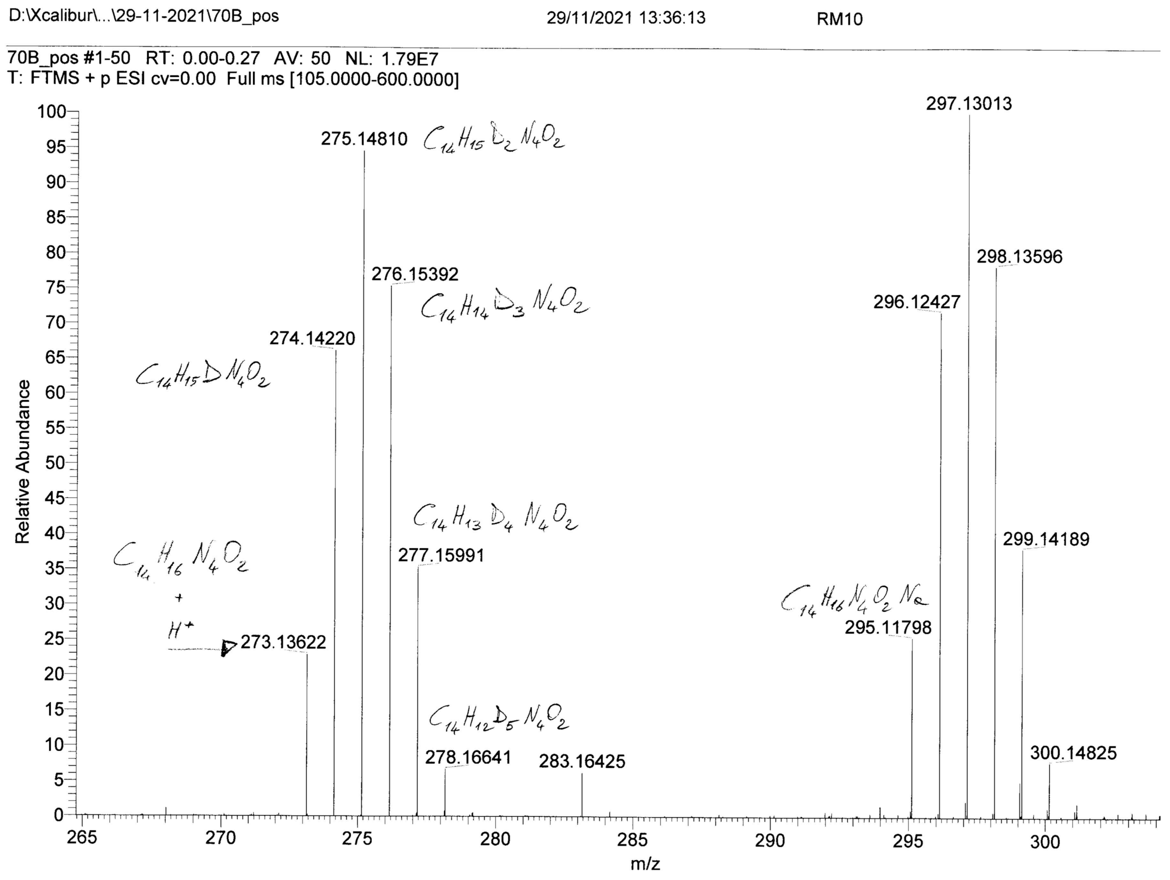

Figure S8e. HRMS of clusters for molecular ion of **6e**(2e)

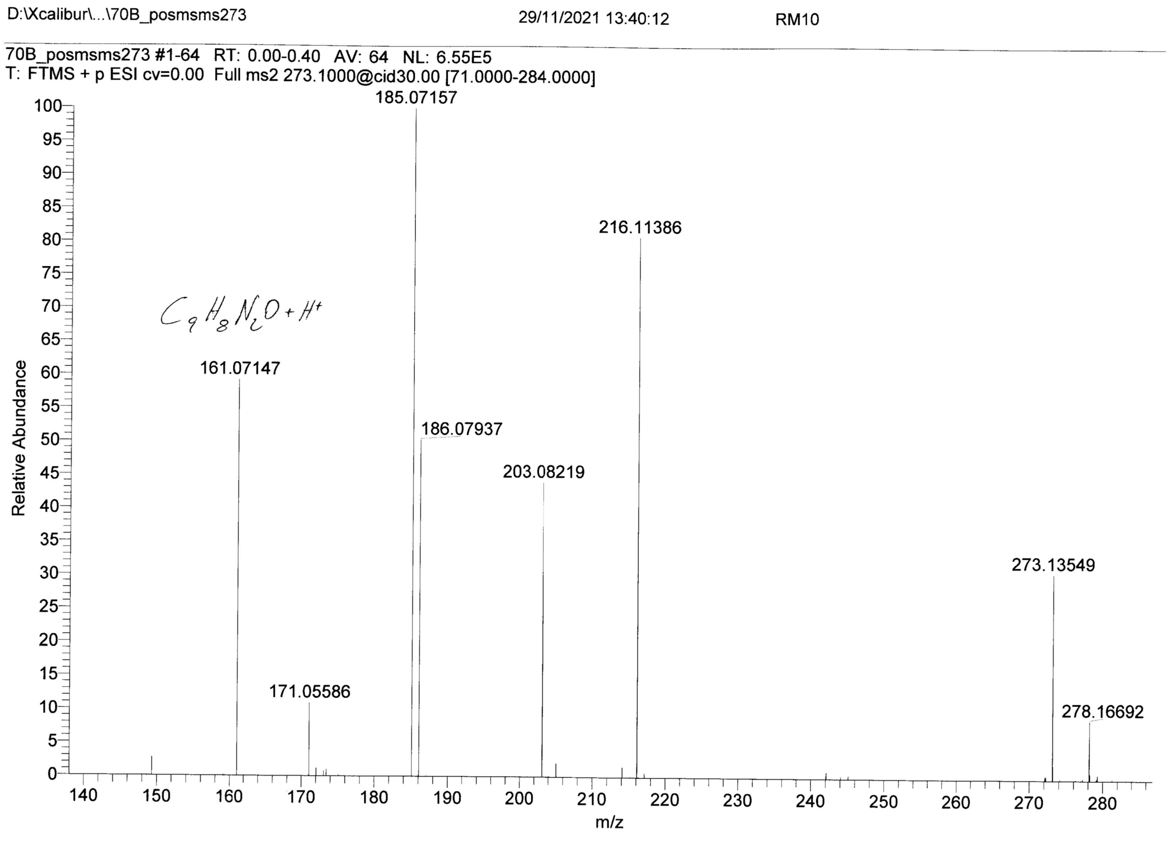

Fig. S8f. HRMS^2 of molecular ion at *m/z* 273 of **6e**(2e)

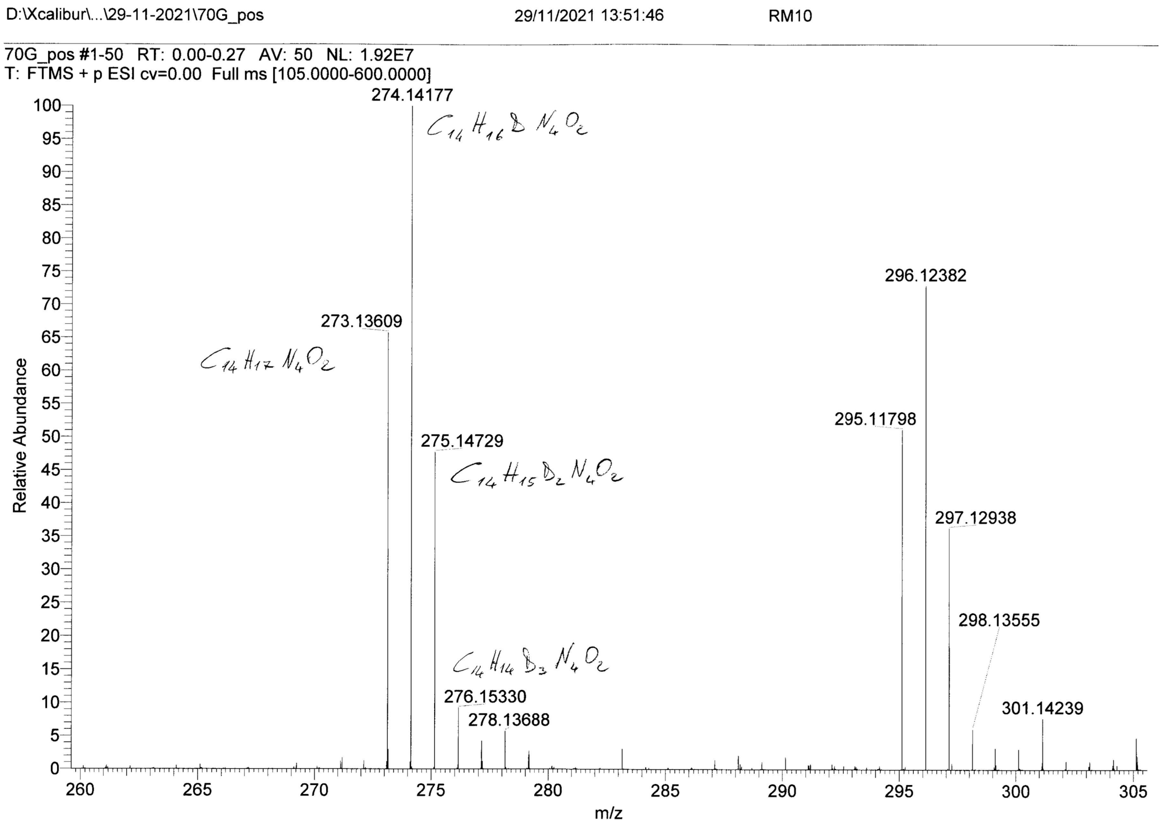

Figure S8g. HRMS of clusters for molecular ion of **5e**(2e)

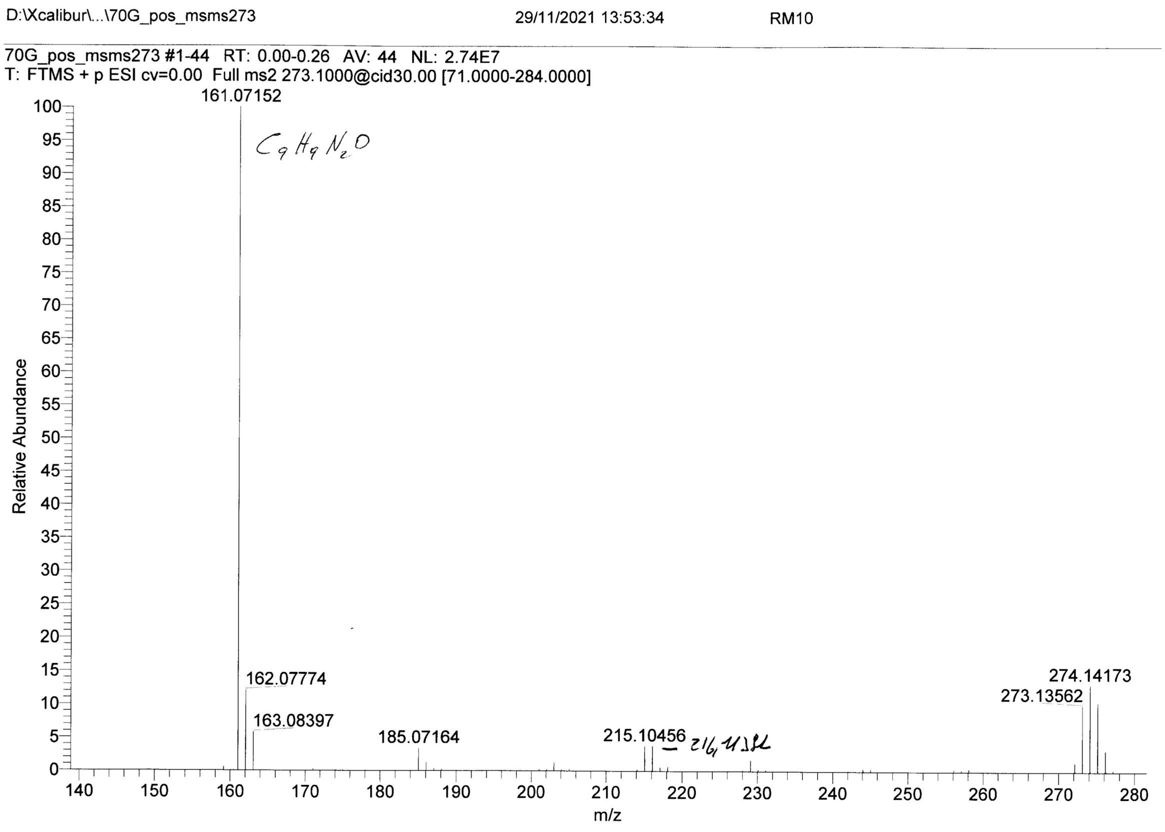

Fig. S8h. HR-MS^2 of molecular ion at *m/z* 273 of **5e**(2e)

A	B	C	D	E	F	G	H	I	J	K	L
Compound	Entry	Formula	$[M+H]^+$ (m/z)	Rel. Int. (%)	D total	$[ion]^+$ (m/z)	Rel. Int. (%)	# D	Neutral loss	# D	**H**/D (%)
6e(2e) Neutral loss % H=51.7% D=48.3%	1	$C_{14}H_{16}N_4O_2$	273	9.3	0	161	100	0	112	0	9.3
	2	$C_{14}H_{15}DN_4O_2$	274	22.4	1	162	78.1	1	112	0	17.5
	3				1	161	21.9	0	113	1	4.9
	4	$C_{14}H_{14}D_2N_4O_2$	275	33.6	2	163	55.2	2	112	0	18.5
	5				2	162	44.8	1	113	1	15.1
	6	$C_{14}H_{14}D_3N_4O_2$	276	20.8	3	164	26.7	3	112	0	5.5
	7				3	163	60.0	2	113	1	12.5
	8				3	162	13.3	1	114	2	2.8
	9	$C_{14}H_{14}D_4N_4O_2$	277	13.9	4	165	6.9	4	112	0	0.9
	10				4	164	58.6	3	113	1	8.1
	11				4	163	34.5	2	114	2	4.8
5e(2e) Neutral loss % H=84.6% D=15.4%	12	$C_{14}H_{16}N_4O_2$	273	24,8	0	161	100	0	112	0	24,8
	13	$C_{14}H_{15}DN_4O_2$	274	45,6	1	162	88,6	1	112	0	40,4
	14				1	161	11,4	0	113	1	5,2
	15	$C_{14}H_{14}D_2N_4O_2$	275	23,5	2	163	70,5	2	112	0	16,5
	16				2	162	29,5	1	113	1	6,9
	17	$C_{14}H_{14}D_3N_4O_2$	276	6,2	3	164	47,7	3	112	0	3,0
	18				3	163	43,1	2	113	1	2,7
	19				3	162	9,2	1	114	2	0,6
6e(5e) Neutral loss % H=78.5% D=21.5%	20	$C_{14}H_{16}N_4O_2$	273	47.0	0	161	100	0	112	0	47.0
	21	$C_{14}H_{15}DN_4O_2$	274	35.6	1	162	71.0	1	112	0	25.3
	22				1	161	29.0	0	113	1	10.3
	23	$C_{14}H_{14}D_2N_4O_2$	275	17.4	2	163	35.7	2	112	0	6.2
	24				2	162	53.6	1	113	1	9.3
	25				2	161	10.7	0	114	2	1.9

Figure S8i.

Table 2

Figure S8j. The cluster of **6e**(2e) was formed by five ions having from zero (*m/z* 273, entry 1) and up to four (*m/z* 274-77, entries 2-11) atoms of deuterium. The respective empirical formulae are reported in col. C, the m/z values in column D, their relative intensity in col. E and the total number of deuterium atoms in col. F. The experiment of MS^2 on the ion $[M+H]^+$ at *m/z* 273 yielded five positive fragments at *m/z* 216, 203, 185, 171, 161 and the full fragmentation pattern is more clearly illustrated in Figure 1.

The fragment ion at *m/z* 161 stands out as a diagnostic ion in that it corresponds to the pyrazinone moiety after loss of the neutral imidazolone moiety. The pyrazinone moiety can contain from zero (*m/z* 161, entry 1) and up to four (*m/z* 162-65, entries 2-11) deuterium atoms. The respective ions are reported in column G, their relative intensity in col. H and the total number of deuterium atoms in col. I. Likely, the deuteration occurs easily at C14 and C15 by a SEA reaction, which can take place at any stage of the transformation from **2e** to **6e**, included. An acid catalysed interconversion between **2e** and an alleged olefin **9e**, involved in the equilibrium carbocation **A** ⇆ **9e**, would explain the presence of four deuterium in the pyrazinone moiety (col. I, entry 9) . The compound **9e** has not be isolated in the present study but an agelastatin derivative with C5=C6 double bond has been previously reported. The difference between the *m/z* of each molecular ion (col. D) and the corresponding pyrazinone ion (col. G) constitutes the mass value of each neutral loss, which account for the imidazolone moiety. The respective mass values are reported in column J, their total number of deuterium atoms in col. K and the H/D relative abundance in col. L. The deuterium can be linked at either C4 and/or C5 of the imidazolone ring. The deuteration at C4 occurs because of the equilibrium carbocation **A** ⇆ **3e** whereas the deuteration at C5 is the starting point\step from **5e** to **6e**.

The mass spectrometry data for compounds **5e**(2e) and **6e**(5e) were processed by applying the same calculations used for **6e**(2e) . The cluster of **5e**(2e) showed four ions having from zero (*m/z* 273, entry 12) and up to three (*m/z* 274-76, entries 13-19) atoms of deuterium.

Figure S8j.
The fragmentation pattern of **5e** at *m/z* 273 consisted only of a very week fragment at *m/z* 216 and an intense one at *m/z* 161. The pyrazinone moiety could accommodate up to three deuterium at the pyrrole ring (*m/z* 164) .
The loss of 114 Dalton (col. J, entry 19) implies the presence of two deuterium at the imidazolone portion. The deuterium at C4 comes from the equilibrium A ⇆ 3 but the simultaneous presence of deuterium at C5 force to write 5 ⇆ C as equilibrium instead of 5 → C.
The cluster of **6e**(5e) showed just three peaks at *m/z* 273, 274, 275 (entries 20-25) because the deuteration at C6 is no longer possible. The loss of 114 Dalton (col. J, entry 25) is also\ still observed by fragmentation of the ion $[M+H]^+$ at *m/z* 275. The reaction steps 5 ⇆ C ⇆ D → 6 explain the deuterium at C5 but the simultaneous presence of deuterium at C4 force the introduction of an equilibrium 5 ⇆ E. Obviously, it is not possible to establish the position of deuteration in the neutral loss of 113 Dalton mass value. What matters is that the total percentage of deuterated imidazolone losses (col. A) amounts to about 48% in **6e**(2e) , 15% in **5e**(2e) and 21% in **6e**(5e) . The percentage difference **6e**(2e) – **5e**(2e) = 33% can be ascribed to the deuteration at C5 for the final steps of conversion **5e** → **6e**. The value 33% is double of 15% value deriving from the equilibria A ⇆ 3, 5 ⇆ C, 5 ⇆ E and therefore can be considered a valid \ effective support of the mechanism illustrated in the Scheme. The percentage of 21% deuteration on the imidazolone of **6e**(5e) comes from an experiment performed under conditions other than **6e**(2e) and does not differ much from 33%. In my opinion, this deuteration comes in minimal part from the equilibria **5 ⇆ C**, **5 ⇆ E** (missing **A ⇆ 3**) but mostly from the steps **5e** ⇆ C ⇆ D → **6e**. The medium-low incorporation of deuterium might arise from poor drying of reaction flasks.

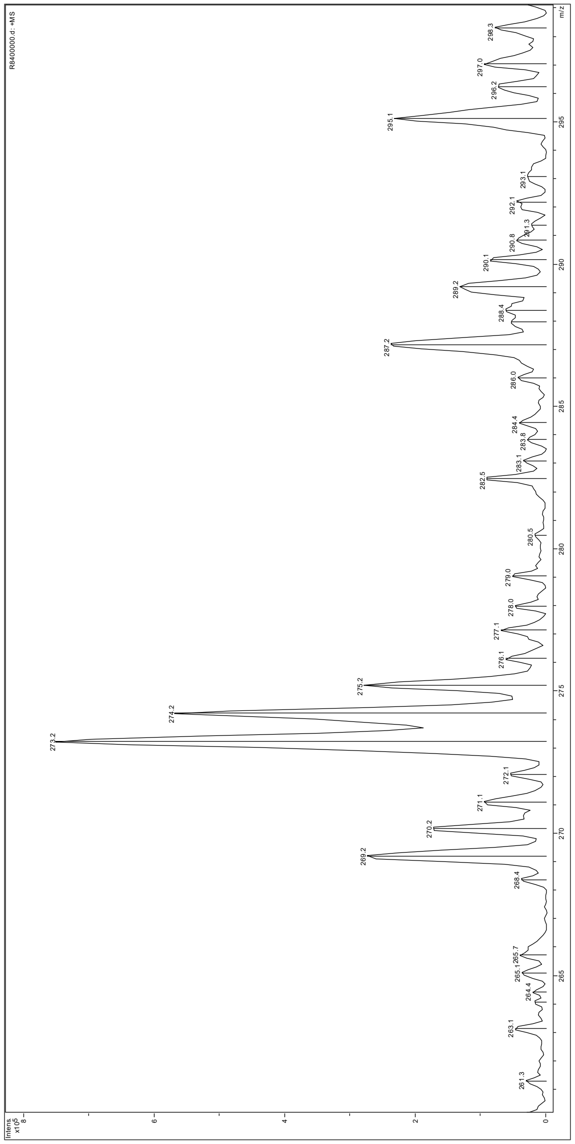

Fig. S8k. 84 Molecular ions of **6e**(5e)

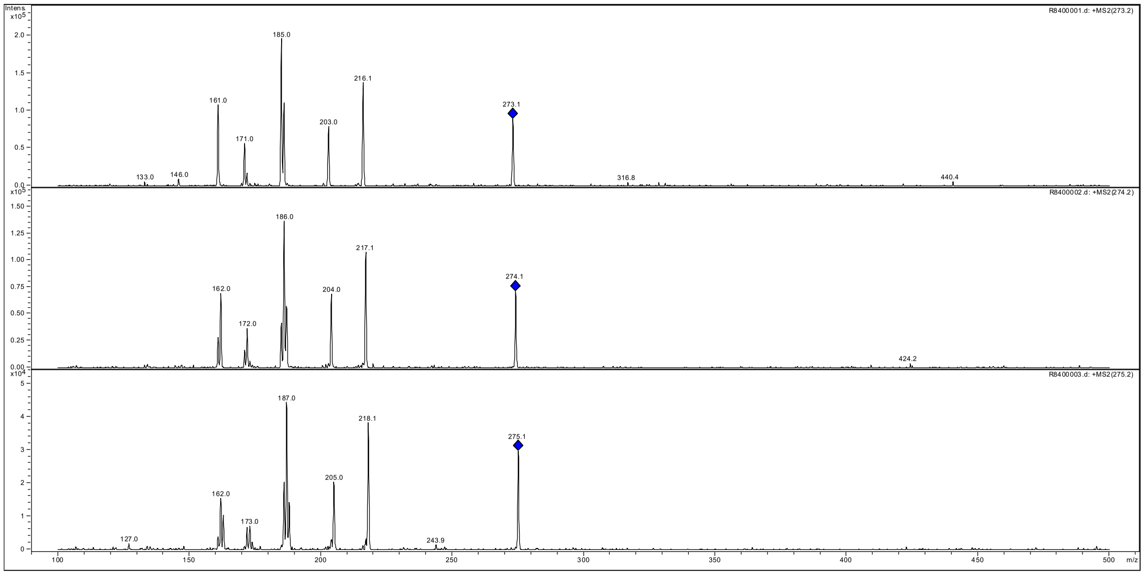

Figure S8l. MS^2 of molecular ions of **6e**(5e)

Figure S8m. Molecular ions and MS^2 at Molecular ions of **5e**(2e)

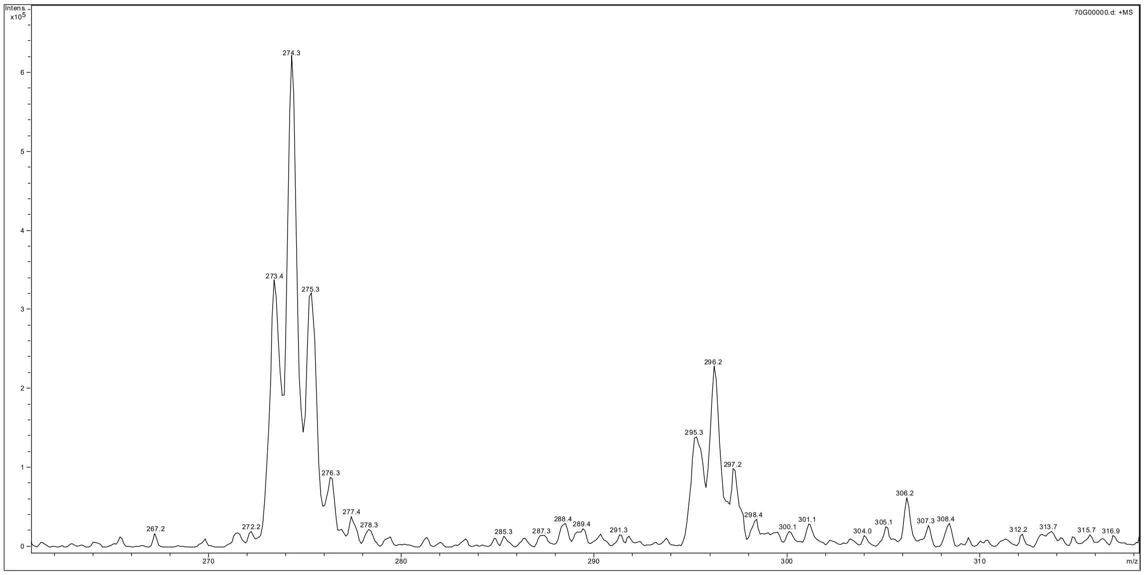

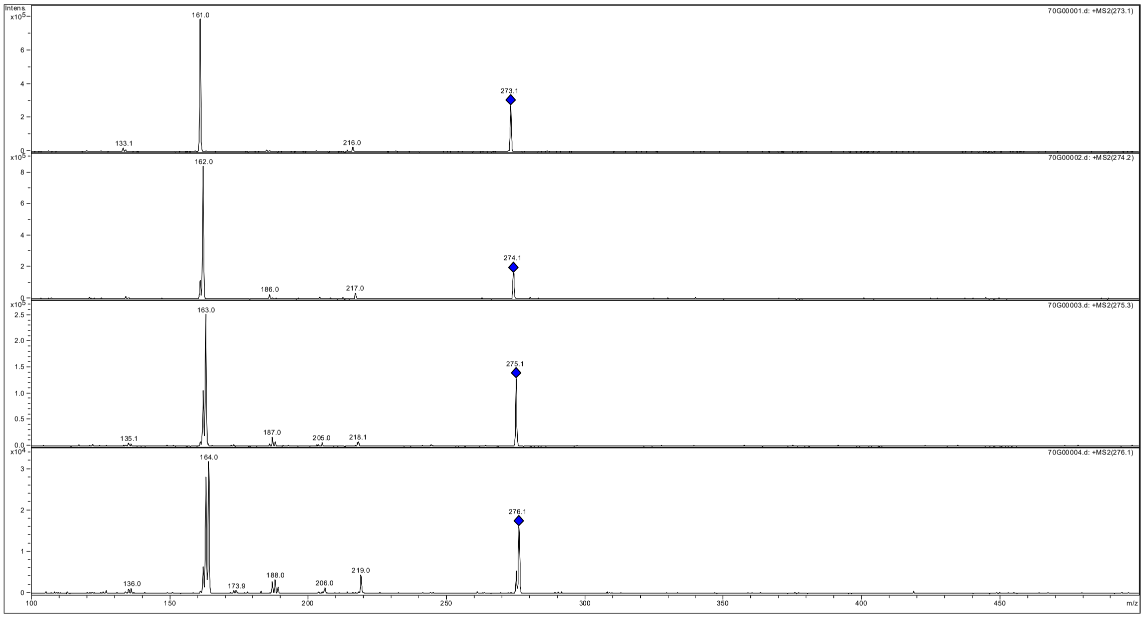

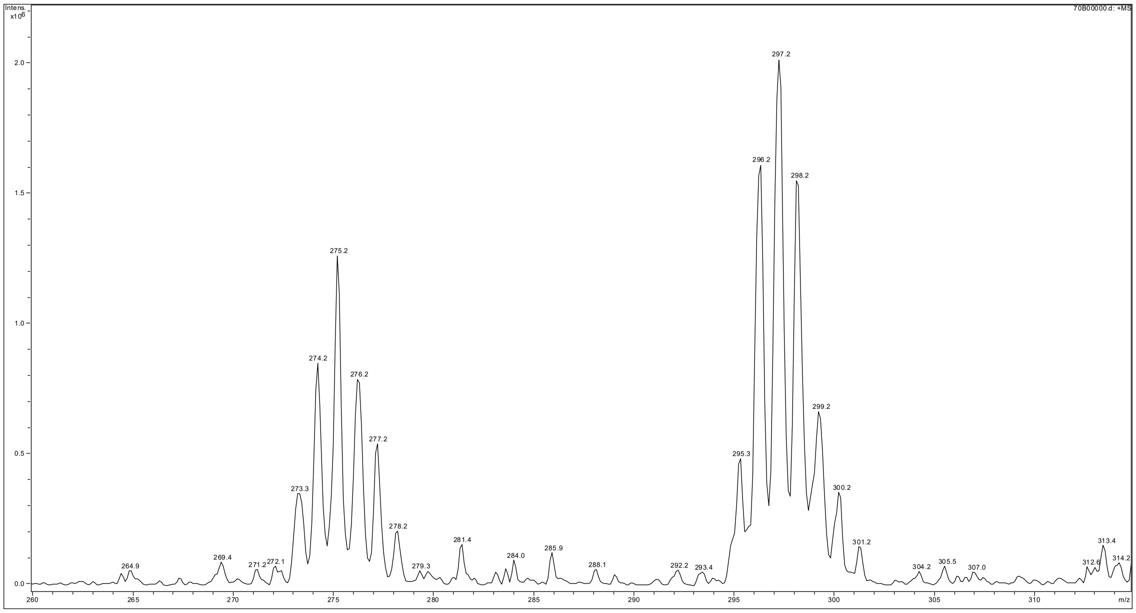

Figure S8n. Molecular ions and MS^2 at Molecular ions of **6e**(2e)

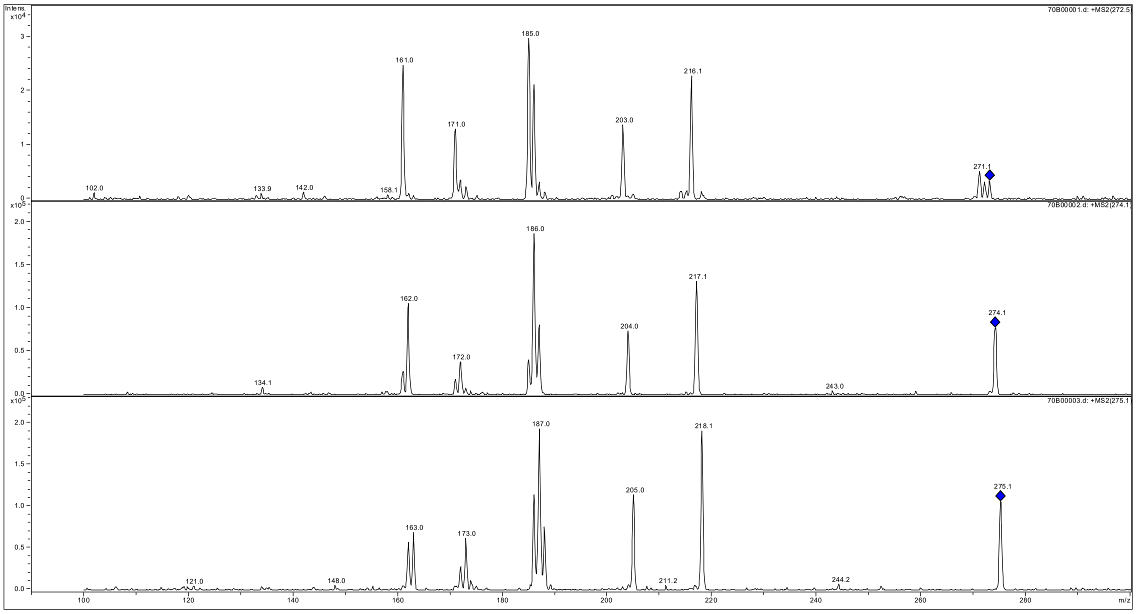

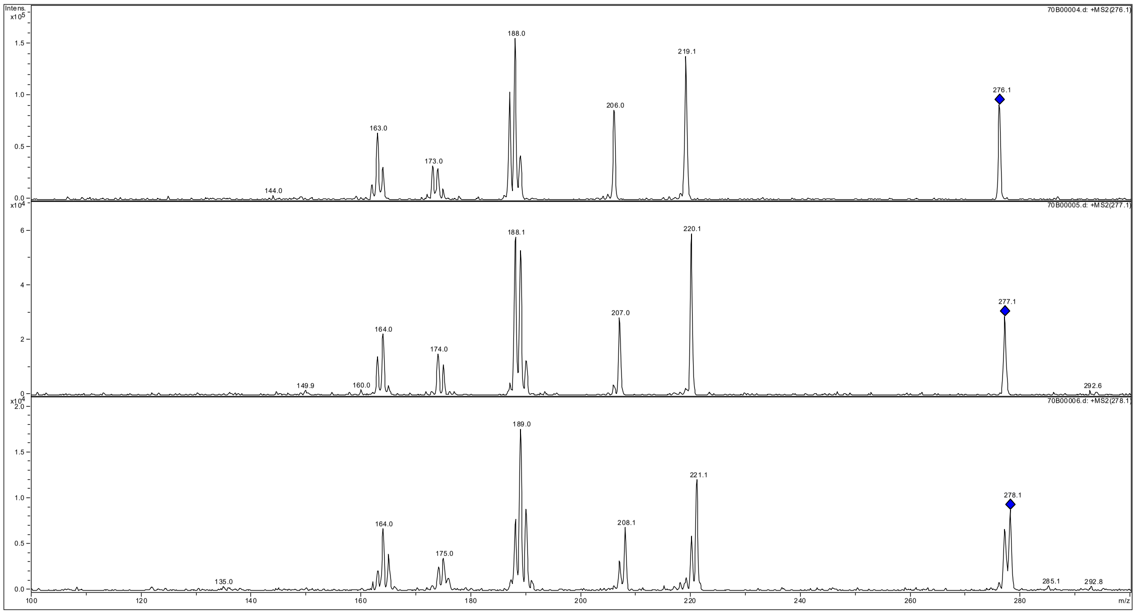

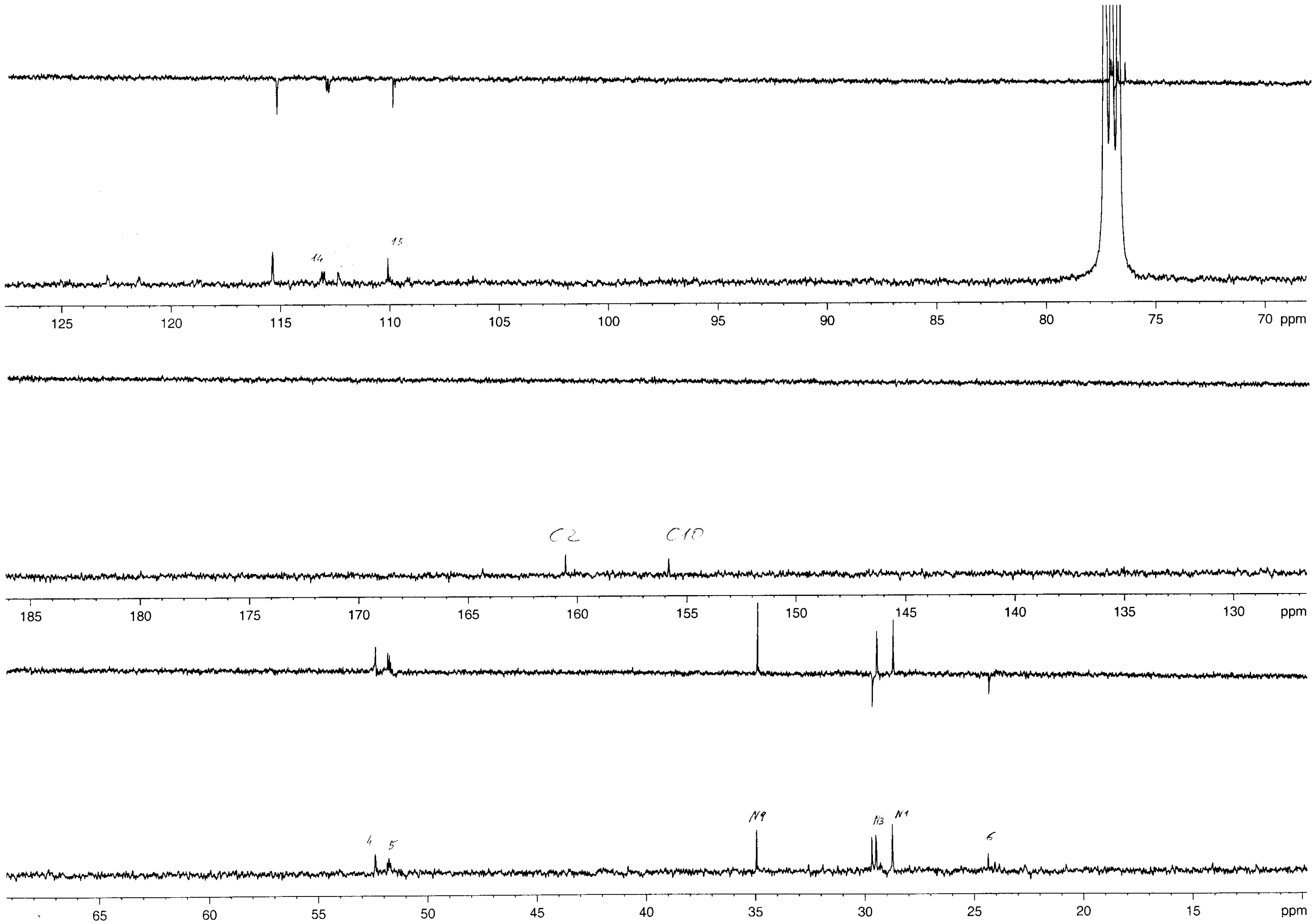

Figure S8o. Three levels plot of ^{13}C-NMR and DEPT spectra of partially deuterated **6e**(2e). NB. Automated processing failed the proper phasing of DEPT-135.

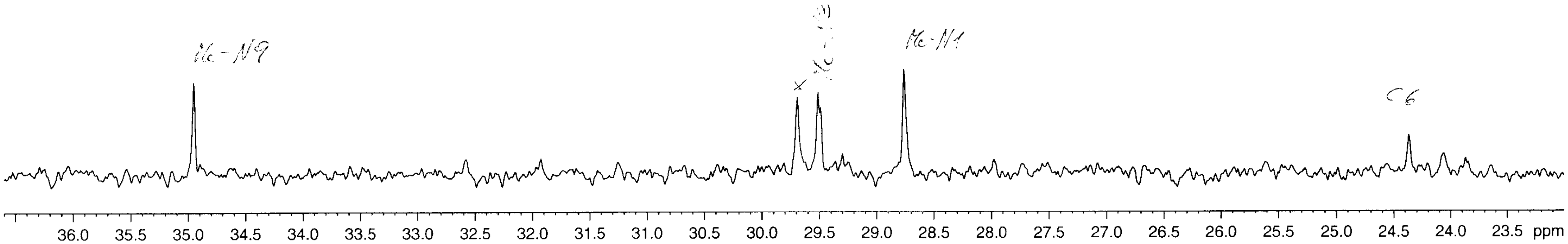

Figure S8p.

Enlarged plot of ^{13}C-NMR spectrum of partially deuterated **6e**(2e)

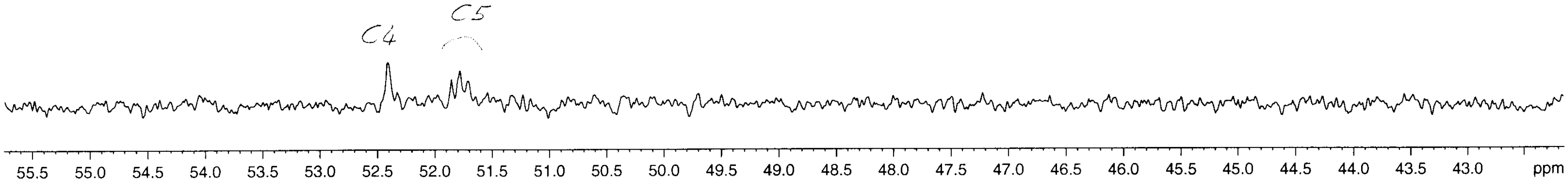

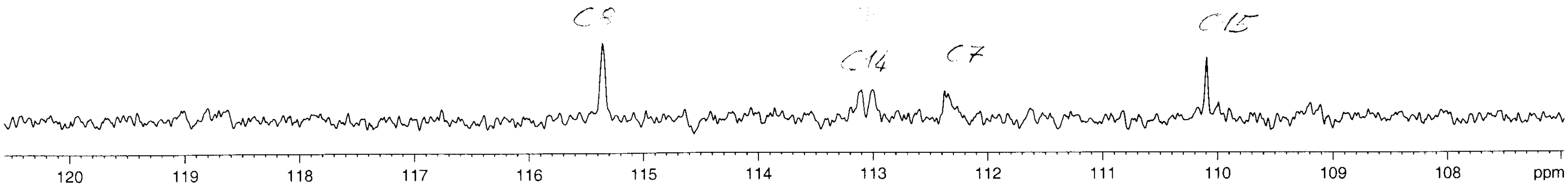

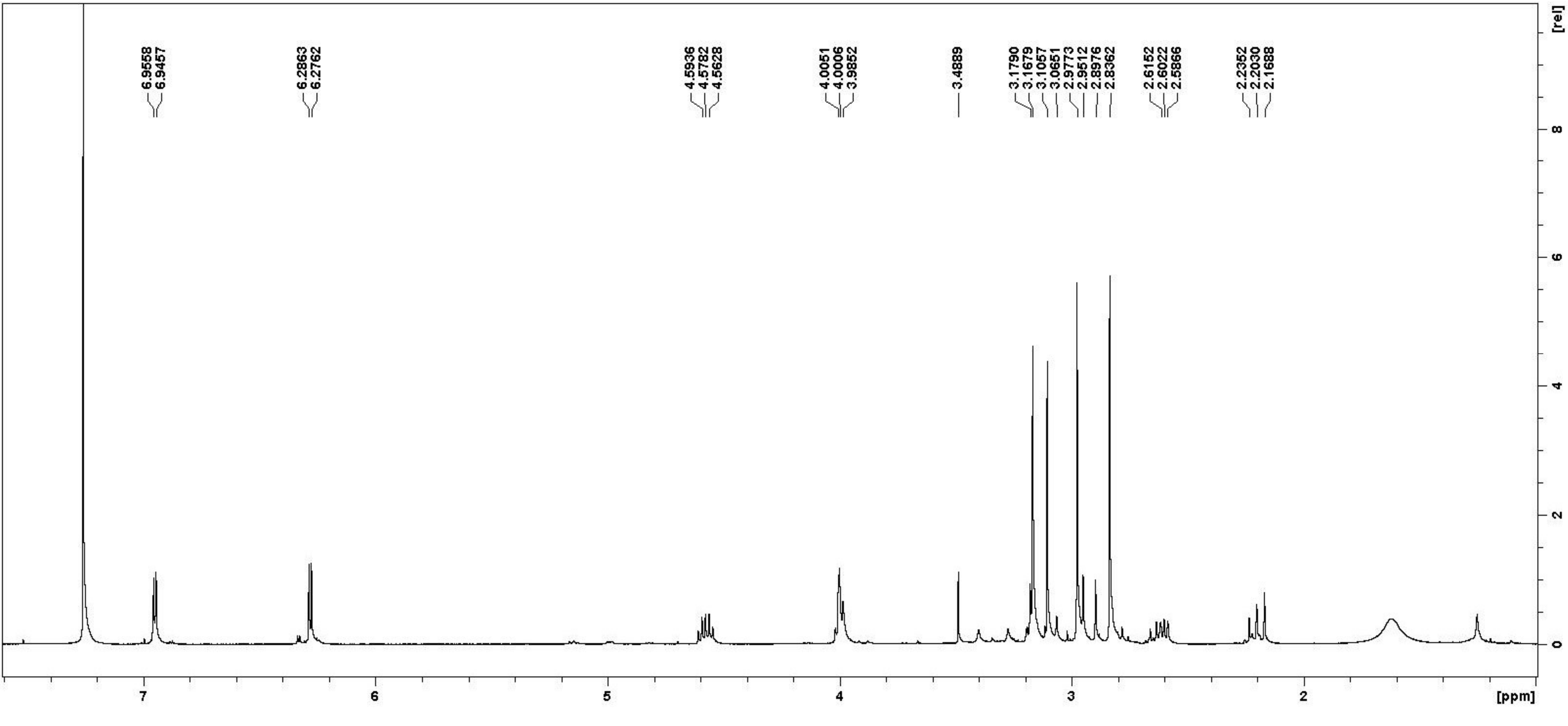

Figure S9a. 2e for reaction performed in the NMR tube

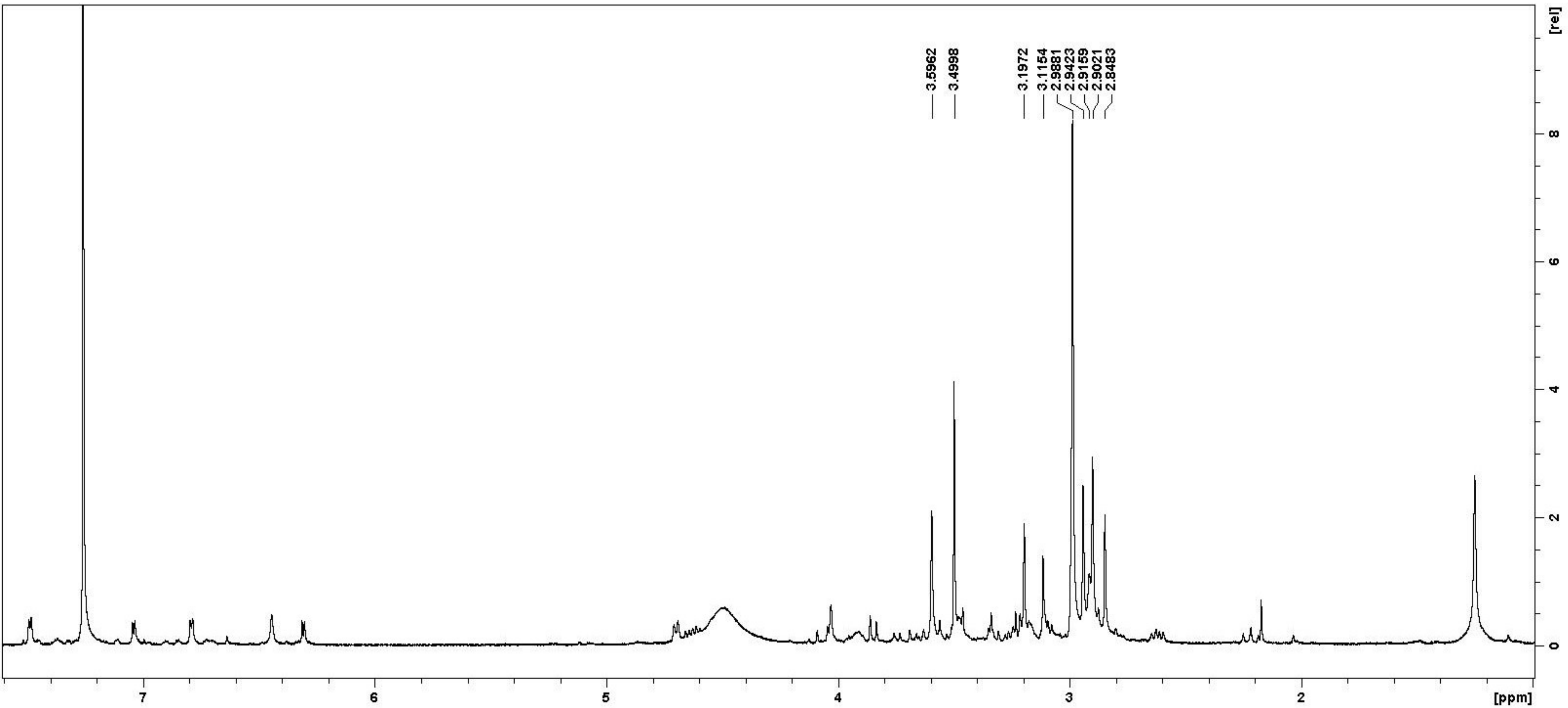

Figure S9b. – **2e** + MsOH in CDCl3 65°, 30’ = **2e** \ **6e** 50:50

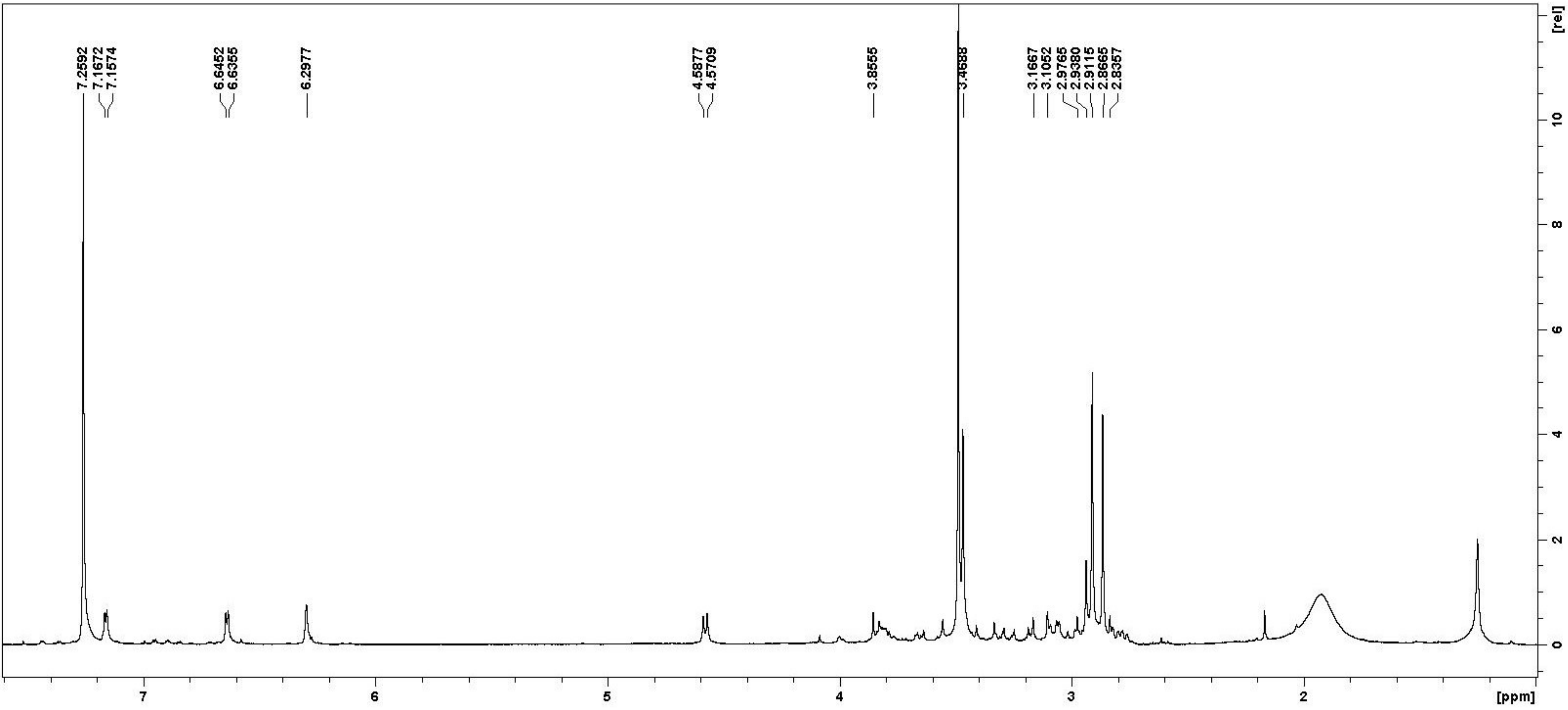

Figure S9c. – **2e** + MsOH in CDCl3 65°, 60' then A21 **6e**

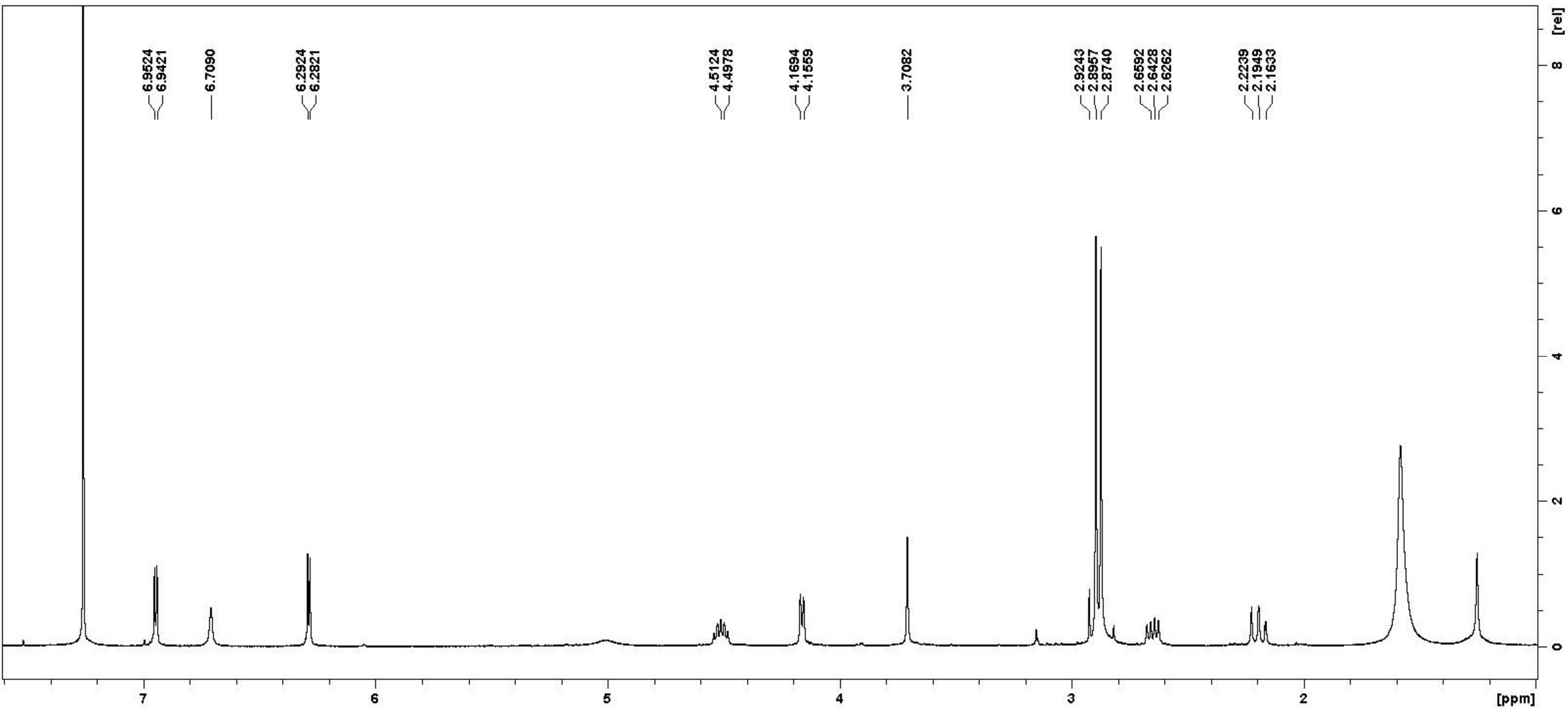

Figure S9d. – **1m** for reaction performed in the NMR tube

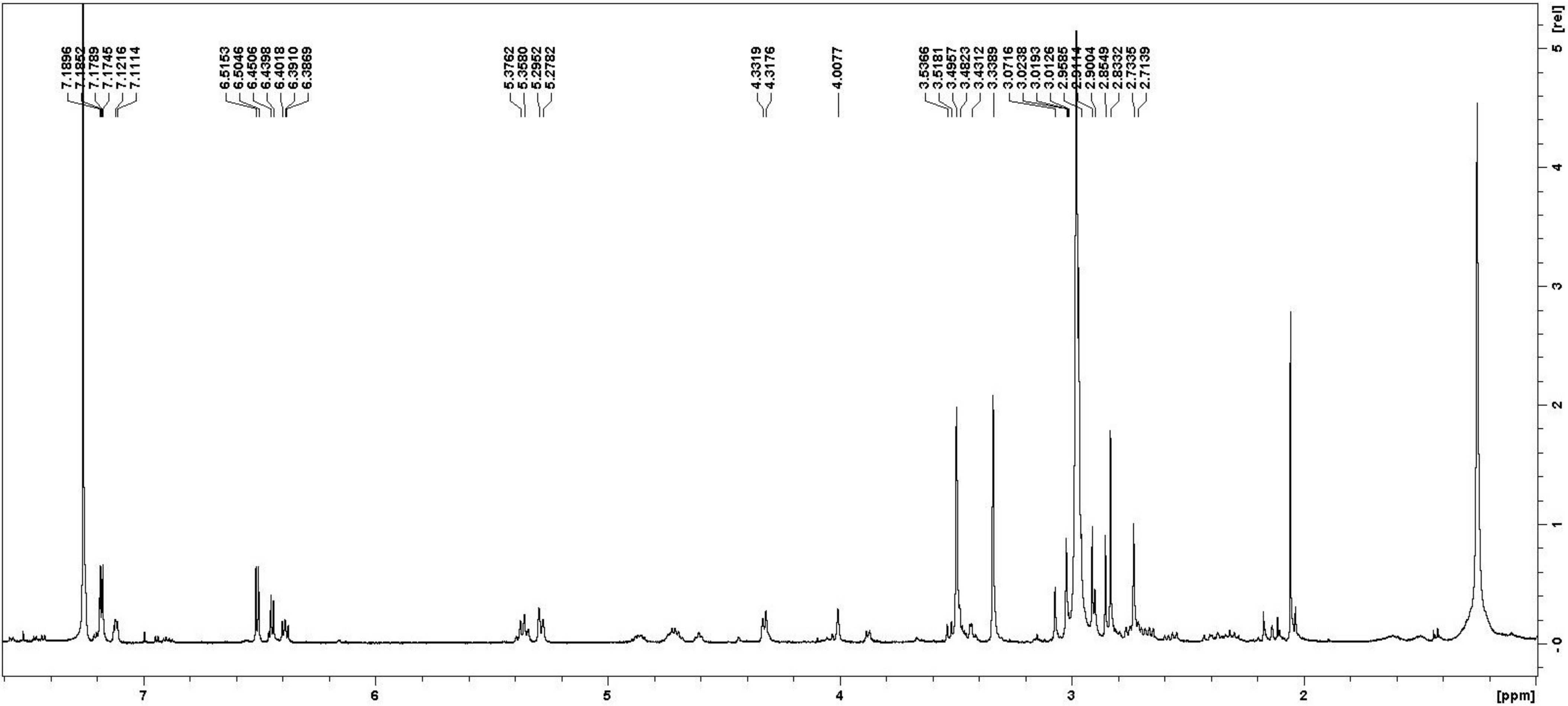

Figure S9e. – **1m** + MsOH in CDCl3 65°,430' => **1m** + **3m**

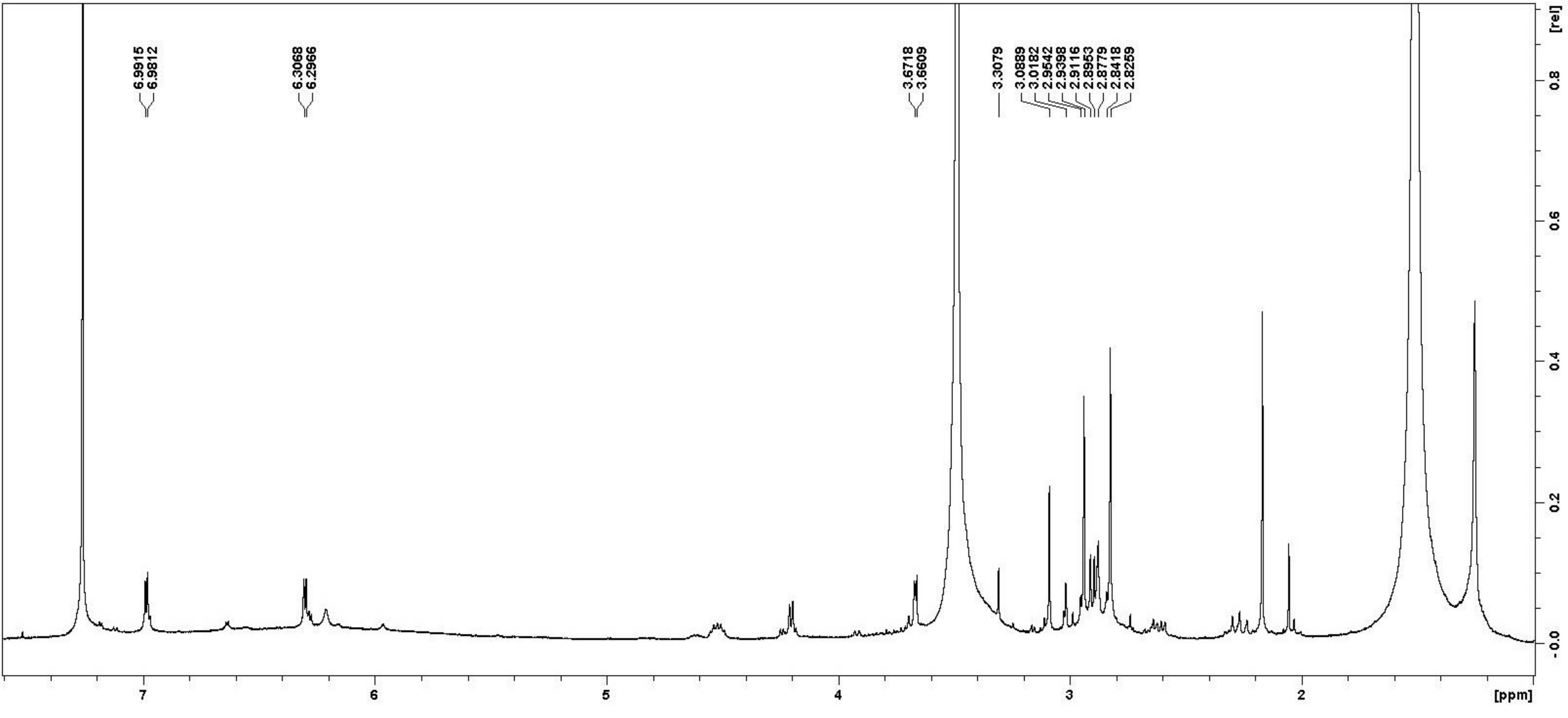

Figure S9f. – **1m** + MsOH in CDCl3 65° ,430’ , 1 day r.t. than A21 => **1m**

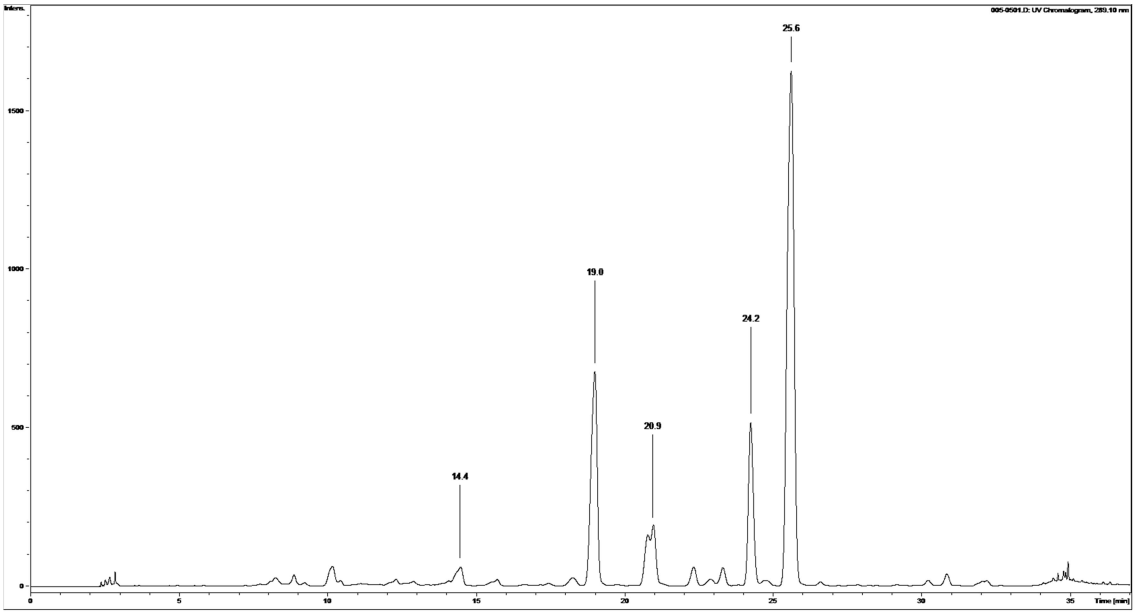

Figure S9g. HPLC chromatogram at λ = 289 nm of (**1m**+MsOH) and mass spectra of peaks at t^R = 14,4 (**1'm**) , t^R = 19,0 (**1m**) , t^R = 20,9 (**10m**) , t^R = 25,6 (**2m**)

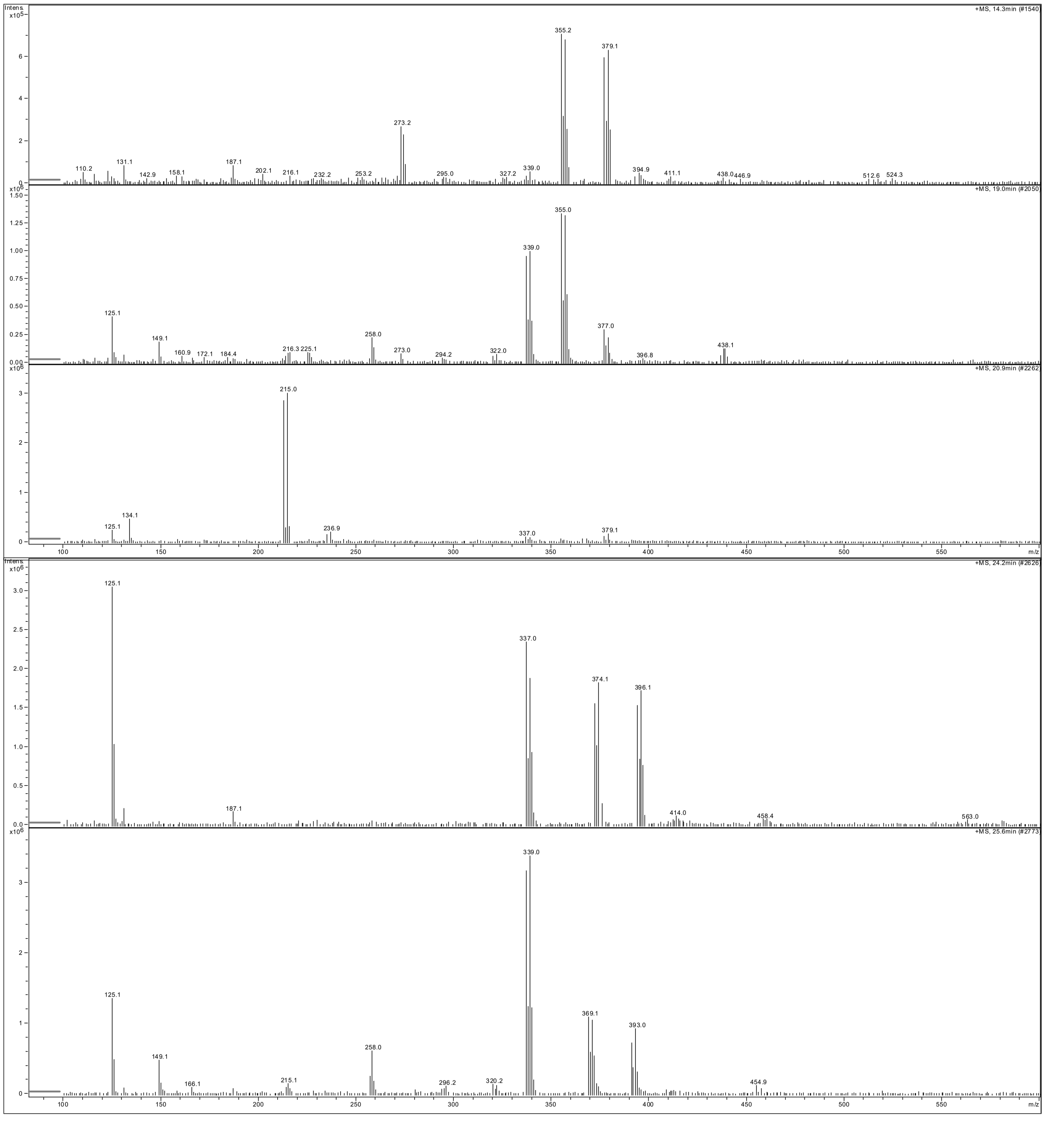

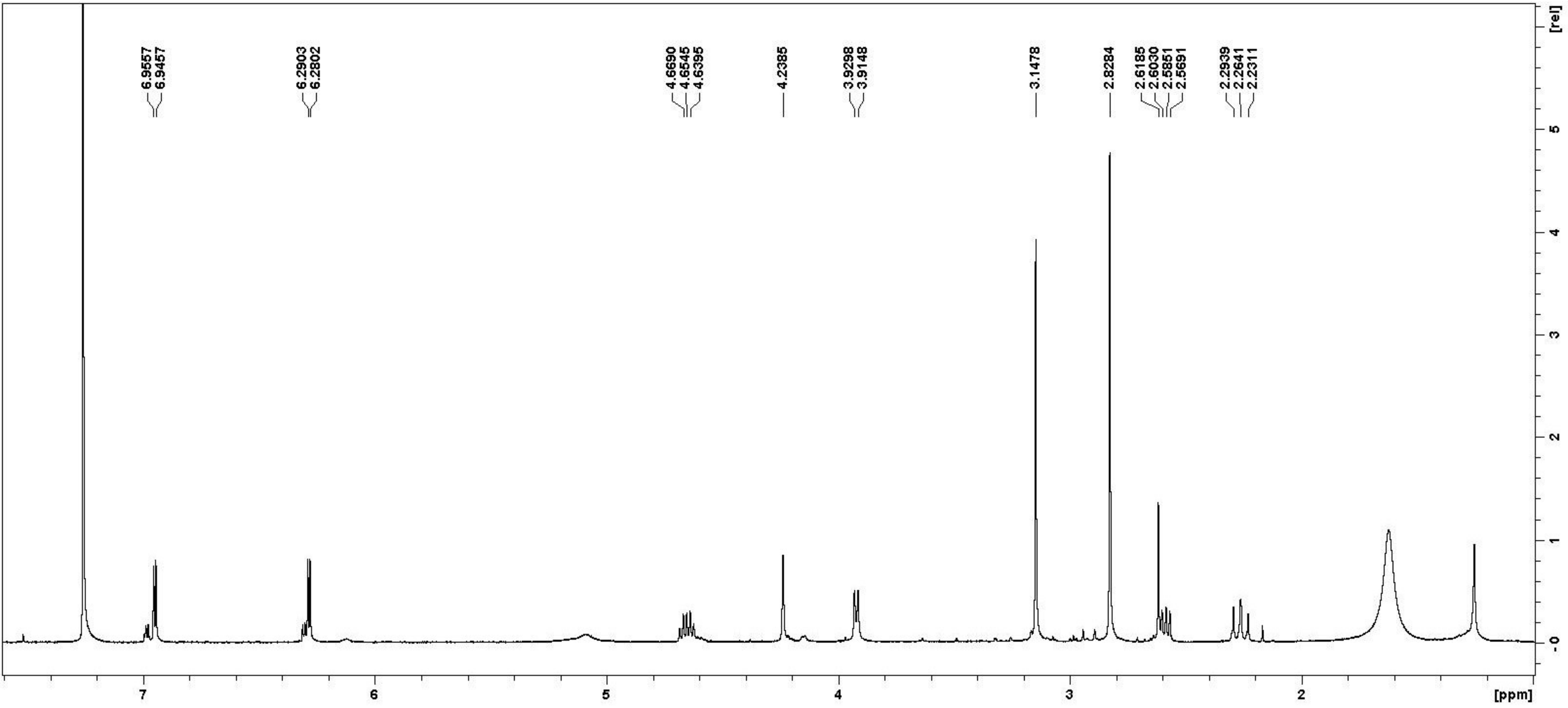

Figure S9h. – **1d** for reaction performed in the NMR tube

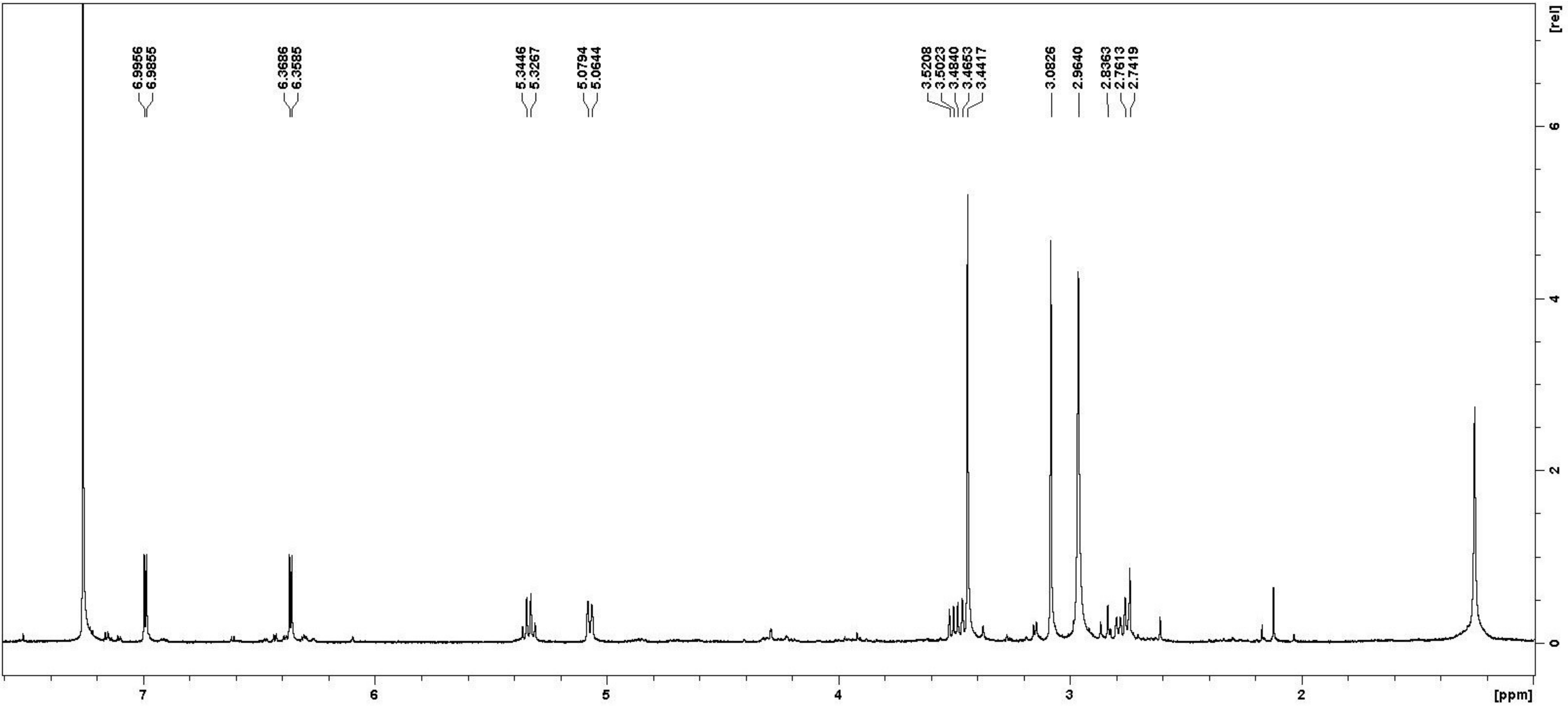

Figure S9i. – **1d** + MsOH in CDCl3 65°,40’ => **3d**

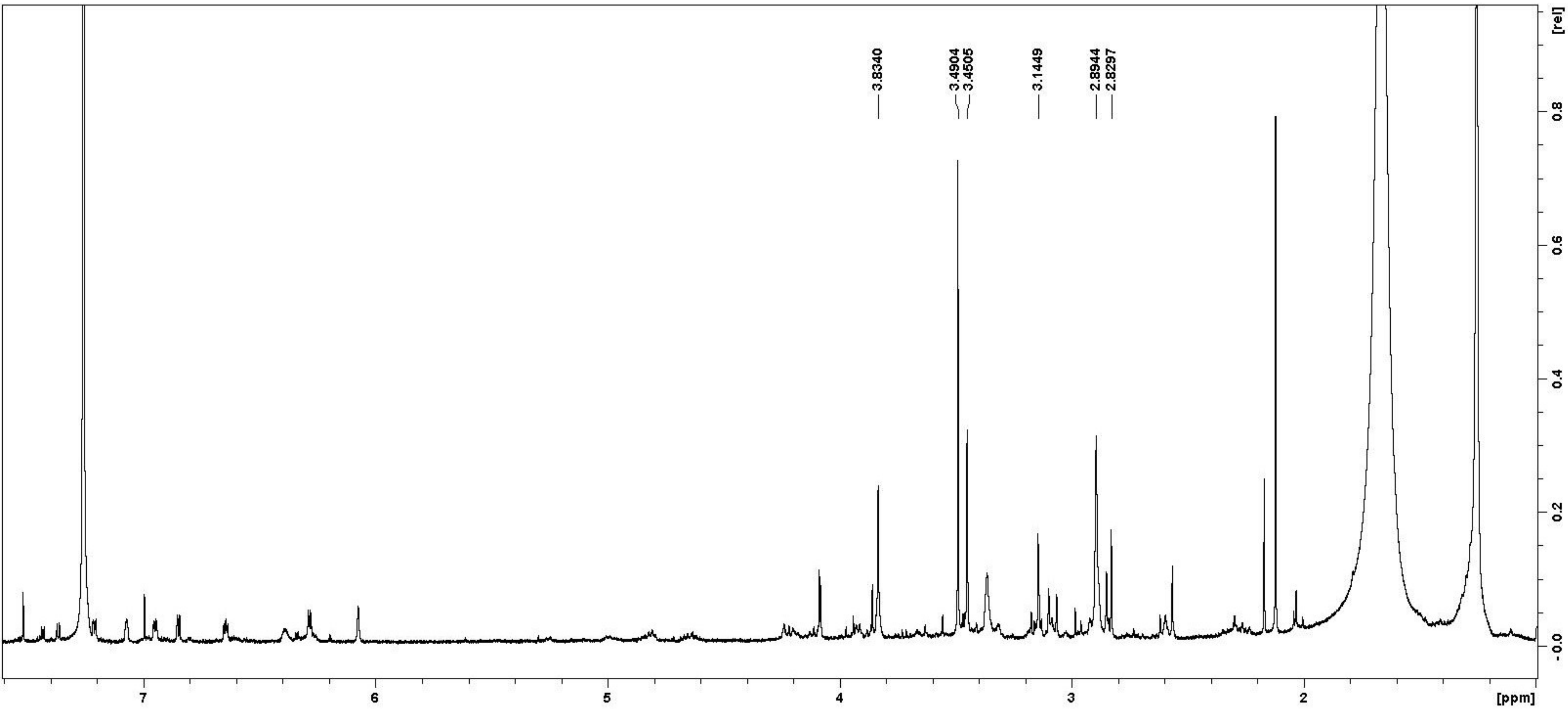

Figure S9j. – **1d** + MsOH in CDCl3 65°, 240’ than A21 => **5d**

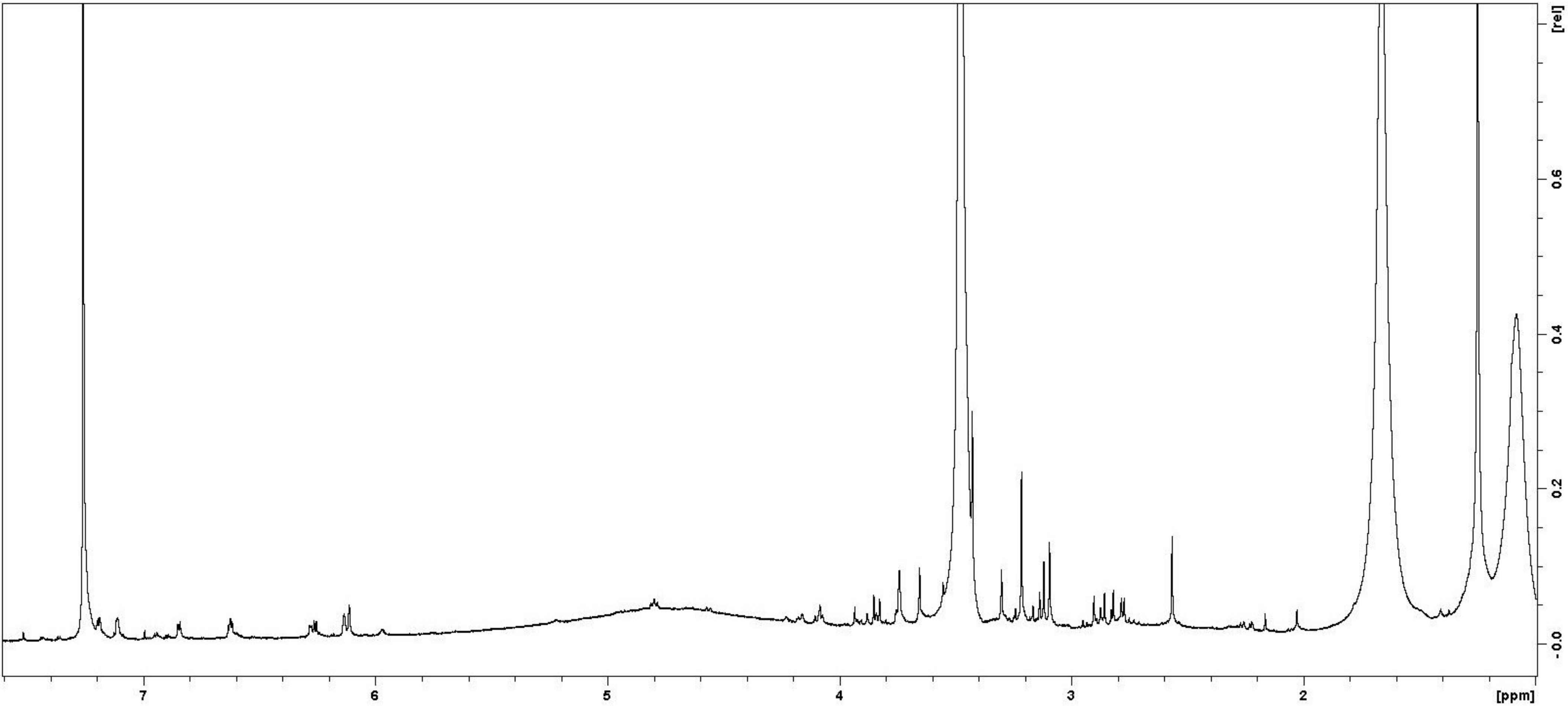

Figure S9k. – **2d** + MsOH in CDCl3 65°, 70' than A21 => **5d** for LC-MS

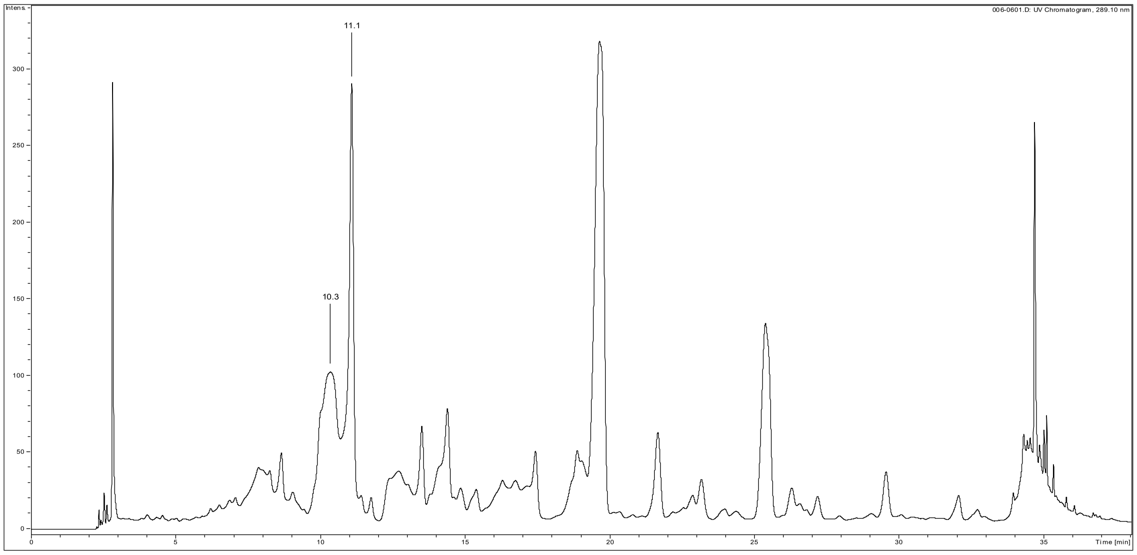

Figure S9l. HPLC chromatogram at λ = 289 nm of (**2d** +MsOH),
mass spectrum of peak at t_R = 19,6 (**2d**) and MS^2 fragmentation of peaks at t_R = 7,1 & 8,7 (**6d**), t_R = 10,4 & 11,3 (**5d**).

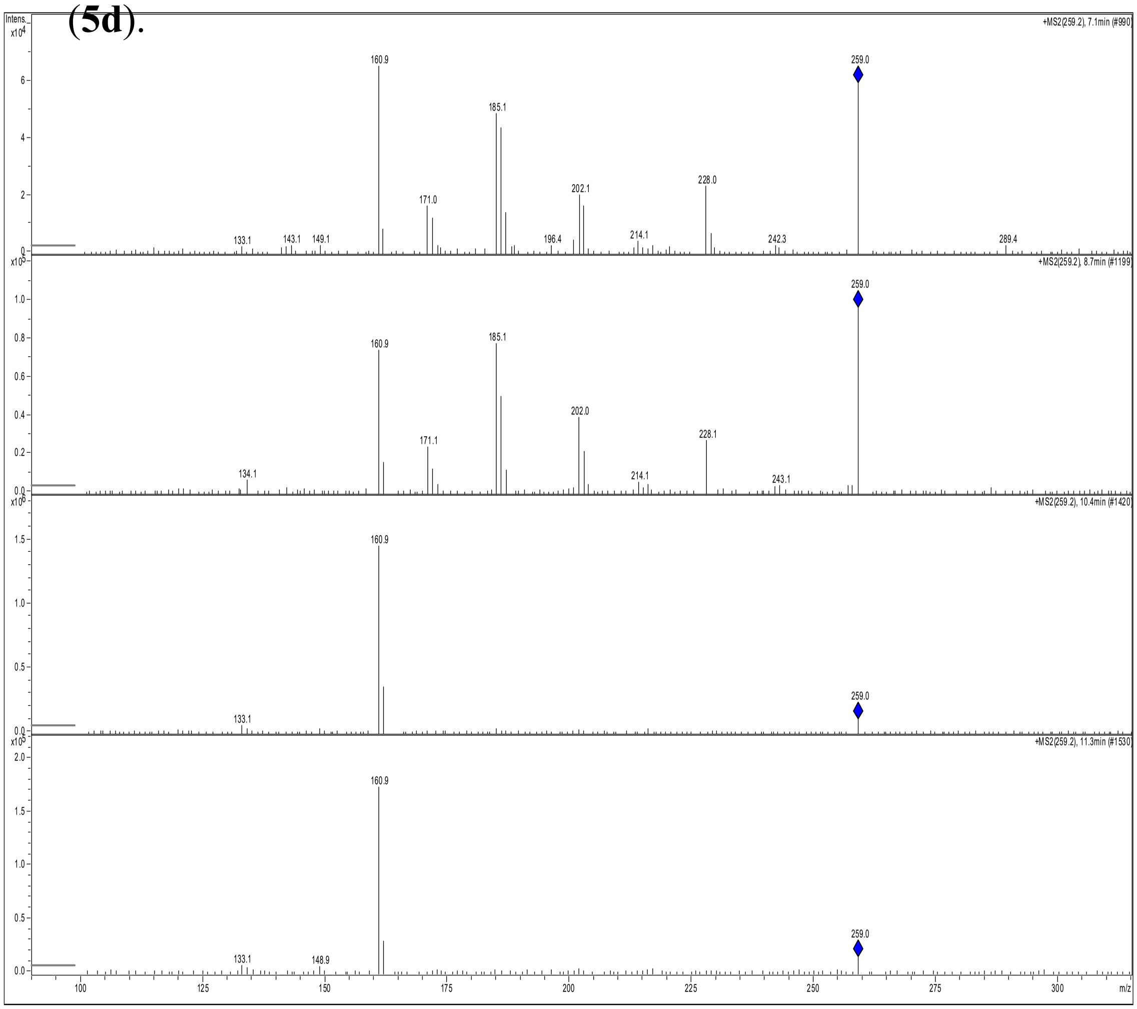

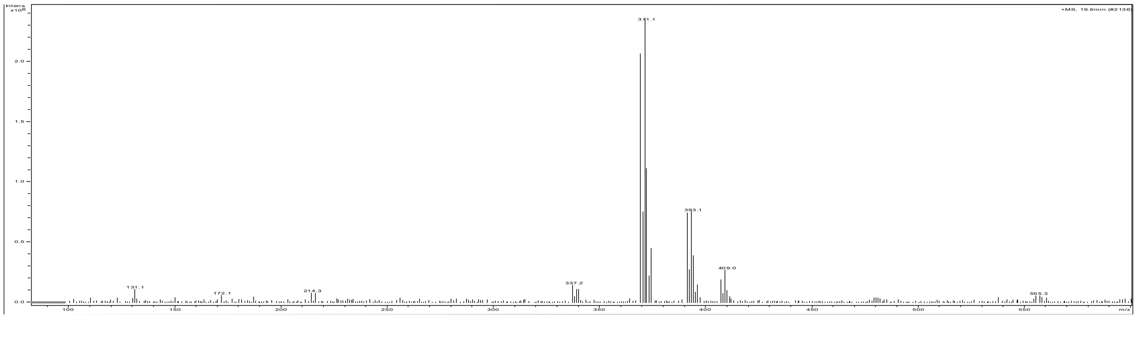

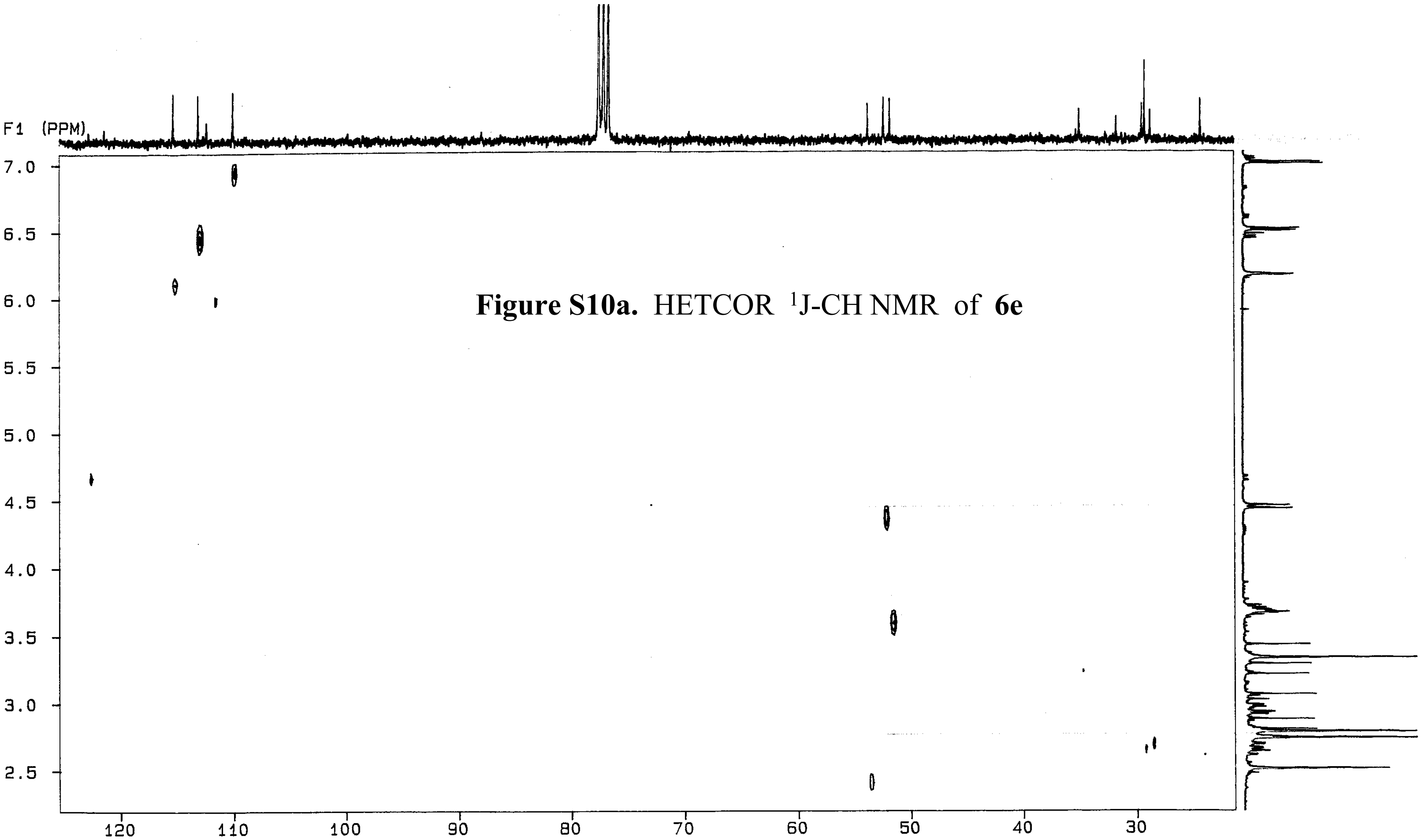

Figure S10a. HETCOR [1]J-CH NMR of **6e**

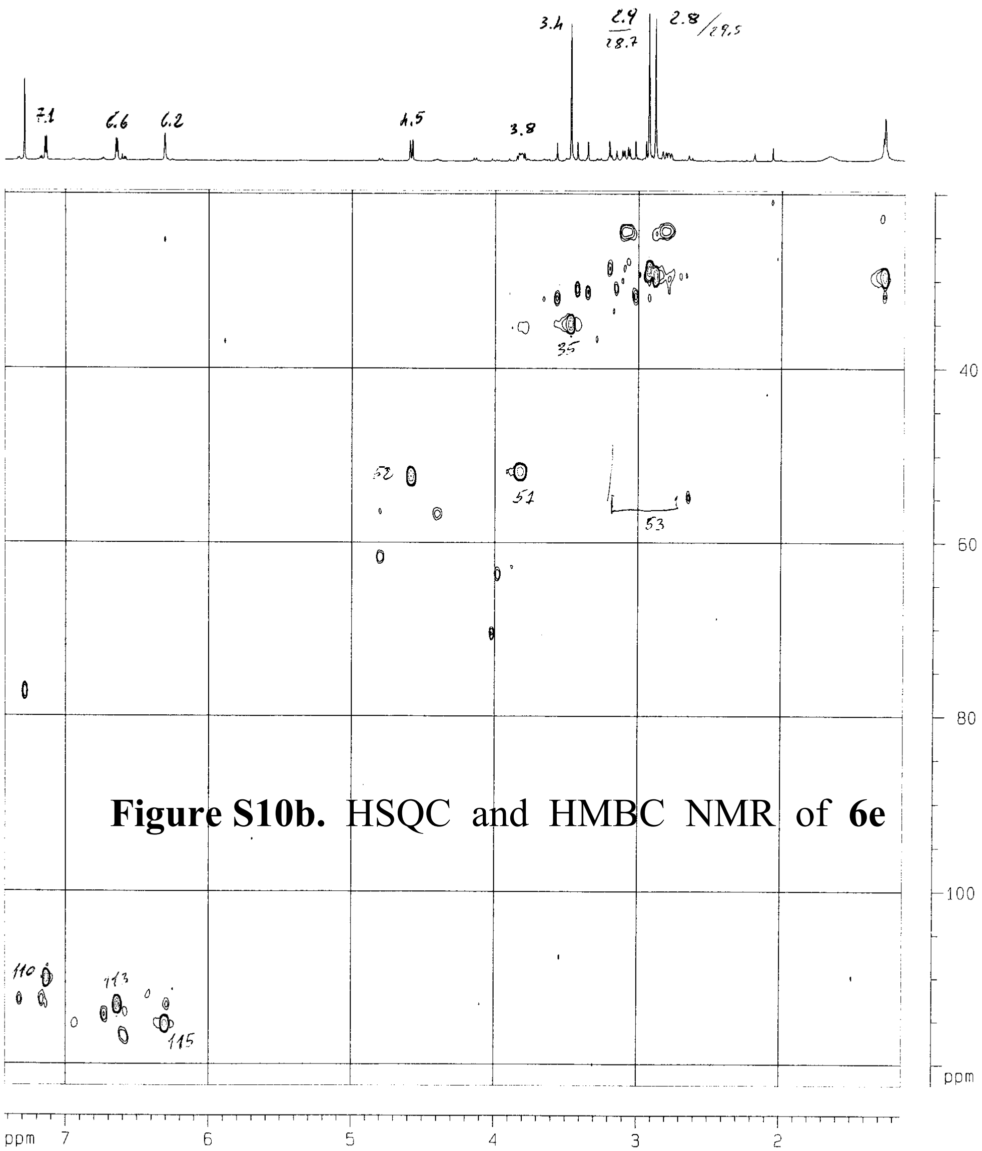

Figure S10b. HSQC and HMBC NMR of **6e**

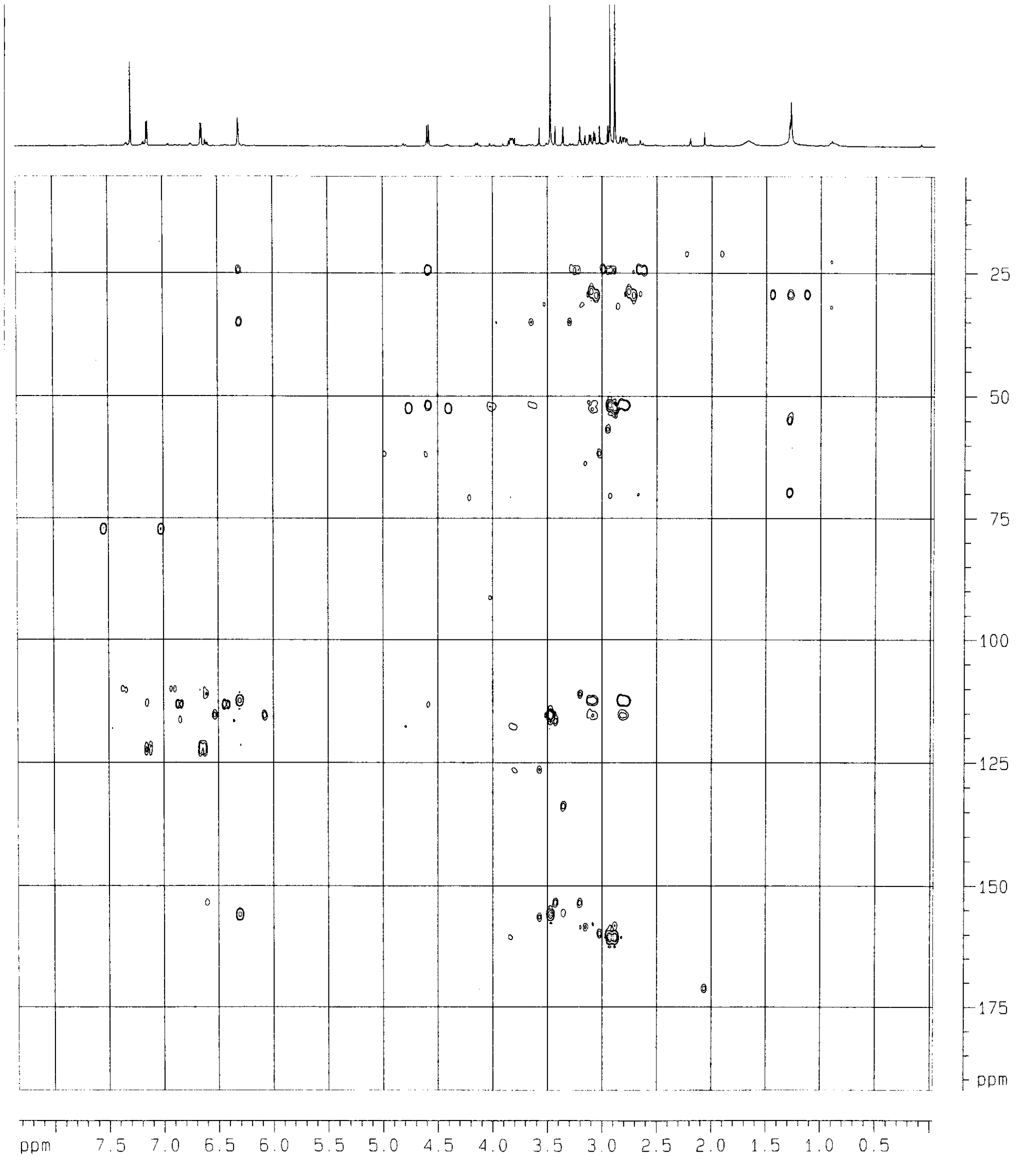

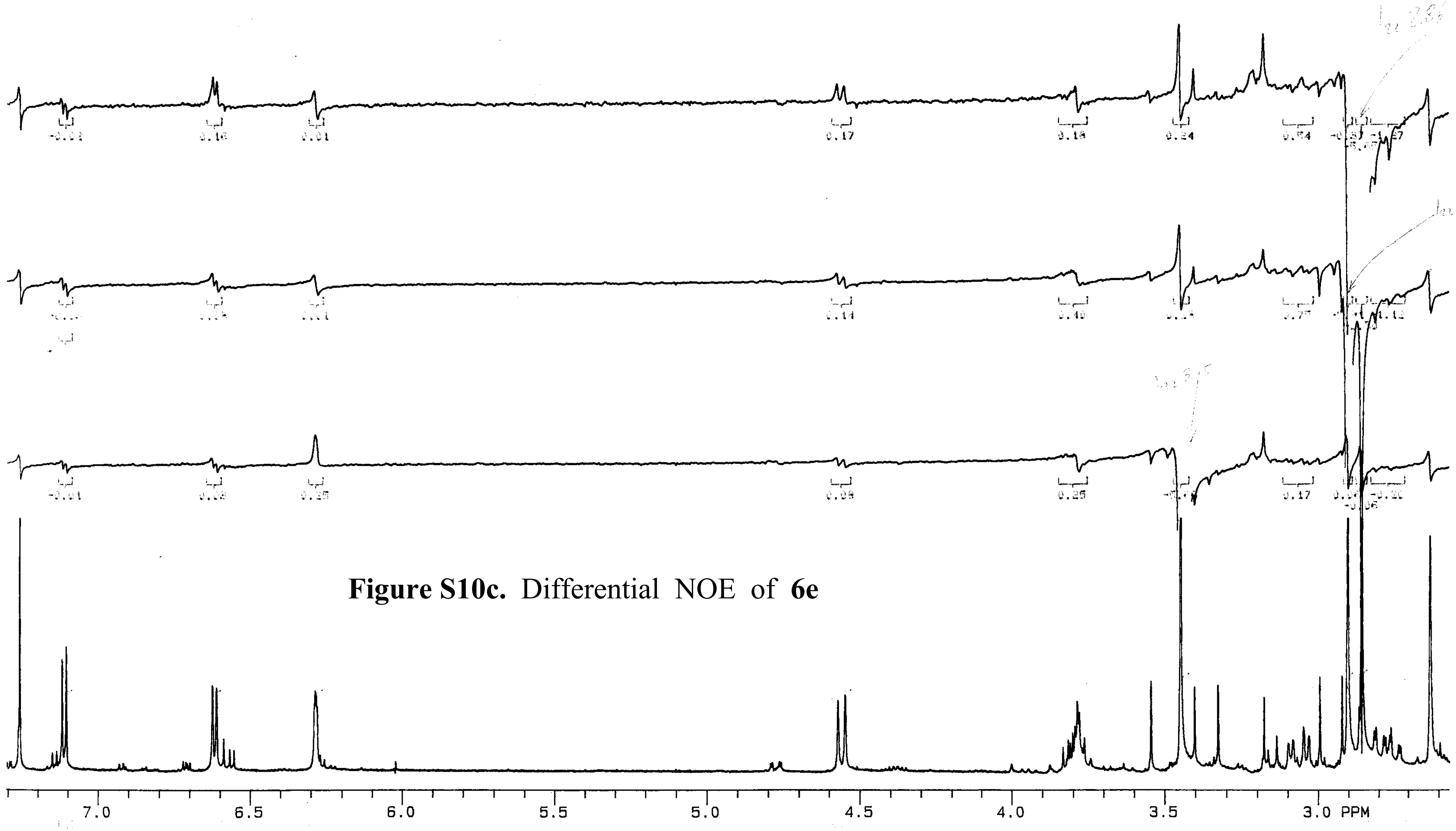

Figure S10c. Differential NOE of **6e**

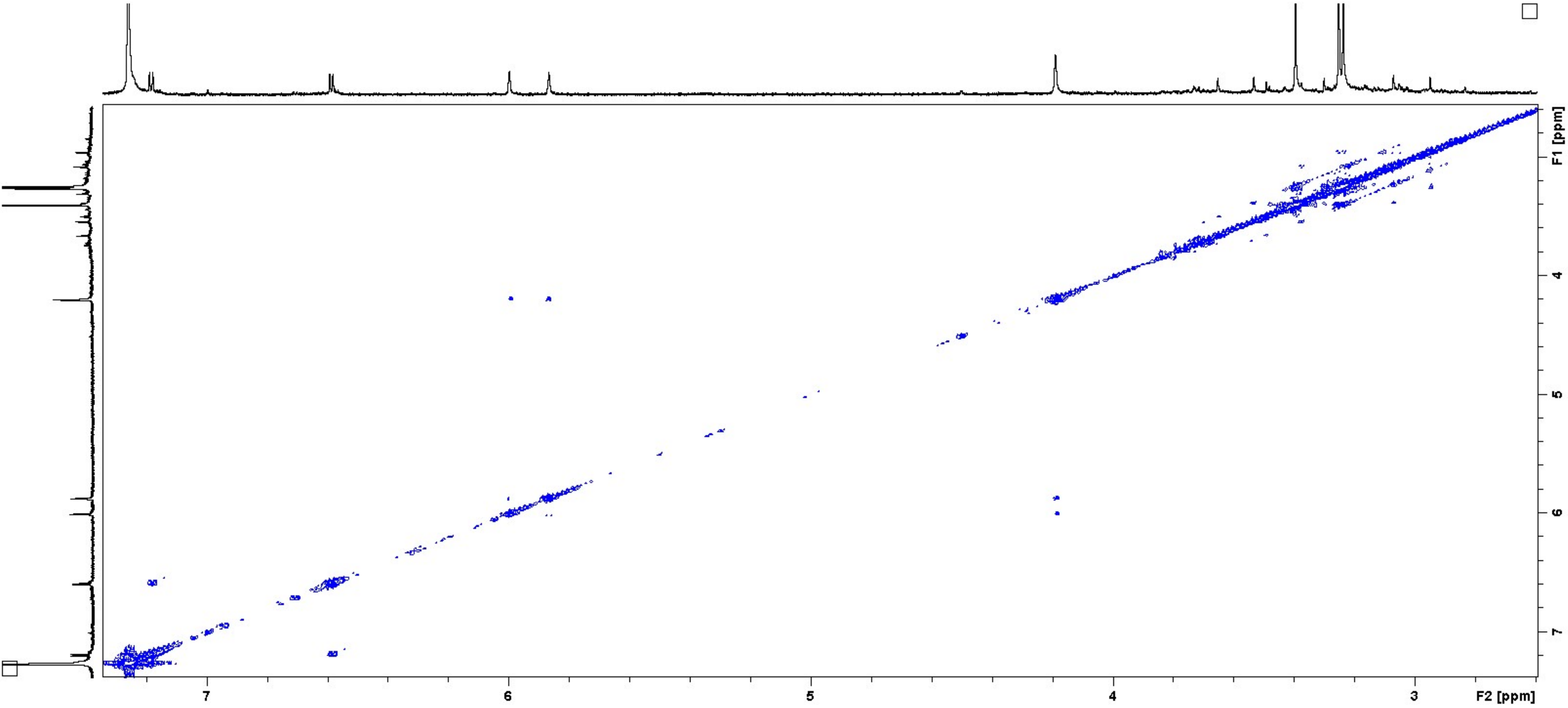

Figure S10d. – COSY spectrum of **4e**. See **S5g** for ^{1}H-NMR

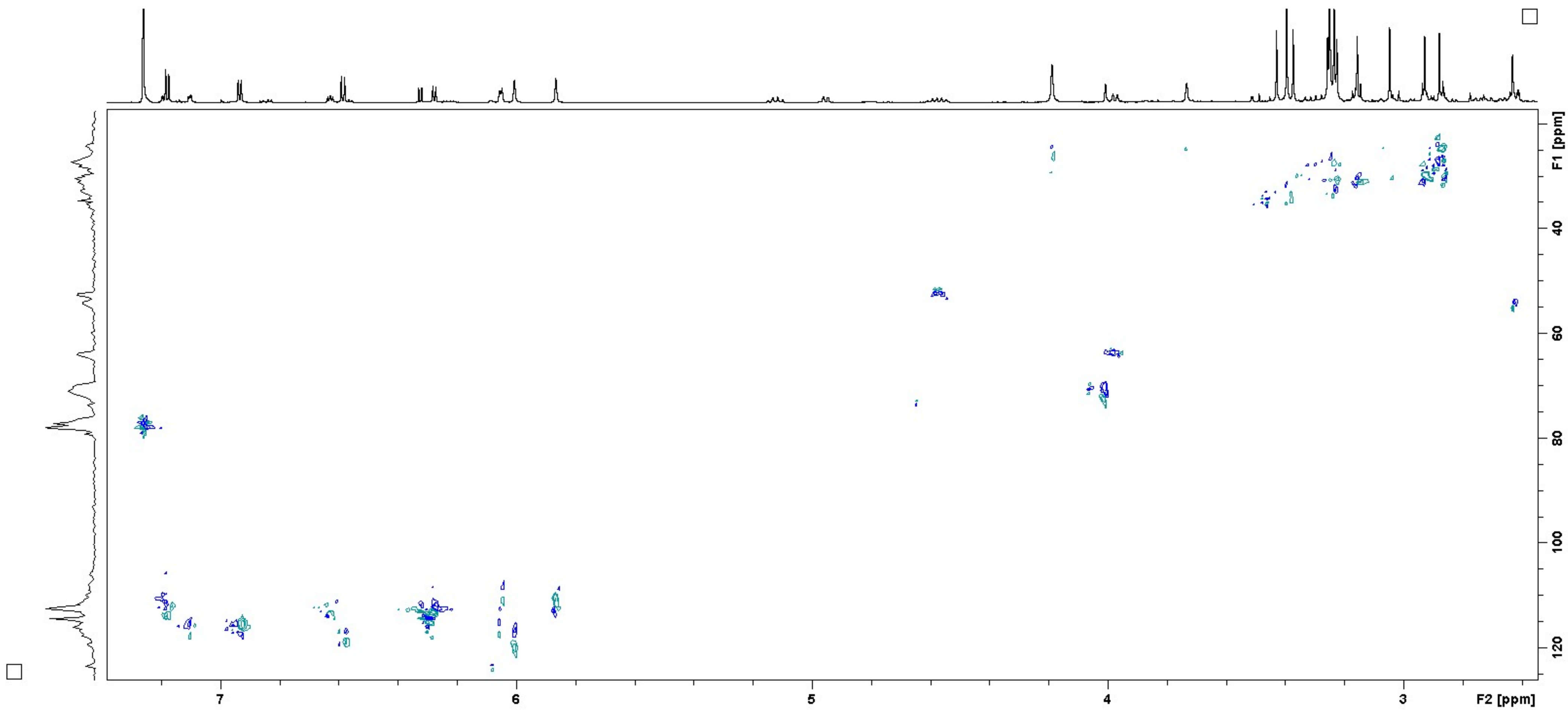

Figure S10e. – HSQC spectrum of **4e** (major component in the mixture).

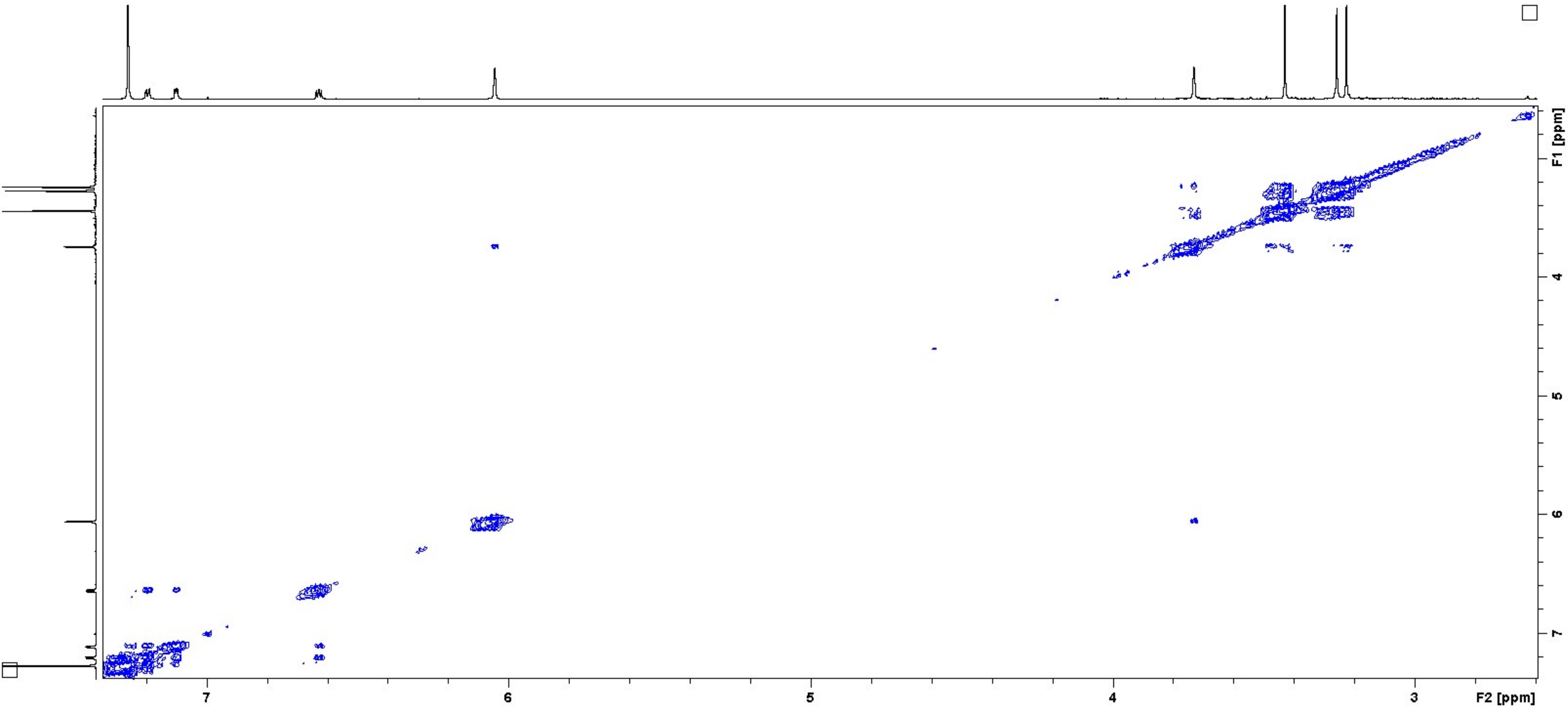

Figure S10f. – COSY spectrum of **5e**. See **S5h** for ^{1}H-NMR

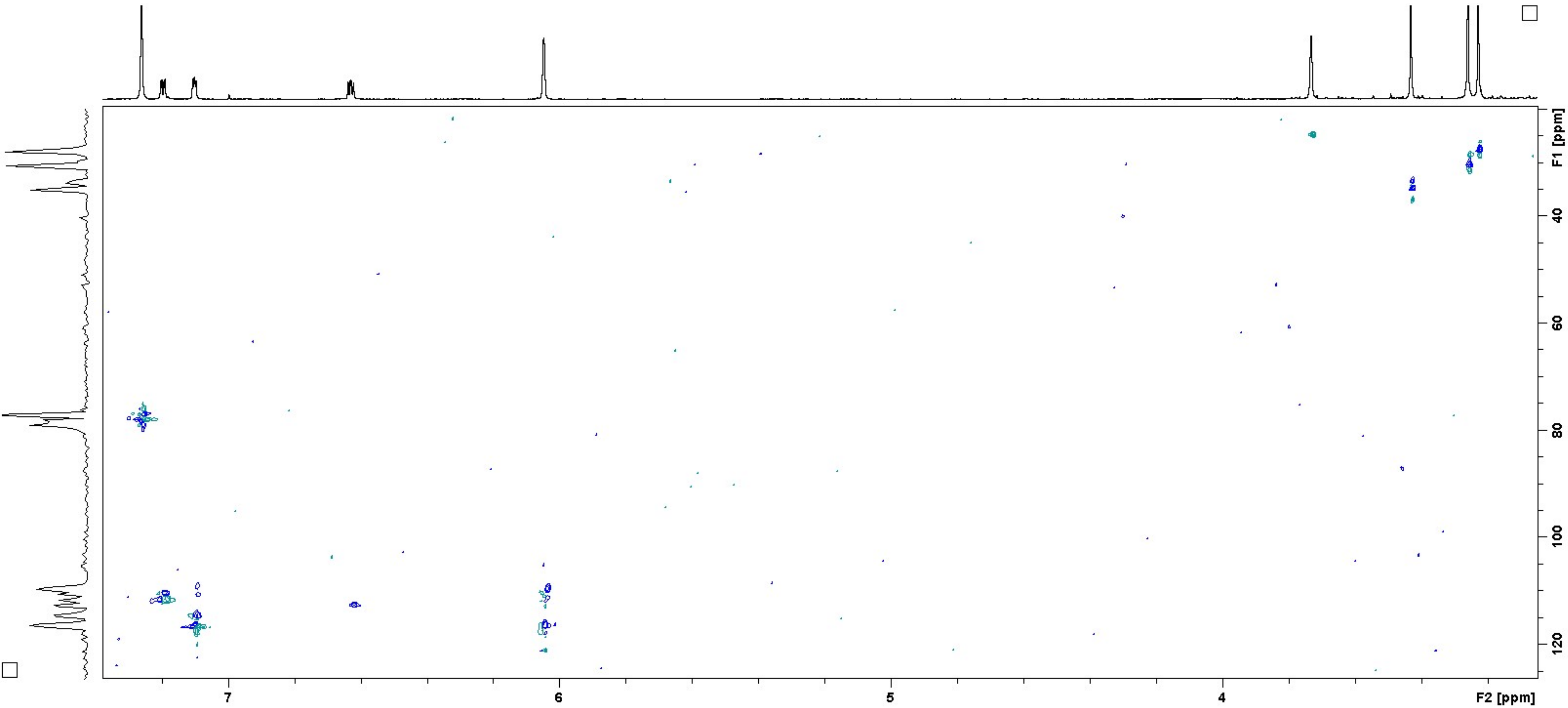

Figure S10g. – HSQC spectrum of **5e**.

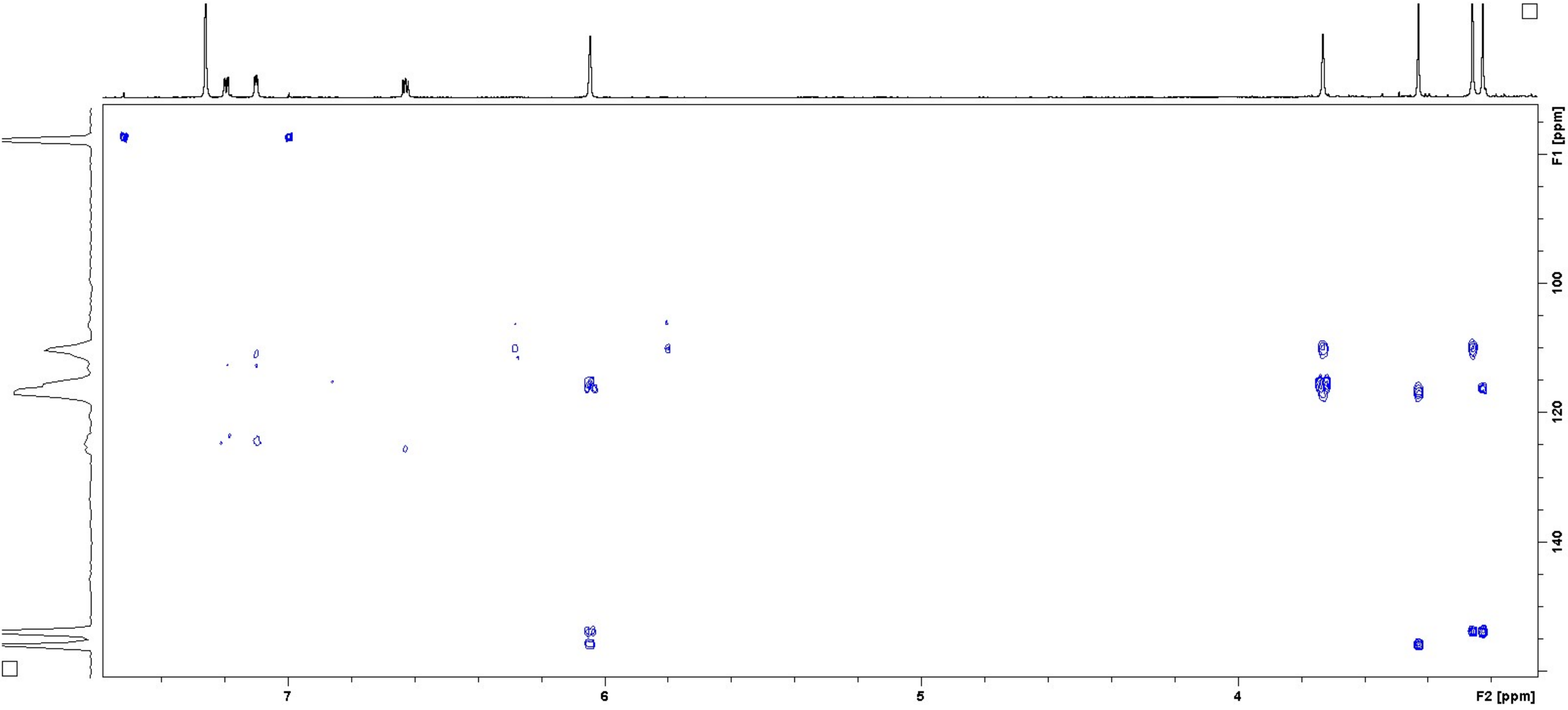

Figure S10h. – HMBC spectrum of **5e**.

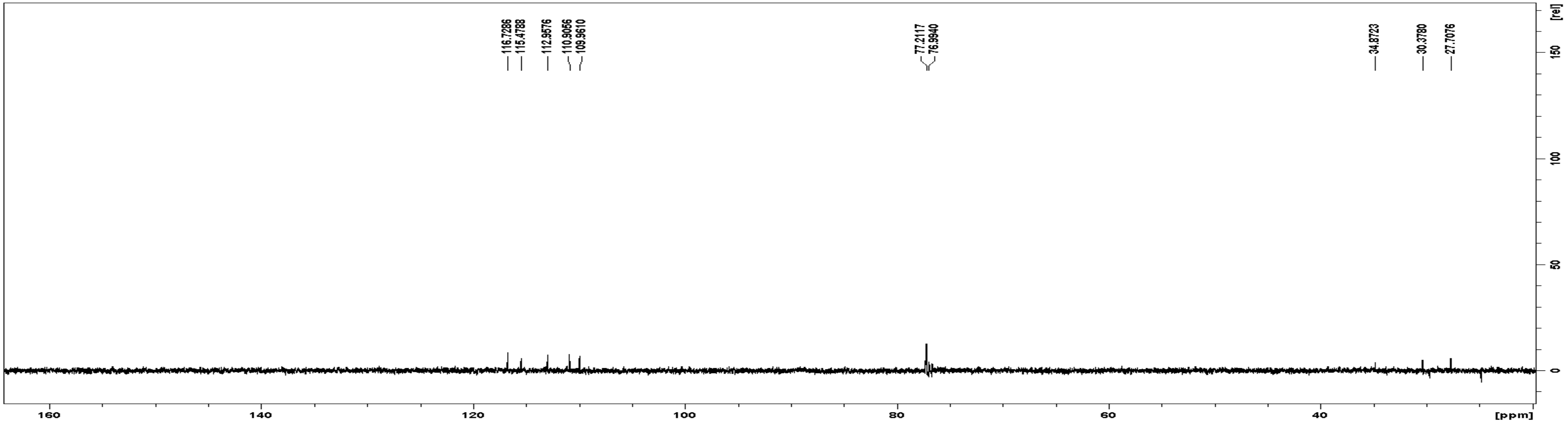

Figure S10i. - ↑ DEPT spectrum **5e** ↓ ^{13}C-NMR spectrum

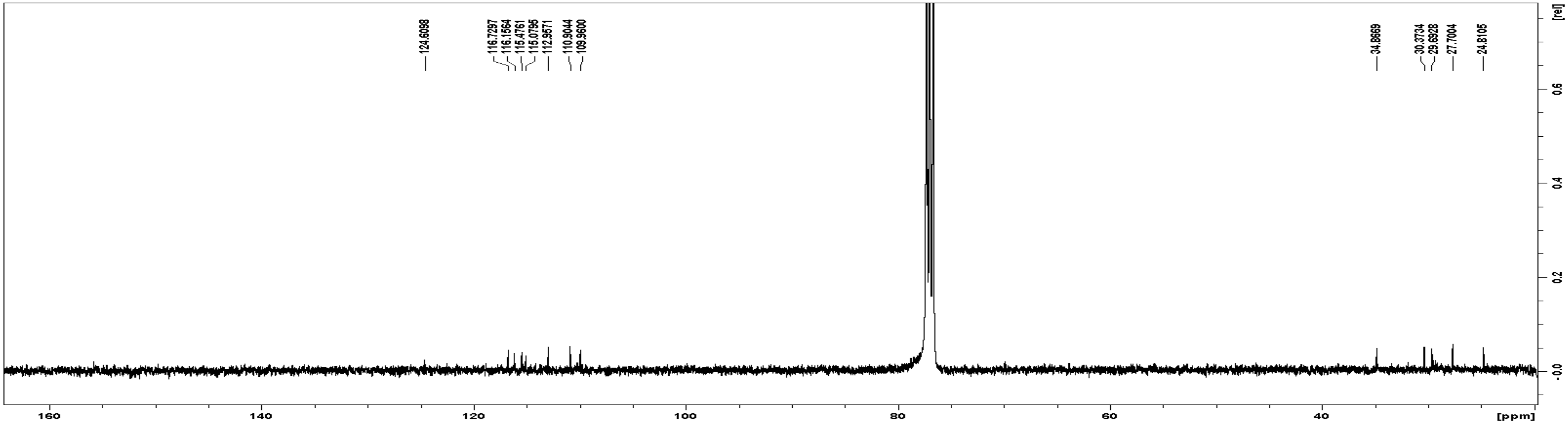

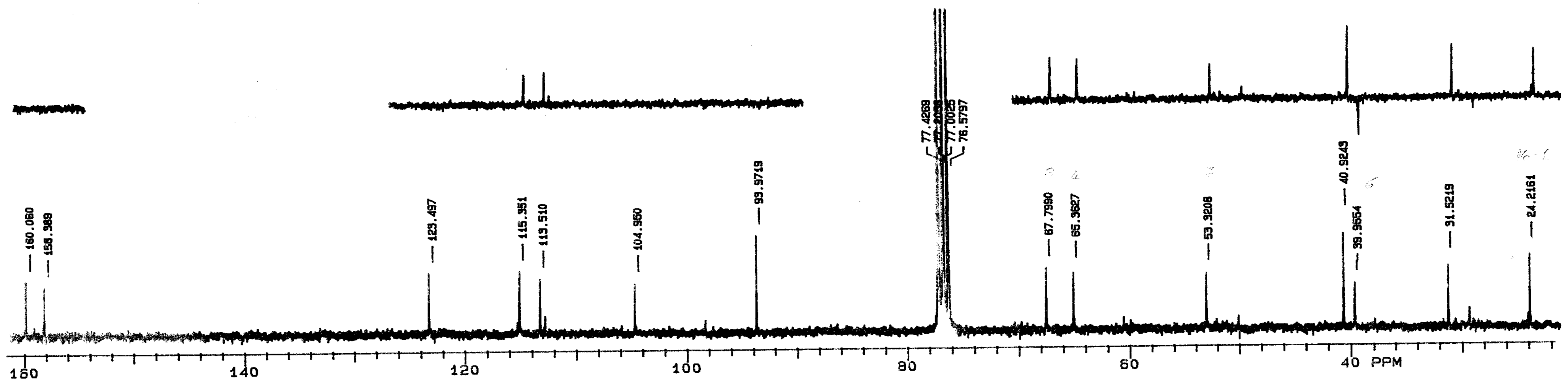

Figure S10j. - ↑ ^{13}C-NMR and DEPT spectra of **1d** ↓ ^{13}C-NMR and DEPT spectra of **2d**

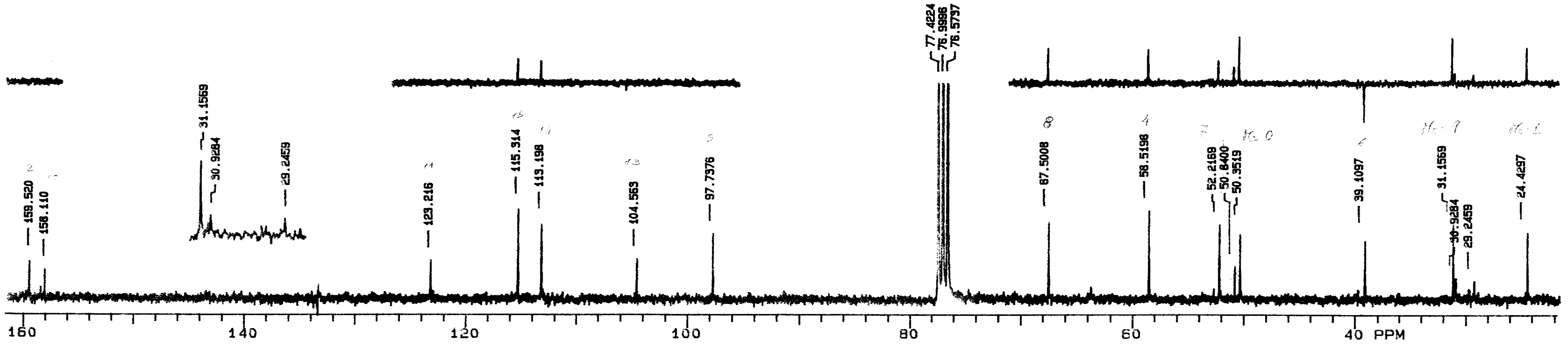

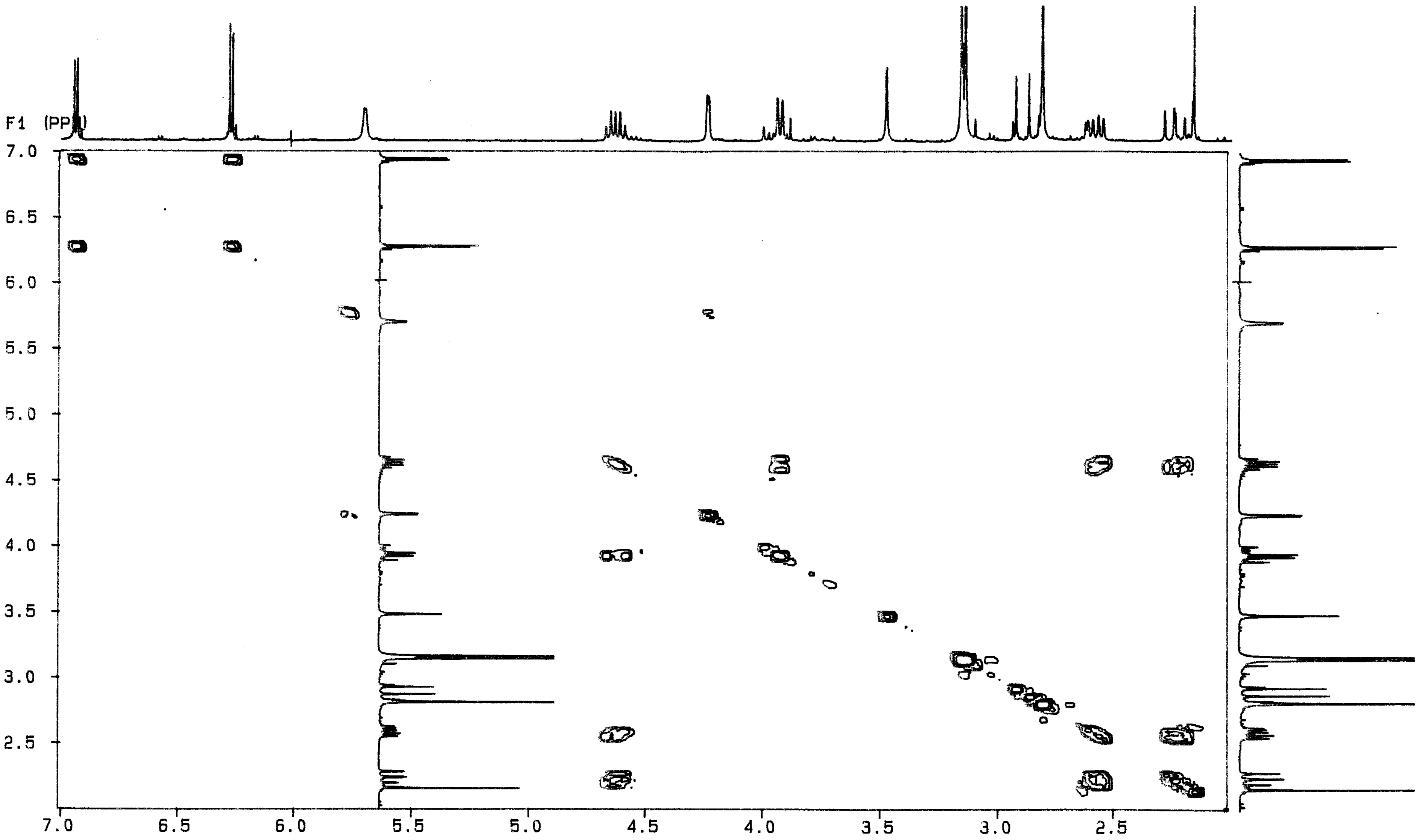

Figure S10k. – COSY spectrum of **2d**. See **1f** for ^{1}H-NMR

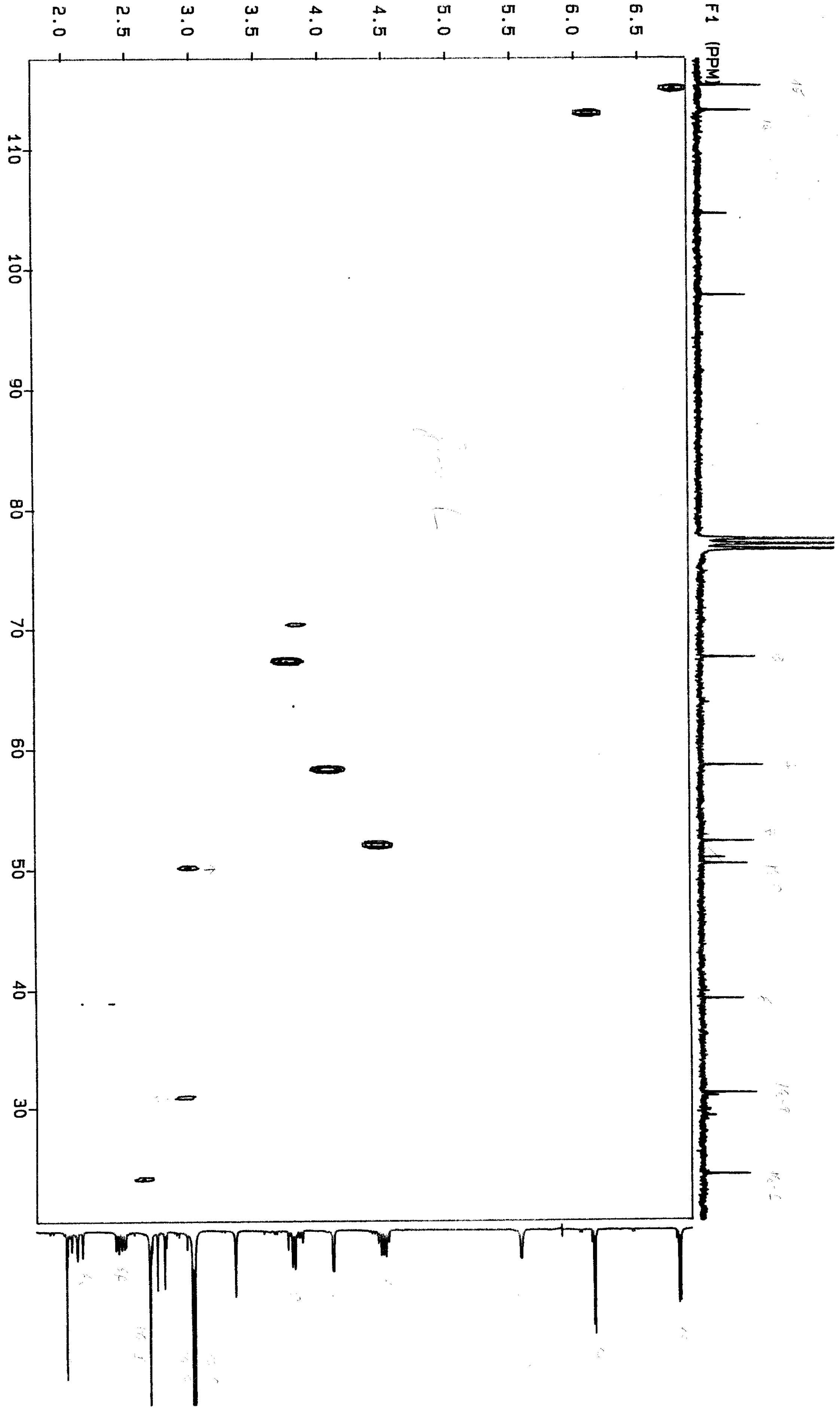

Figure S10l. – HETCOR ^{1}J-CH NMR spectrum of **2d**.

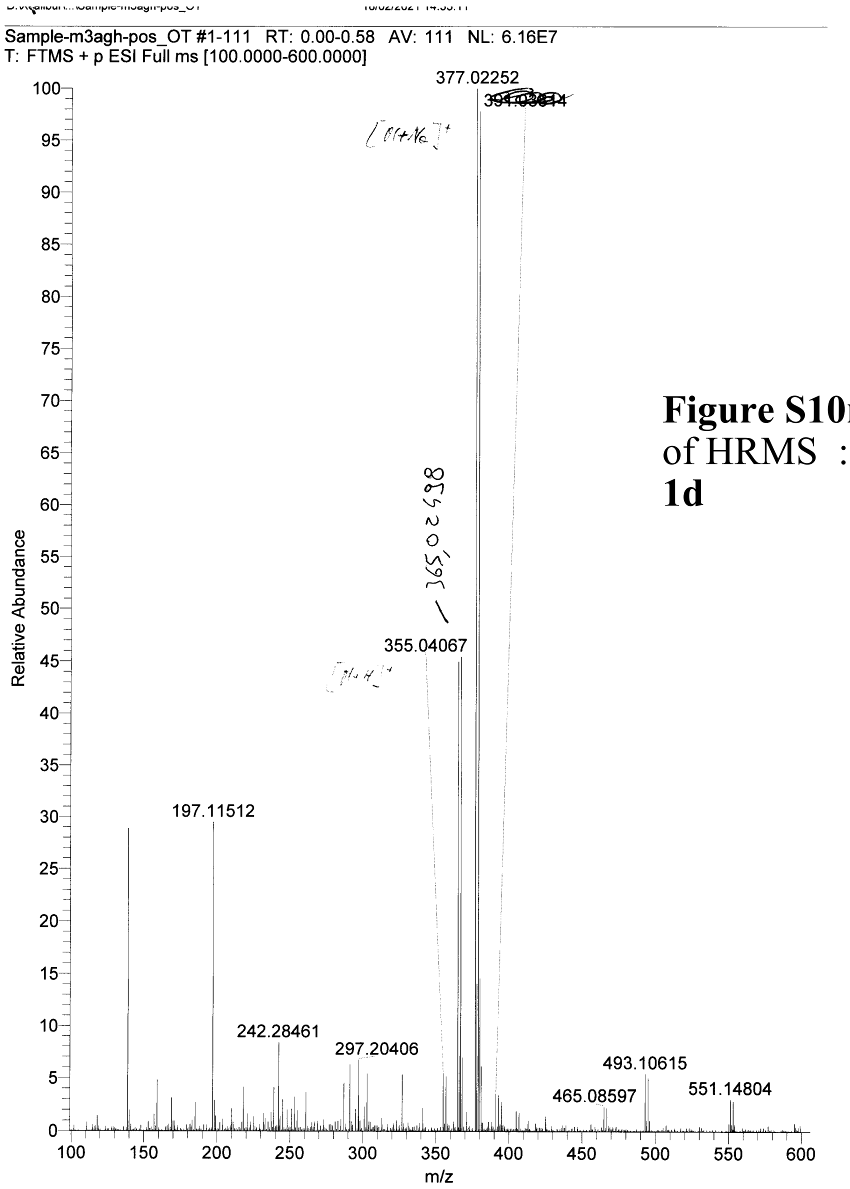

Figure S10m. COPY of HRMS : **1m** & **1d**

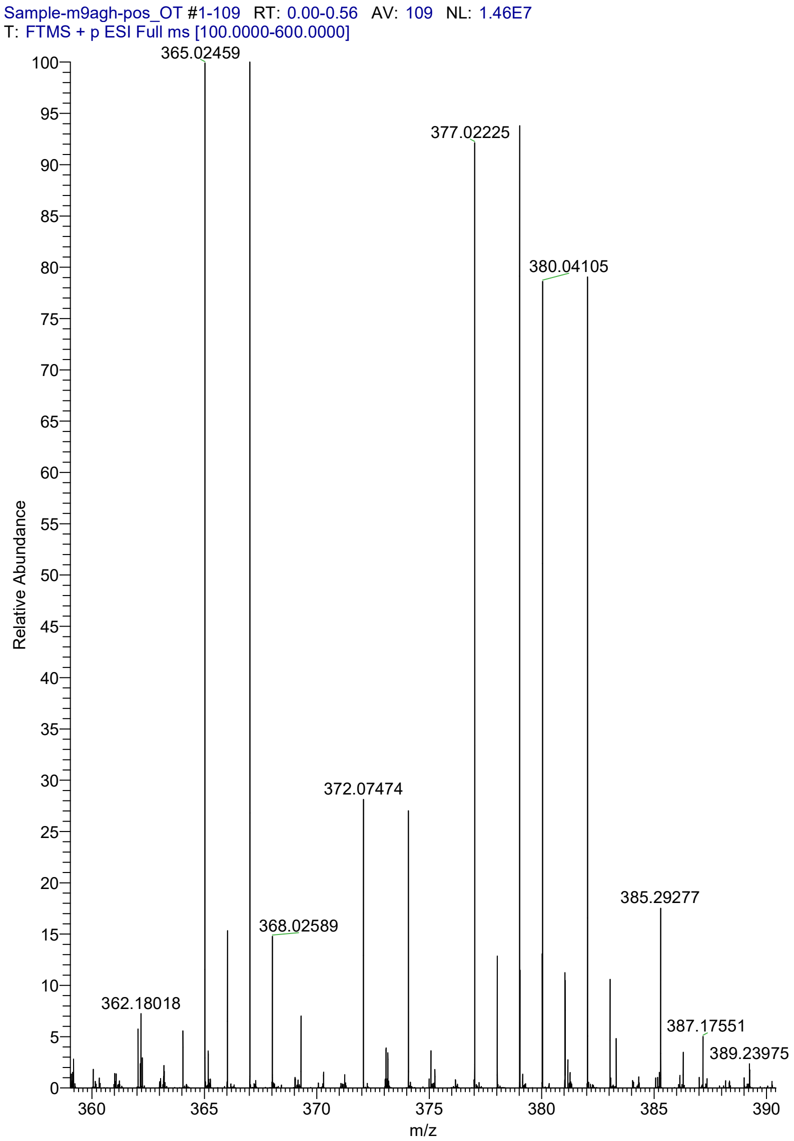

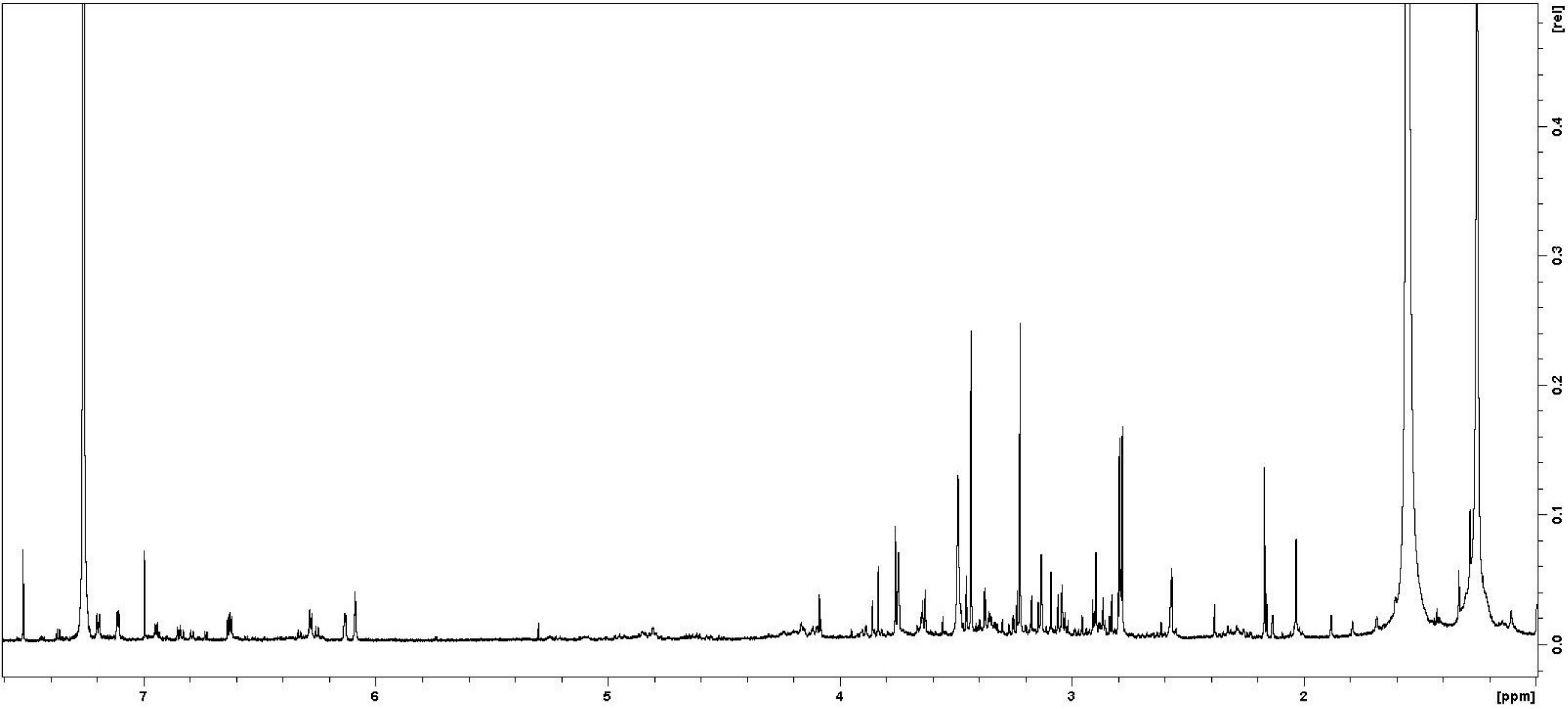

Figure S10n. – **1d** + MsOH in CDCl3 65°, 120' than A21 => **5d**

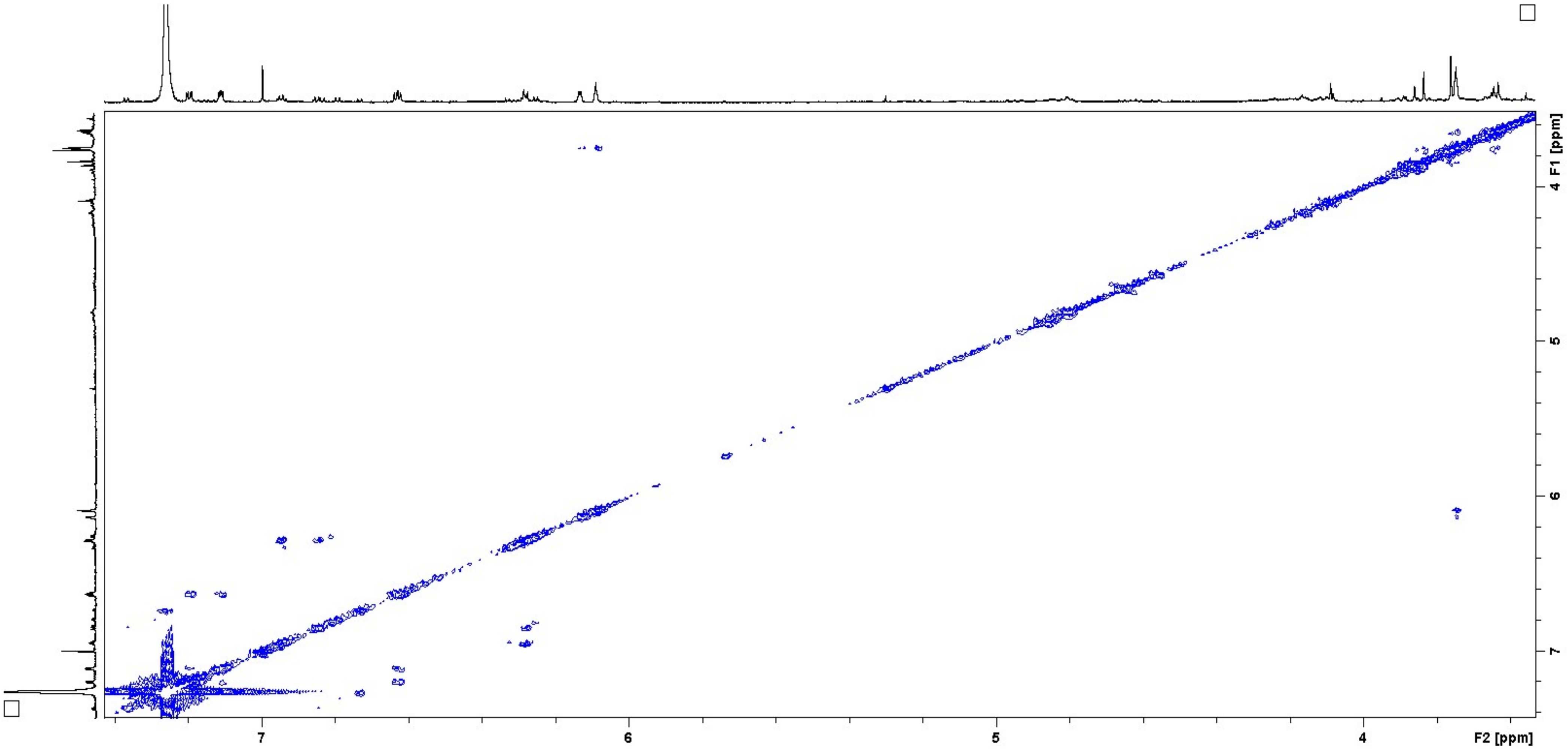

Figure S10o. – **5d** – cosy
